# Additive-Mediated Selective Oxidation of Alcohols to Esters via Synergistic Effect Using Single Cation Cobalt Catalyst Stabilized with Inorganic Ligand

**DOI:** 10.34133/2020/3875920

**Published:** 2020-01-23

**Authors:** Jingjing Wang, Han Yu, Zheyu Wei, Qi Li, Weimin Xuan, Yongge Wei

**Affiliations:** ^1^School of Chemical and Environmental Engineering, Shanghai Institute of Technology, Shanghai 201418, China; ^2^Key Lab of Organic Optoelectronics & Molecular Engineering of Ministry of Education, Department of Chemistry, Tsinghua University, Beijing 100084, China; ^3^State Key Laboratory of Natural and Biomimetic Drugs, Peking University, Beijing 100191, China; ^4^College of Chemistry, Chemical Engineering and Biotechnology, Donghua University, Shanghai 201620, China

## Abstract

The direct catalytic oxidation of alcohols to esters is very appealing, but the economical-friendly catalysis systems are not yet well established. Herein, we show that a pure inorganic ligand-supported single-atomic cobalt compound, (NH_4_)_3_[CoMo_6_O_18_(OH)_6_] (simplified as CoMo_6_), could be used as a heterogeneous catalyst and effectively promote this type of reaction in the presence of 30% H_2_O_2_ using KCl as an additive. The oxidative cross-esterification of various alcohols (aromatic and aliphatic) could be achieved under mild conditions in nearly all cases, affording the corresponding esters in high yields, including several drug molecules and natural products. Detailed studies have revealed that chloride ion is able to bind to the CoMo_6_ to form a supramolecular dimer 2(CoMo_6_∙Cl), which can effectively catalyze the reaction *via* a synergistic effect from chloride ion and CoMo_6_. Mechanism studies and control reactions demonstrate that the esterification proceeds *via* the key oxidative immediate of aldehydes.

## 1. Introduction

**Figure 1:**
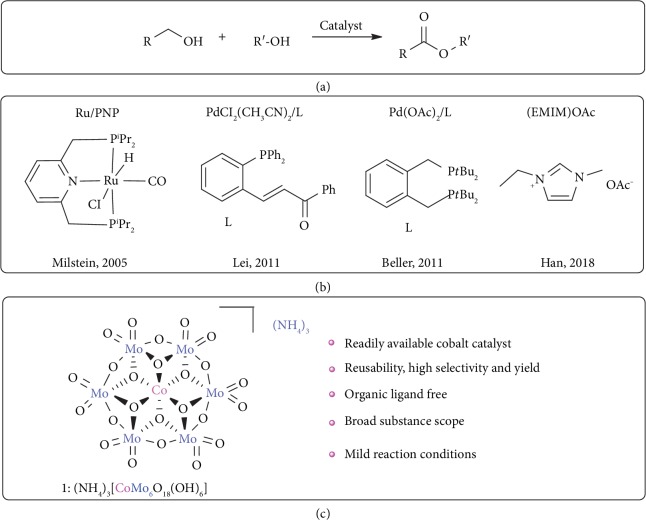
Catalytic systems for oxidation of alcohols to esters: (a) oxidation of alcohols to esters; (b) organometallic complexes or organic catalysts (previous work); (c) inorganic ligand-supported cobalt catalyst (this work).

Ester groups are prevalent in diverse natural products, pharmaceuticals, polymers, fine chemicals, and widely used synthons [1–3]. In general, esters are synthesized by the reaction of carboxylic acids or activated derivatives (acyl chlorides and anhydrides) with alcohols. Such reactions require multiple steps and will often generate a large quantity of undesired byproducts [4]. Therefore, the development of new, environmentally benign and atom-efficient methods without using stoichiometric amount of condensing reagents and activators has attracted much interest. In this respect, an attractive approach concerns the direct transformation of alcohols to esters under catalytic conditions using green oxidants such as O_2_ and H_2_O_2_ (Figure 1(a)).

Both homogeneous and heterogeneous catalytic systems have been well established for the highly efficient oxidation of alcohols to esters. Some of the most effective examples utilize metal-based homogeneous catalysts supported by an organic ligand, such as ruthenium [5–10], palladium [11–14], gold [15], iridium [16, 17], and rhodium [18], or rely on the use of toxic halide-based oxidants [19]. Heterogeneous catalysts generally employ functional ligands to modulate the catalytic performance (Figure 1(b)). The former homogeneous catalysts have been reported to have good generality but require the use of noble metals together with complex and expensive organic ligands which are susceptible to oxidative self-degradation and often require harsh reaction conditions at elevated temperature and pressure [20, 21]. Heterogeneous catalysts can provide the benefits of easy separation from the reaction mixture, but the catalysts are not commercially available or otherwise are not easily accessible [22–26]. Very recently, in 2018, Gowrisankar and coworkers [27] reported an ionic liquid as catalyst for the oxidation of alcohols to esters, but the method required high pressures (10 or 20 bar) and lacked generality. Therefore, the development of promising catalytic systems towards direct conversion of alcohols to esters, which utilize robust and cheap base-metal catalysts under mild conditions in the presence of green oxidants, is highly desired.

Polyoxometalates (POMs) [28, 29], a class of discrete metal oxides, are regarded as inorganic ligands that are able to coordinate to metal centers and quite different from traditional transition metal compounds. They not only feature reversible redox and strong acidities at the atomic or molecular levels but also exhibit high tolerance towards oxidative degradation and hydrolysis [30–33]. Recently, our group showed that Anderson-type POMs can serve as the inorganic ligand-supported metal catalysts to promote the aerobic oxidation of aldehydes to carboxylic acids [34], amines to imines [35], and alcohols to aldehydes [36]. This type of purely inorganic catalysts features a ring-like structure consisted of six edge-sharing {Mo^VI^O_6_} inorganic octahedral units supporting a central metal site (Figure 1(c)); this arrangement on the one hand reinforces the Lewis acidity of the central catalytic metal centers, on the other hand makes full use of the edge-sharing {MoO_6_} scaffolds as versatile inorganic ligands to encompass various catalytic sites, thus may bring novel features that cannot be attained by the organic counterparts used in classic organometallic catalysts. Herein, we report a Co(III)-based Anderson-type catalyst 1, (NH_4_)_3_[CoMo_6_O_18_(OH)_6_] (simplified as CoMo_6_, Figure 1(c), Figures [Supplementary-material supplementary-material-1]), which can effectively catalyze the oxidative cross-esterification of a variety of alcohols to afford the corresponding esters directly with KCl as addictive and H_2_O_2_ as green oxidant. Mechanistic insights were deduced based upon control reactions and the detection of key intermediate by NMR. Compared with previously reported homogeneous catalysts, the purely inorganic 1 avoids the use of complicated/sensitive organic ligands and noble metals. More importantly, due to the high stability, 1 can be recovered and reused for six times while almost maintain the same activity.

## 2. Results

The catalysis study started with the oxidative coupling of benzyl alcohol and methanol using 30% H_2_O_2_ as oxidant to evaluate the effectiveness of the cobalt catalyst (Tables [Supplementary-material supplementary-material-1]). To our delight, the targeted product methyl benzoate could be obtained with excellent selectivity and yield (Table 1, entry 1). The byproduct benzyl formate was not observed by GC-Ms. However, in the absence of cat. 1, trace amounts of methyl benzoate were detected under the same condition (Table 1, entry 2), indicating the key role of the cobalt catalyst. If the catalysis was carried out under the N_2_ atmosphere, the targeted ester was acquired in a yield <5% (Table 1, entry 3), demonstrating that H_2_O_2_ was essential for the oxidative coupling. With O_2_ (1 atm) as oxidant, methyl benzoate could be produced as well, albeit in lower yield (20%) even after 48 h; this may arise from the lower activity of molecular O_2_ (Table 1, entry 4). Meanwhile, when Co(NO_3_)_2_·6H_2_O or (NH_4_)_6_Mo_7_O_24_=(NH_4_)_6_[MoMo_6_O_18_(O)_6_] (isostructural Anderson-type cluster with the central Co being replaced by Mo) was utilized as the catalyst, trace quantities of the product were formed even after 48 h (Table 1, entries 5 and 6). However, when a mixture of 1.0 mol% Co(NO_3_)_2_·6H_2_O and 1.0 mol% of (NH_4_)_6_Mo_7_O_24_ was employed for the reaction, the desired ester was produced but in a low yield (Table 1, entry 7). These results clearly show that cat. 1 is indispensable and account for the superior oxidative efficiency in all the components. Moreover, additives also played an important role in the direct oxidative conversion, in which KCl (0.2 equiv) showed the best performance (Tables [Supplementary-material supplementary-material-1] and [Supplementary-material supplementary-material-1]). The yield of the desired product was dramatically reduced to 37% in the absence of KCl (Table 1, entry 8). This may be ascribed to the improved efficiency of electron transfer mediated by the chloride ion [37, 38]. Afterwards, the temperature, reaction time, and the type of oxidant were screened. The best yield can be obtained when using 2.0 equiv H_2_O_2_ as the oxidant at 70°C for 36 h ([Supplementary-material supplementary-material-1]). Besides, we also investigated the catalytic activities of other Anderson-type catalysts bearing different central metal atoms. It is shown that all these compounds can promote the transformation of alcohol to ester while cat. 1 exhibited the best performance ([Supplementary-material supplementary-material-1]).

**Figure 2:**
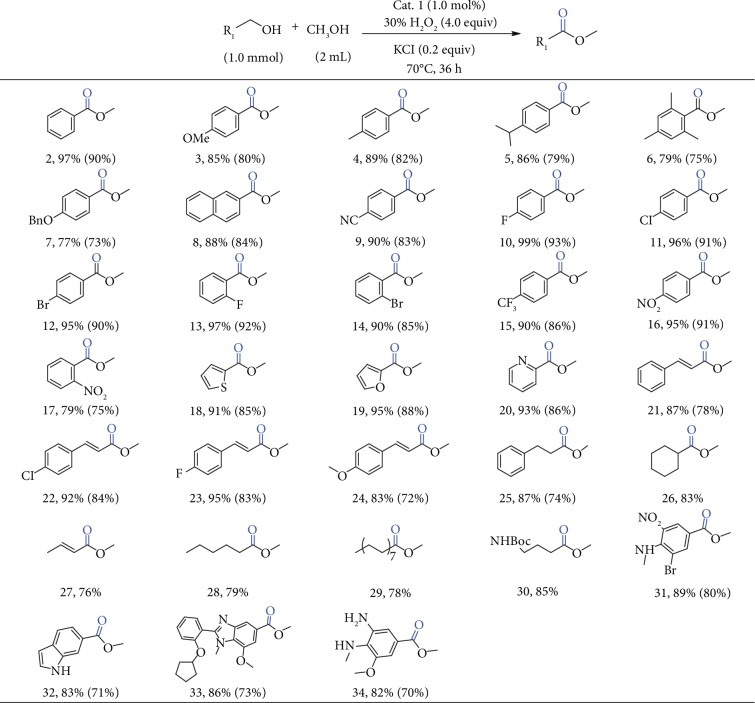
Oxidative esterification of different benzylic or aliphatic alcohols with methanol. Reaction condition: cat. 1 (1.0 mol%), aromatic alcohol (1.0 mmol), 30% H_2_O_2_ (4.0 equiv), CH_3_OH (2 mL), KCl (0.2 equiv) at 70°C for 36 h. Yields were determined using GC-Ms and column chromatography.

Once the optimal catalytic parameters were determined, the substrate scope was extended to both the benzylic and aliphatic alcohols along with the methanol partner (Figure 2). To our delight, a variety of benzylic alcohol derivatives can be converted to the targeted products in both remarkably high yields and excellent selectivity.

Aromatic alcohols containing electron-donating groups, such as *p*-methoxybenzyl alcohol, *p*-methylbenzyl alcohol, *p*-isopropylbenzyl alcohol, *p*-benzyloxybenzyl alcohol, 2-naphthalene methanol, and 2,4,6-trimethylbenzyl alcohol, all underwent completely oxidative esterification with excellent selectivity to provide the corresponding esters (compounds 2-9). Halogen-substituted aromatic alcohols were also amenable to the reaction conditions, giving the desired products in excellent yields without regard to the position of the substituents (compounds 10-14). Strongly electron-withdrawing groups, such as -CF_3_ and -NO_2_, were also tolerated under the standard conditions (compounds 15-17), but where the -NO_2_ group locates on the phenyl ring resulted in totally different reactivity. For example, benzylic alcohol substituted with the -NO_2_ group at the ortho position of the aromatic ring afforded the related ester in 79% yield. Heteroaromatic alcohols bearing sulfur, oxygen, and nitrogen atoms can be facilely converted to the corresponding products in very high yields (compounds 18–20). Even unsaturated aromatic alcohols, such as cinnamyl alcohol, gave the targeted ester in excellent yields as well (compounds 21-24). It is worth noted that reactivities of aliphatic alcohols are not comparable with benzylic alcohols, but they can also undergo aerobic oxidative esterification and give the ester products in moderate to good yields (compounds 25-30). We also studied the preparation of active drug molecules *via* oxidation of alcohols, and all the substrates tested gave the corresponding drug molecules (compounds 31-34) in good yields of 82%-89%. Moreover, the applicability of our oxidation protocol was illustrated by the scalable oxidation of 4-fluorobenzyl alcohol in good yield (90%, 10 mmol scale) ([Supplementary-material supplementary-material-1]).

**Figure 3:**
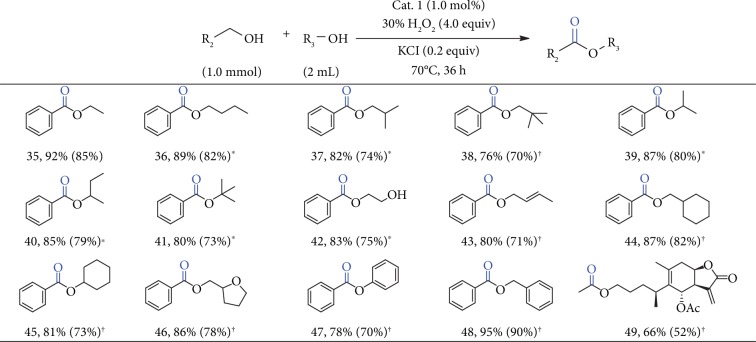
Oxidative esterification of benzylic alcohols or ethyl alcohol with different aromatic and long-chain aliphatic alcohols. Reaction conditions: cat. 1 (1.0 mol%), alcohols (R_2_) (1.0 mmol), 30% H_2_O_2_ (4.0 equiv), alcohols (R_3_) (2 mL), KCl (0.2 equiv) at 70°C for 36 h. Yields were determined using GC-Ms and column chromatography. ^∗^Nitric acid (0.7 mmol). †Alcohols (R_3_) (2.0 equiv) and nitric acid (0.7 mmol).

Upon the completion of reaction, the solid cobalt catalyst could be facilely separated *via* filtration and utilized directly for the following couplings without further purification. 1 could be recycled and used repetitively for six times with little loss in activity ([Supplementary-material supplementary-material-1]). As demonstrated by XRD and FT-IR (Figures [Supplementary-material supplementary-material-1] and [Supplementary-material supplementary-material-1]), the corresponding spectra of the recovered catalyst were almost the same as the pristine sample, confirming that 1 exhibited high stability and kept intact during the recycling process. Inspired by the success in oxidative cross-coupling of benzylic alcohols with methanol, we then further tested this promising synthetic strategy for long-chain aliphatic alcohols (Figure 3). Initially, ethyl alcohol was selected and displayed similar reactivity to methanol in this oxidative esterification reaction (compound 35). For longer chain aliphatic and branched alcohols (compounds 36-41), the desired products could be obtained from moderate to good yields. It should be noted that the pH of the reaction mixture was required to slightly decrease *via* addition of nitric acid for compounds 36 and 37.

Functionalized alcohols, such as ethylene glycol and crotyl alcohol, could all be oxidized chemoselectively to generate the corresponding esters with good yields (compounds 42 and 43). Furthermore, annular and heterocyclic alcohols also underwent facile oxidation to the corresponding esters (compounds 44-46). Even phenol could be used as a coupling partner with the desired ester being obtained in 78% yield (compound 47). Besides, benzyl alcohol can be efficiently transformed to the self-esterification product with an excellent yield up to 95% (compound 48). The active natural product, *β*-hydroxy alantolactone-containing esters group, can be easily prepared with this protocol from the corresponding alcohols (compound 49).

## 3. Discussion

**Figure 4:**
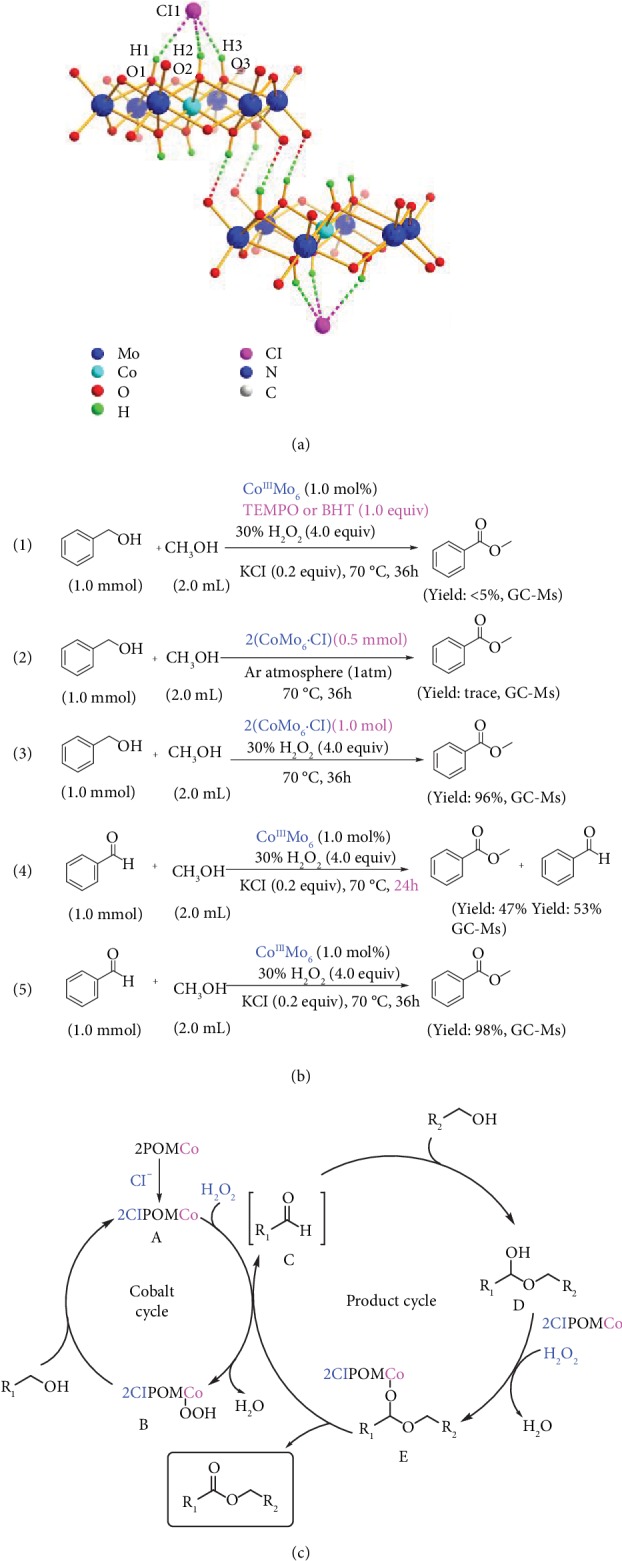
Crystal structure, control experiments, and plausible pathway of catalysis: (a) supramolecular structure of the dimeric 2(CoMo_6_∙Cl); (b) control experiments: (1) the addition of TEMPO or BHT, (2) in Ar atmosphere, (3) benzyl alcohol used as substrate under the optimum reaction condition, (4) benzaldehyde used as substrate directly for 24 h, and (5) benzaldehyde used as substrate directly for 36 h; (c) plausible pathway for the Co-catalyzed oxidative esterification reaction.

To investigate the crucial role of the chloride addictive during the catalysis, an adduct formed from chloride and cat. 1 was examined by single crystal X-ray diffraction (Figure 4(a), [Supplementary-material supplementary-material-1] and Tables [Supplementary-material supplementary-material-1] and [Supplementary-material supplementary-material-1]) and electrochemistry techniques ([Supplementary-material supplementary-material-1]). Single-crystal X-ray diffraction analysis on the adduct revealed that the chloride ion can bind to the cobalt catalyst *via* multiple hydrogen bonds to give rise to {CoMo_6_∙Cl}, which further evolves into a dimeric motif 2(CoMo_6_∙Cl) *via* the intermolecular hydrogen bonds between two CoMo_6_ (Figure 4(a)). In the supramolecular structure of dimer, the three protonated *μ*_3_-oxo ligands (O1, O2, and O3) strongly interact with chloride ion, driving the formation of three hydrogen bonds. As suggested by the short donor-acceptor distances (typically, O3∙∙∙Cl = 2.988 Å), these hydrogen bonds are comparatively strong and the accumulation of multiple hydrogen bonds is critical for overcoming electrostatic repulsion between the anionic {CoMo_6_} catalyst and chloride ion. The anion binding effect on the redox properties of the POM catalyst has been evaluated using electrochemistry techniques. Due to the strong electronic interaction between the anionic cobalt catalyst and chloride ion, the halide ion binding event can be used to tune the electrochemical properties of the cobalt catalyst. Upon addition of KCl, the characteristic redox peak of 1 shifted gradually towards a more positive potential, indicating that the 2(CoMo_6_∙Cl) complex is easier to be reduced ([Supplementary-material supplementary-material-1]). Thus, it can be seen from the CV studies that the hydrogen bonds between CoMo_6_ and chloride ion significantly alter the electrochemical properties of the whole catalyst system, which may facilitate the selective oxidation of alcohol.

**Figure 5:**
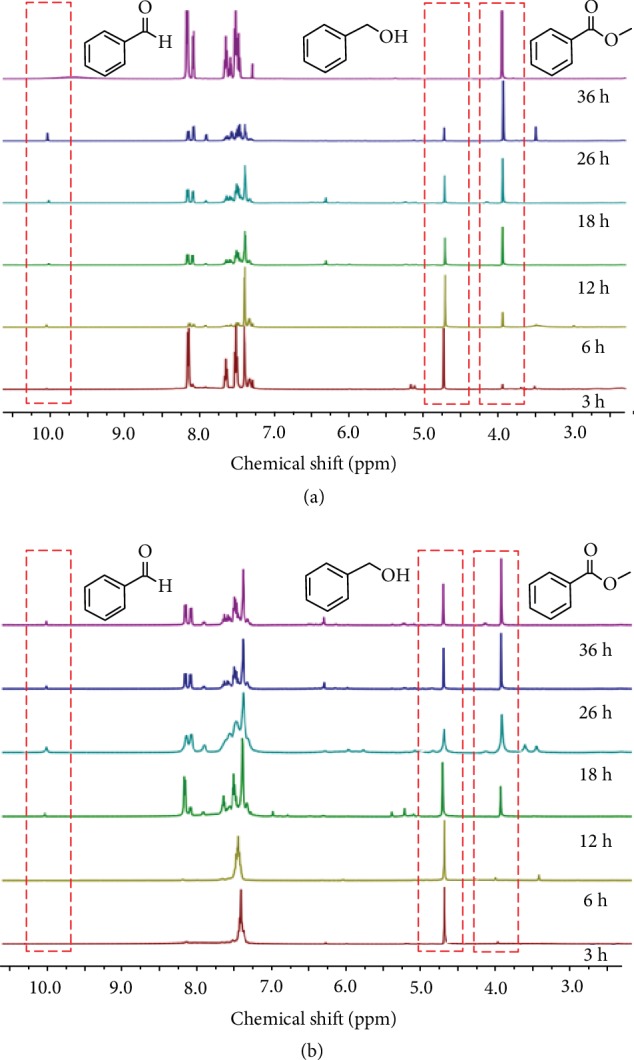
^1^H NMR spectra of the synergistic effect of the cobalt catalyst and/or KCl: (a) oxidation of benzyl alcohol and methanol with KCl; (b) oxidation of benzyl alcohol and methanol in the absence of KCl.

To gain insight into the mechanism, some control experiments were conducted. The conversion of benzyl alcohol dramatically decreased upon adding representative radical scavengers such as butylated hydroxytoluene [2,6-ditert-butyl-4-methylphenol (BHT)] or 2,2,6,6-tetramethylpiperidine-1-oxyl (TEMPO) into the reaction mixture. The obviously suppressed conversion implied that the oxidative esterification in current catalytic system proceeded based on a radical mechanism (Figure 4(b), (1)). When 2(CoMo_6_∙Cl) was employed stoichiometrically under an inert atmosphere, none of the ester product was detected upon mixing with benzyl alcohol, indicating that 2(CoMo_6_∙Cl) was not an activated oxidant (Figure 4(b), (2)). Conversely, addition of H_2_O_2_ to the stoichiometric reaction gave methyl benzoate in 96% yield (Figure 4(b), (3)), which revealed that 2(CoMo_6_∙Cl) was oxidized by H_2_O_2_ and generated a new reactive species. If the reaction was stopped after 24 h, the oxidation product methyl benzoate and benzaldehyde were both detected in 47% and 53% yields, respectively (Figure 4(b), (4)), suggesting that benzaldehyde is the key intermediate product that further undergoes esterification with methanol to afford methyl benzoate. This was further supported by the fact that methyl benzoate can be obtained in 98% yield in prolonged time exclusively using benzaldehyde directly as substrate (Figure 4(b), (5)). Since benzyl acid was not observed *via* GC-Ms and NMR, this further precluded the possible route of generating methyl benzoate involving acid and methanol. Because benzaldehyde is not further oxidized in the catalytic system, a second-step oxidation is therefore required to oxidize hemiacetal, intermediate derived from benzaldehyde and methanol, to final ester product.

Based on the experimental results and previous investigations [23, 25, 27], a plausible mechanism that underlies the catalysis cycle is depicted in Figure 4(c). Firstly, the chloride ion reacts with the cobalt catalyst to form the dimer A (2(CoMo_6_∙Cl)) *via* multiple hydrogen bonds. In the presence of H_2_O_2_, A is oxidized to active complex B which promotes the selective oxidation of alcohol to aldehyde intermediate C and regenerates A. In the presence of an alcohol partner, aldehyde C then further reacts with it to give hemiacetal intermediate D, which thereafter experiences a second cobalt-catalyzed oxidation to generate the ester product as well as E. In the end, E is reoxidized back to B by H_2_O_2_.

Figure 5 shows the ^1^H NMR studies that were performed to follow the progress of the oxidative coupling of benzyl alcohol and methanol with and without KCl as an additive. To our delight, using KCl as an additive, the production of methyl benzoate (3.89 ppm) was detected after 3 h, and benzaldehyde as the only intermediate product was also observed (10.04 ppm).

The characteristic peaks corresponding to benzyl alcohol and benzaldehyde then gradually decreased and almost completely disappeared when the reaction time was prolonged to 36 h (Figure 5(a)). In contrast, in the absence of KCl as an additive, only a very small amount of methyl benzoate and none of the benzaldehyde were observed over 3 h reaction time. After 36 h, a large amount of benzaldehyde and benzyl alcohol was still present (Figure 5(b)). As an attempt to prove the reactivity of aldehyde, KCl was then added to promote the reaction of benzaldehyde and methanol and the intermediate hemiacetal was thus generated and detected ([Supplementary-material supplementary-material-1]). These results indicate that KCl and the cobalt catalyst 1 work synergistically for the oxidative esterification of alcohols to esters.

In summary, we described here that the pure inorganic ligand-supported cobalt catalyst 1 can promote the direct oxidative esterification of a variety of alcohols using H_2_O_2_ as green oxidant. Both oxidative cross-esterification and homocoupling of benzyl alcohols with diverse aliphatic alcohols proceed quite smoothly under mild conditions, affording the corresponding ester products very efficiently. Additionally, several drug molecules and natural products have been prepared in high yields. In the inorganic cobalt-catalyzed reaction system, the chloride ion and the {CoMo_6_} synergistically promote the reaction by forming the dimeric structure of 2(CoMo_6_∙Cl) via hydrogen bonding, which is critical in modulating the reactivity and activating the substrates. The cobalt catalyst is stable and recyclable and can be easily prepared by a simple one-step synthesis from simple inorganic metal salts. Furthermore, the catalysis is achieved highly efficiently in the absence of any organic ligands. We envision that this simple, effective, and environmentally benign methodology can be potentially used for various synthetic procedures.

## 4. Materials and Methods

All reagents were purchased from Adamas-beta and Sigma-Aldrich, which were as received. ^1^H and ^13^C Nuclear Magnetic Resonance (NMR) spectra were recorded on a Bruker AVANCE III 500 MHz (500 MHz for proton, 125 MHz for carbon) spectrometer with tetramethylsilane as the internal reference using CDCl_3_ or DMSO-d_6_ as solvent in all cases, and chemical shifts were reported in parts per million (ppm, *δ*). FT-IR spectra were recorded on a Thermo-Fisher Nicolet 6700. XRD were explored on D/max 2200PC of Japan. Single-crystal X-ray diffraction analysis was performed on a Rigaku SuperNova diffractometer. GC analyses were performed on Shimadzu GC-2014 with a flame ionization detector equipped with a Rtx-1 capillary column (internal diameter = 0.25 mm, length = 30 m) or a Stabil wax capillary column (internal diameter = 0.25 mm, length = 30 m). GC-Ms spectra were recorded on Shimadzu GCMS-QP2010 with RTX-5MS capillary column. Column chromatography was performed using 300-400 mesh silica gel.

### 4.1. Preparation of Inorganic Ligand-Supported Cobalt Catalyst

(NH_4_)_3_[CoMo_6_O_18_(OH)_6_] was prepared according to literature methods with suitable modification:(NH_4_)_6_Mo_7_O_24_·4H_2_O (30.9 g) was dissolved in water (260 mL) and then heated to 100°C [39, 40]. A mixed aqueous solution (30 mL) of Co(NO_3_)_2_·6H_2_O (4.2 g) was added into the boiling aqueous solution of heptamolybdate. After being stirred for approximately 1 h, the hot solution was cooled and filtered to remove any insoluble substances. Subsequently, excess 30% aqueous H_2_O_2_ was added to the solution and placed at room temperature. The product was deposited from the filtrate and collected after a few days. IR: 3192 (*ν*asNH, m), 1636 (*δ*OH m), 1400 (*δ*NH, s), 941 (*ν* Mo = O, vs), 893 (*ν* Mo = O, vs), 655 (*ν* Mo-O-Mo, vs), and 583 (*ν*M-O-Mo, w) cm^−1^.

### 4.2. Gram-Scale Reactions

In a round bottomed flask, the cat. 1 (1.0 mol%, 0.012 g) and KCl (0.2 equiv, 14.9 mg) were added followed by the addition of 10 mL methanol. The corresponding 4-fluorophenyl methanol (10 mmol, 1.26 g) and 30% H_2_O_2_ (4.0 equiv, 0.45 g) were added. Then, reaction is stirred at 70°C for 36 h. After completion of the reaction, the mixture is cooled down to room temperature, diluted with ethyl acetate, and the catalyst was filtered off. The solvent from the filtrate containing reaction products was evaporated, and the reaction product was treated with water. The mixture was then extracted with ethyl acetate, the combined organic layers were dried over MgSO_4_, and the solvent was removed in vacuo. Finally, the corresponding methyl benzoate was purified by column chromatography (silica; petroleum ether-ethyl acetate mixture).

### 4.3. Procedure for Aerobic Oxidation of Alcohols and Recycling of Catalyst

Typically, (NH_4_)_3_[CoMo_6_O_18_(OH)_6_] (12.0 mg, 1.0 mol%) and KCl (14.9 mg, 0.2 mmol) were placed in a Schlenk tube. Aromatic alcohol (1.0 mmol), 30% H_2_O_2_ (0.45 g, 4.0 mmol), and methanol (2 mL) were sequentially added to the reaction tube. The reaction mixture was stirred at 70°C for 36 h. The reaction yield was determined via gas chromatography-mass spectrometry (GC-Ms). The resulting mixture was quenched with water. The suspension was extracted with ethyl acetate (3 × 5 mL) and the organic layers were combined, dried over sodium sulfate, and concentrated. The pure product was obtained by flash column chromatography on silica gel (silica; petroleum ether-ethyl acetate mixture).

## Figures and Tables

**Table 1:** Influence of the reaction parameters on the oxidative esterification of benzylic alcohol with methanol.
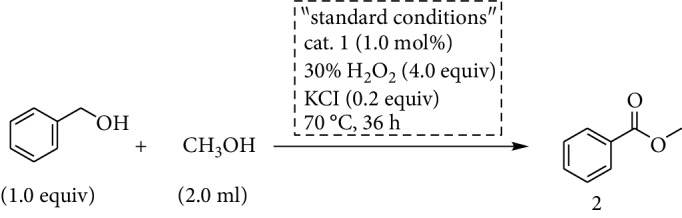

Entry	Variation from the standard conditions	Yield (%)^∗^
1	None	97
2	Without cat. 1	—
3	N_2_ (1.0 bar) instead of H_2_O_2_ (4.0 equiv)	<5
4	O_2_ (1.0 bar) instead of H_2_O_2_ (4.0 equiv)	20
5	Co(NO_3_)_2·_6H_2_O instead of cat. 1	Trace
6	NH_4_Mo_7_O_24·_4H_2_O instead of cat. 1	Trace
7	Co(NO_3_)_2·_6H_2_O+ NH_4_Mo_7_O_24·_4H_2_O	<10
8	Without KCl	37

^∗^Yields were determined by GC-Ms analysis of the crude reaction mixtures.

## Data Availability

The authors declare that all data supporting the findings of this study are available within in the article and Supplementary Information files and are also available from the corresponding authors upon reasonable request.

## Supplementary Materials

Supplementary Materials Supplementary Text Figure S1: the process of catalyst preparation. Figure S2: the FTIR spectra of (NH_4_)_3_[CoMo_6_O_18_(OH)_6_]. Figure S3: the XRD spectra of (NH_4_)_3_[CoMo_6_O_18_(OH)_6_]. Figure S4: cluster structure of the monomer (a) and dimer (b). Figure S5: cyclic voltammogram experiments. Figure S6. Gram scale reactions. Figure S7: recycling of cobalt catalyst for oxidative esterification of benzyl alcohol and methanol. Figure S8: the FTIR spectra of the catalyst before and after the sixth reaction. Figure S9: the XRD spectra of the catalyst before and after the sixth reaction. Figure S10: ^1^H NMR spectral study of the oxidation of benzaldehyde and methanol in the presence of KCl with time evolving from 3 h to 24 h. Figures S11-S54: ^1^H NMR (top) and 13C NMR (bottom) of 3-50 (except of 28-31 which yields were determined by GC-Ms, all of them used CDCl_3_ as the solvent). Table S1: the effect of additive on the oxidative esterification of benzylic alcohol with methanol. Table S2: the effect of positive ion of additive on the oxidative esterification of benzylic alcohol with methanol. Table S3: impact of reaction parameters on the oxidative esterification of benzylic alcohol with methanol. Table S4: investigation the influence of the metal center of catalyst 1 on the oxidative esterification of benzylic alcohol with methanol. Table S5: crystal data and structure refinement for CoMo_6_/Cl. Table S6: hydrogen bonds in the dimeric cluster.
