# Supplementary material for: Additive-Mediated Selective Oxidation of Alcohols to Esters via Synergistic Effect Using Single Cation Cobalt Catalyst Stabilized with Inorganic Ligand
Source: Research (Wash D C). 2020 Jan 23;2020:3875920. doi: 10.34133/2020/3875920 (PMC6998037; doi:10.34133/2020/3875920)
Supplement: Supplementary Materials — Supplementary Text Figure S1: the process of catalyst preparation. Figure S2: the FTIR spectra of (NH4)3[CoMo6O18(OH)6]. Figure S3: the XRD spectra of (NH4)3[CoMo6O18(OH)6]. Figure S4: cluster structure of the monomer (a) and dimer (b). Figure S5: cyclic voltammogram experiments. Figure S6. Gram scale reactions. Figure S7: recycling of cobalt catalyst for oxidative esterification of benzyl alcohol and methanol. Figure S8: the FTIR spectra of the catalyst before and after the sixth reaction. Figure S9: the XRD spectra of the catalyst before and after the sixth reaction. Figure S10: 1H NMR spectral study of the oxidation of benzaldehyde and methanol in the presence of KCl with time evolving from 3 h to 24 h. Figures S11-S54: 1H NMR (top) and 13C NMR (bottom) of 3-50 (except of 28-31 which yields were determined by GC-Ms, all of them used CDCl3 as the solvent). Table S1: the effect of additive on the oxidative esterification of benzylic alcohol with methanol. Table S2: the effect of positive ion of additive on the oxidative esterification of benzylic alcohol with methanol. Table S3: impact of reaction parameters on the oxidative esterification of benzylic alcohol with methanol. Table S4: investigation the influence of the metal center of catalyst 1 on the oxidative esterification of benzylic alcohol with methanol. Table S5: crystal data and structure refinement for CoMo6/Cl. Table S6: hydrogen bonds in the dimeric cluster. [file 3875920.f1.doc]

**Supplementary Materials for**

**Additive-Mediated Selective Oxidation of Alcohols to Esters via Synergistic Effect Using Single Cation Cobalt Catalyst Stabilized with Inorganic Ligand**

**Jingjing Wang^1^, Han Yu^1,2,3^,****^*^, Zheyu Wei^1^, Qi Li^2^, Weimin Xuan^4,*^, and Yongge Wei^2,3,*^**

*Contributed author. Email:  [Weimin Xuan; weiminxuan@dhu.edu.cn; yonggewei@mail.tsinghua.edu.cn](mailto:hansheng654321@sina.com; yonggewei@mail.tsinghua.edu.cn); [hanyu0220@tsinghua.edu](mailto:hanyu0220@tsinghua.edu).cn

**This PDF file includes:**

Supplementary Text

Figure S1. The process of catalyst preparation.

Figure S2. The FTIR spectra of (NH_4_)_3_[CoMo_6_O_18_(OH)_6_].

Figure S3. The XRD spectra of (NH_4_)_3_[CoMo_6_O_18_(OH)_6_].

Figure S4. Cluster structure of the monomer (a) and dimer (b).

Figure S5. Cyclic voltammogram experiments.

Figure S6. Gram scale reactions.

Figure S7. Recycling of cobalt catalyst for oxidative esterification of benzyl alcohol and methanol.

Figure S8. The FTIR spectra of the catalyst before and after the sixth reaction.

Figure S9. The XRD spectra of the catalyst before and after the sixth reaction.

Figure S10. ^1^H NMR spectral study of the oxidation of benzaldehyde and methanol in the presence of KCl with time evolving from 3h to 24 h.

Figures S11-S54. ^1^H NMR (top) and ^13^C NMR (bottom) of 3-50 (except of 28-31 which yields were determined by GC-Ms, all of them used CDCl_3_ as the solvent).

Table S1. Crystal data and structure refinement for CoMo_6_/Cl.

Table S2. Hydrogen bonds in the dimeric cluster.

Table S3. The effect of additive on the oxidative esterification of benzylic alcohol with methanol.

Table S4. Impact of reaction parameters on the oxidative esterification of benzylic alcohol with methanol.

Table S5. The effect of [positive](javascript:;) [ion](javascript:;) of additive on the oxidative esterification of benzylic alcohol with methanol.

**Supplementary Text**

**Cyclic voltammogram.** Cyclic voltammograms obtained at the glassy carbon electrode and a 1.0 mM acetonitrile solution of the CoMo_6_ in the presence of increasing amounts of KCl at sweep rates of 100 mV s^-1^.

**Preparation of the crystals of 2(CoMo_6_∙Cl).** Single crystals of chloride ion/ POM complex 2(CoMo_6_∙Cl) were obtained by evaporation of an aqueous solution of [NH_4_]_3_[CoMo_6_O_18_(OH)_6_] (10.0 mmol) with stoichiometric ratio of [(C_4_H_9_)_4_N]Br (10.0 mmol) and excess KCl (200.0 mmol) added.

**X-ray Crystallography.** Single-crystal X-ray diffraction analysis was performed on a Rigaku Super Nova diffractometer at 50 kV and 20 mA, using graphite monochromatized Mo Kα radiation (λ = 0.71073 Å) at 100 K. Data collection, data reduction, cell refinement, and experimental absorption correction were performed with the software package of CrysAlisPro 1.171.39.46 (Rigaku Oxford Diffraction, 2018). Structures were solved by direct methods and refined against F2 by full matrix least squares. All non-hydrogen atoms were refined anisotropically. Hydrogen atoms were generated geometrically. All calculations were performed using the SHELXS-974 in Olex25 program package. (CCDC: 1882681) These data can be obtained free of charge from The Cambridge Crystallographic Data Centre via www.ccdc.cam.ac.uk/data_request/cif.

**NMR data of products.**

**Methyl benzoate:** GC: t_R_=6.74 min; Colorless liquid; Eluent (petroleum ether: ethyl acetate, 20: 1)

^1^H NMR (501 MHz, CDCl_3_) δ 8.06 (d, J = 7.4 Hz, 2H), 7.57 (t, J = 7.4 Hz, 1H), 7.46 (t, J = 7.7 Hz, 2H), 3.94 (s, 3H).

^13^C NMR (126 MHz, CDCl_3_) δ 167.14 (s), 132.94 (s), 130.16 (s), 129.59 (s), 128.38 (s), 52.13 (s).

**Methyl anisate:** GC: t_R_=11.49 min; White powder; Eluent (petroleum ether: ethyl acetate, 30: 1)

^1^H NMR (501 MHz, CDCl_3_) δ 7.96 (d, J = 8.9 Hz, 2H), 6.87 (d, J = 8.9 Hz, 2H), 3.82 (d, J = 24.3 Hz, 6H).

^13^C NMR (126 MHz, CDCl_3_) δ 166.98 – 166.82 (m), 163.41 (s), 131.51 – 131.35 (m), 122.51 (s), 113.42 (s), 55.32 (s), 51.67 – 51.51 (m).

**Methyl 4-methylbenzoate:** GC: t_R_=8.58 min; White crystalline solid; Eluent (petroleum ether: ethyl acetate, 30: 1)

^1^H NMR (501 MHz, CDCl_3_) δ 7.93 (d, J = 8.1 Hz, 2H), 7.22 (d, J = 8.0 Hz, 2H), 3.89 (s, 3H), 2.39 (s, 3H).

^13^C NMR (126 MHz, CDCl_3_) δ 167.15 (s), 143.54 (s), 129.59 (s), 129.07 (s), 127.41 (s), 51.90 (s), 21.60 (s).

**Methyl 4-propan-2-ylbenzoate:** GC: t_R_=11.50 min; Light yellow liquid; Eluent (petroleum ether: ethyl acetate, 30: 1)

^1^H NMR (501 MHz, CDCl_3_) δ 7.99 (d, J = 8.2 Hz, 2H), 7.31 (d, J = 8.2 Hz, 2H), 3.92 (s, 3H), 1.28 (d, J = 6.9 Hz, 6H).

^13^C NMR (126 MHz, CDCl_3_) δ 167.19 (s), 154.26 (s), 129.75 (s), 127.78 (s), 126.48 (s), 51.95 (s), 34.26 (s), 23.72 (s).

**Methyl 2,4,6-trimethylbenzoate:** GC: t_R_=10.83 min; Light yellow liquid; Eluent (petroleum ether: ethyl acetate, 20: 1)

^1^H NMR (501 MHz, CDCl_3_) δ 6.87 (s, 2H), 3.91 (s, 3H), 2.34 (s, 6H), 2.30 (s, 3H).

^13^C NMR (126 MHz, CDCl_3_) δ 170.64 (s), 143.84 (s), 141.56 (s), 135.18 (s), 128.39 (s), 51.86 (s), 21.48 (s), 20.51 (s).

**Methyl 4-n-butoxybenzoate:** GC: t_R_=18.38 min; Colorless liquid; Eluent (petroleum ether: ethyl acetate, 30: 1)

^1^H NMR (501 MHz, CDCl_3_) δ 7.95 (d, J = 8.8 Hz, 2H), 6.87 (d, J = 8.8 Hz, 2H), 3.96 (t, J = 6.5 Hz, 2H), 3.84 (s, 3H), 1.75 (d, J = 7.4 Hz, 2H), 1.48 – 1.45 (m, 2H), 0.96 (d, J = 3.4 Hz, 3H).

^13^C NMR (126 MHz, CDCl_3_) δ 164.24 (s), 162.95 (s), 131.52 (s), 122.26 (s), 114.01 (s), 68.05 (s), 51.73 (s), 31.13 (s), 19.15 (s), 13.78 (s).

**Methyl 2-naphthoate:** GC: t_R_=18.25 min; White powder; Eluent (petroleum ether: ethyl acetate, 20: 1)

^1^H NMR (501 MHz, CDCl_3_) δ 8.64 (s, 1H), 8.09 (d, J = 8.5 Hz, 1H), 7.97 (d, J = 8.0 Hz, 1H), 7.90 (d, J = 8.9 Hz, 2H), 7.59 (dt, J = 14.8, 7.1 Hz, 2H), 4.01 (s, 3H).

^13^C NMR (126 MHz, CDCl_3_) δ 167.30 (s), 135.53 (s), 132.51 (s), 131.10 (s), 129.38 (s), 128.23 (d, J = 10.7 Hz), 127.79 (s), 127.40 (s), 126.67 (s), 125.25 (s), 52.28 (s).

**4-cyanobenzoic acid methyl ester:** GC: t_R_=11.14 min; White crystal; Eluent (petroleum ether: ethyl acetate, 30: 1)

^1^H NMR (501 MHz, CDCl_3_) δ 8.15 (d, J = 8.3 Hz, 2H), 7.76 (d, J = 8.3 Hz, 2H), 3.97 (s, 3H).

^13^C NMR (126 MHz, CDCl_3_) δ 165.46 (s), 133.92 (s), 132.26 (s), 130.11 (s), 118.00 (s), 116.38 (s), 52.77 (s).

**Methyl 4-fluorobenzoate:** GC: t_R_=6.43 min; Colorless liquid; Eluent (petroleum ether: ethyl acetate, 20: 1)

^1^H NMR (501 MHz, CDCl_3_) δ 8.22 – 7.86 (m, 2H), 7.12 (t, J = 7.9 Hz, 2H), 3.92 (s, 3H).

^13^C NMR (126 MHz, CDCl_3_) δ 166.77 (s), 166.11 (s), 164.75 (s), 132.11 (d, J = 9.3 Hz), 126.38 (s), 115.39 (s), 51.99 (s).

**4-chlorobenzoic acid methyl ester:** GC: t_R_=9.23 min; Light yellow solid; Eluent (petroleum ether: ethyl acetate, 20: 1)

^1^H NMR (501 MHz, CDCl_3_) δ 7.93 (d, J = 8.6 Hz, 2H), 7.37 (d, J = 8.6 Hz, 2H), 3.88 (s, 3H).

^13^C NMR (126 MHz, CDCl_3_) δ 166.11 (s), 139.30 (s), 130.93 (s), 128.60 (d, J = 13.6 Hz), 52.21 (s).

**Methyl 4-bromobenzoate:** GC: t_R_=10.74 min; White solid; Eluent (petroleum ether: ethyl acetate, 20: 1)

^1^H NMR (501 MHz, CDCl_3_) δ 7.87 (d, J = 8.3 Hz, 2H), 7.55 (d, J = 8.3 Hz, 2H), 3.90 (s, 3H).

^13^C NMR (126 MHz, CDCl_3_) δ 166.29 (s), 131.69 (s), 131.11 (s), 129.01 (s), 128.03 (s), 52.29 (s).

**Methyl 2-fluorobenzoate:** GC: t_R_=7.04 min; colourless liquid; Eluent (petroleum ether: ethyl acetate, 30: 1)

^1^H NMR (501 MHz, CDCl_3_) δ 7.95 (t, J = 7.1 Hz, 1H), 7.55 – 7.49 (m, 1H), 7.21 (t, J = 7.6 Hz, 1H), 7.16 – 7.12 (m, 1H), 3.94 (s, 3H).

^13^C NMR (126 MHz, CDCl_3_) δ 164.93 (d, J = 3.9 Hz), 162.95 (s), 160.89 (s), 134.52 (d, J = 9.0 Hz), 132.14 (s), 123.98 (d, J = 3.9 Hz), 118.59 (d, J = 9.6 Hz), 117.07 (s), 116.89 (s), 52.35 (s).

**Methyl 2-bromobenzoate:** GC: t_R_=10.98 min; Light yellow liquid; Eluent (petroleum ether: ethyl acetate, 30: 1)

^1^H NMR (501 MHz, CDCl_3_) δ 7.79 (dd, J = 13.6, 6.4 Hz, 1H), 7.66 (dd, J = 15.4, 7.7 Hz, 1H), 7.36 – 7.21 (m, 2H), 3.93 (d, J = 14.3 Hz, 3H).

^13^C NMR (126 MHz, CDCl_3_) δ 166.60 (d, J = 2.9 Hz), 134.33 (s), 132.59 (s), 131.32 (s), 128.38 (s), 127.18 (s), 121.66 (s), 52.46 (s).

**Methyl 4-(trifluoromethyl)benzoate:** GC: t_R_=6.41min; Colorless liquid; Eluent (petroleum ether: ethyl acetate, 30: 1)

^1^H NMR (501 MHz, CDCl_3_) δ 8.17 (d, J = 7.9 Hz, 2H), 7.72 (d, J = 7.9 Hz, 2H), 3.98 (s, 3H).

^13^C NMR (126 MHz, CDCl_3_) δ 165.84 (s), 134.60 (s), 133.40 (s), 129.97 (s), 125.34 (s), 122.55 (s), 52.56 (s).

**Methyl 4-nitrobenzoate:** GC: t_R_=13.84 min; Yellow solid; Eluent (petroleum ether: ethyl acetate, 30: 1)

^1^H NMR (501 MHz, CDCl_3_) δ 8.29 (d, J = 8.8 Hz, 2H), 8.21 (d, J = 8.8 Hz, 2H), 3.98 (s, 3H).

^13^C NMR (126 MHz, CDCl_3_) δ 165.18 (s), 150.72 (s), 135.41 (s), 130.78 (s), 123.38 (s), 52.73 (s).

**2-Nitrobenzoic acid methyl ester:** GC: t_R_=12.97 min; Yellow solid; Eluent (petroleum ether: ethyl acetate, 30: 1)

^1^H NMR (501 MHz, CDCl_3_) δ 7.64 (d, J = 16.0 Hz, 1H), 7.45 (s, 1H), 7.36 (d, J = 6.6 Hz, 1H), 6.42 (d, J = 16.0 Hz, 1H), 3.82 (s, 3H).

^13^C NMR (126 MHz, CDCl_3_) δ 167.18 (s), 143.42 (s), 136.19 (s), 132.87 (s), 129.21 (d, J = 6.9 Hz), 118.38 (s), 51.81 (s).

**Methyl thiophene-2-carboxylate:** GC: t_R_=6.98 min; Light yellow liquid; Eluent (petroleum ether: ethyl acetate, 20: 1)

^1^H NMR (501 MHz, CDCl_3_) δ 7.80 (d, J = 3.6 Hz, 1H), 7.55 (d, J = 4.9 Hz, 1H), 7.19 – 7.02 (m, 1H), 3.89 (s, 3H).

^13^C NMR (126 MHz, CDCl_3_) δ 162.71 (s), 133.52 (d, J = 8.2 Hz), 132.39 (s), 127.77 (s), 52.16 (s).

**Methyl 2-furoate:** GC: t_R_=4.85 min; Light yellow liquid; Eluent (petroleum ether: ethyl acetate, 20: 1)

^1^H NMR (501 MHz, CDCl_3_) δ 7.79 – 7.43 (m, 1H), 7.29 – 7.01 (m, 1H), 6.61 – 6.46 (m, 1H), 3.91 (s, 3H).

^13^C NMR (126 MHz, CDCl_3_) δ 159.15 (s), 146.29 (s), 144.65 (s), 142.81 (s), 117.95 (s), 111.85 (s), 51.90 (s).

**Methyl picolinate:** GC: t_R_=8.15 min; Colorless liquid; Eluent (ethyl acetate)

^1^H NMR (501 MHz, CDCl_3_) δ 8.77 (d, J = 4.4 Hz, 1H), 8.16 (d, J = 7.8 Hz, 1H), 7.87 (t, J = 7.2 Hz, 1H), 7.51 (dd, J = 6.3, 5.9 Hz, 1H), 4.03 (s, 3H).

^13^C NMR (126 MHz, CDCl_3_) δ 165.71 (s), 149.81 (s), 147.78 (s), 137.11 (s), 127.00 (s), 125.17 (s), 52.91 (s).

**Methyl cinnamate:** GC: t_R_=11.70 min; White crystal; Eluent (petroleum ether: ethyl acetate, 10: 1)

^1^H NMR (501 MHz, CDCl_3_) δ 7.72 (d, J = 16.0 Hz, 1H), 7.54 (d, J = 3.5 Hz, 2H), 7.43 – 7.36 (m, 3H), 6.47 (d, J = 16.0 Hz, 1H), 3.82 (s, 3H).

^13^C NMR (126 MHz, CDCl_3_) δ 167.47 (s), 144.91 (s), 134.37 (s), 130.34 (s), 128.92 (s), 128.11 (s), 117.79 (s), 51.74 (s).

**Methyl 4-chlorocinnamate:** GC: t_R_=13.98 min; Light yellow crystals; Eluent (petroleum ether: ethyl acetate, 20: 1)

^1^H NMR (501 MHz, CDCl_3_) δ 7.64 (d, J = 16.0 Hz, 1H), 7.45 (d, J = 8.5 Hz, 2H), 7.36 (d, J = 8.5 Hz, 2H), 6.42 (d, J = 16.0 Hz, 1H), 3.82 (s, 3H).

^13^C NMR (126 MHz, CDCl_3_) δ 167.18 (s), 143.42 (s), 136.21 (s), 132.87 (s), 129.21 (d, J = 6.9 Hz), 118.38 (s), 51.79 (s).

**Methyl 4-** **fluorocinnamate:** GC: t_R_=11.59 min; Light yellow liquid; Eluent (petroleum ether: ethyl acetate, 20: 1)

^1^H NMR (501 MHz, CDCl_3_) δ 7.66 (d, J = 16.0 Hz, 1H), 7.52 (dd, J = 8.6, 5.4 Hz, 2H), 7.08 (t, J = 8.6 Hz, 2H), 6.37 (d, J = 16.0 Hz, 1H), 3.81 (s, 3H).

^13^C NMR (126 MHz, CDCl_3_) δ 167.30 (s), 164.86 (s), 162.91 (s), 143.56 (s), 130.63 (d, J = 3.3 Hz), 129.94 (d, J = 8.5 Hz), 117.54 (s), 116.13 (s), 115.95 (s), 51.81 (s).

**(3-Methoxyphenyl)methyl prop-2-enoate:** GC: t_R_=18.77 min; Light yellow crystal; Eluent (petroleum ether: ethyl acetate, 20: 1)

^1^H NMR (501 MHz, CDCl_3_) δ 7.63 (d, J = 16.0 Hz, 1H), 7.45 (d, J = 8.5 Hz, 2H), 6.88 (d, J = 8.4 Hz, 2H), 6.29 (d, J = 15.9 Hz, 1H), 3.79 (d, J = 16.9 Hz, 6H).

^13^C NMR (126 MHz, CDCl_3_) δ 167.73 (s), 161.40 (s), 144.51 (s), 129.71 (s), 127.06 (s), 115.22 (s), 114.31 (s), 55.55 (s), 51.52 (s).

**3-Phenylpropionic acid methyl ester:** GC: t_R_=9.53min; White crystal; Eluent (petroleum ether: ethyl acetate, 30: 1)

^1^H NMR (501 MHz, CDCl_3_) δ 7.34 (t, J = 7.5 Hz, 2H), 7.25 (d, J = 7.6 Hz, 3H), 3.38 (s, 3H), 2.78 – 2.61 (m, 2H), 1.98 (dd, J = 15.8, 5.9 Hz, 2H).

^13^C NMR (126 MHz, CDCl_3_) δ 173.34 (s), 141.67 (s), 128.65 – 128.22 (m), 126.31 (s), 125.93 (s), 52.73 (s), 34.14 (s), 30.92 (s).

**Cyclohexanecarboxylic acid methyl ester:** GC: t_R_=6.18 min; Light yellow liquid; Eluent (petroleum ether: ethyl acetate, 30: 1)

^1^H NMR (501 MHz, CDCl_3_) δ 3.67 (s, 3H), 2.39 – 2.21 (m, 1H), 1.90 (d, J = 12.8 Hz, 2H), 1.80 – 1.61 (m, 3H), 1.52 – 1.39 (m, 2H), 1.35 – 1.19 (m, 3H).

^13^C NMR (126 MHz, CDCl_3_) δ 176.59 (s), 51.44 (s), 43.11 (s), 29.02 (s), 25.75 (s), 25.45 (s).

**Methyl 3-bromo-4-(methylamino)-5-nitrobenzoate:** Yellow solid; Eluent (petroleum ether: ethyl acetate, 20: 1)

^1^H NMR (501 MHz, CDCl_3_) δ 8.51 (s, 1H), 8.29 (s, 1H), 3.92 (s, 3H), 3.09 (s, 3H).

^13^C NMR (126 MHz, CDCl_3_) δ 164.41 (s), 145.39 (s), 138.55 (s), 128.20 (s), 125.65 (s), 118.74 (s), 111.45 (s), 52.45 (s), 33.83 (s).

**Methyl 1*H*-indole-6-carboxylate:** Light yellow solid; Eluent (petroleum ether: ethyl acetate, 20: 1)

^1^H NMR (501 MHz, CDCl_3_) δ 8.20 (s, 1H), 7.84 (d, J = 8.2 Hz, 1H), 7.69 (d, J = 8.3 Hz, 1H), 7.39 (s, 1H), 6.63 (s, 1H), 3.96 (s, 3H).

^13^C NMR (126 MHz, CDCl_3_) δ 168.31 (s), 135.15 (s), 131.60 (s), 127.60 (s), 123.65 (s), 120.84 (s), 120.30 (s), 113.53 (s), 102.98 (s), 51.99 (s).

**Methyl 2-(2-(cyclopentyloxy)phenyl)-7-methoxy-1-methyl-1H-benzo[d]imidazole-5-carboxylate:** Light brown solid; Eluent (petroleum ether: ethyl acetate, 20: 1)

^1^H NMR (501 MHz, CDCl_3_) δ 8.20 (s, 1H), 7.58 (d, J = 7.2 Hz, 1H), 7.49 (dd, J = 15.9, 7.8 Hz, 2H), 7.10 (t, J = 7.4 Hz, 1H), 7.03 (d, J = 8.4 Hz, 1H), 4.81 (s, 1H), 4.04 (s, 3H), 3.96 (s, 3H), 3.90 (s, 3H), 1.89 (dd, J = 12.9, 6.2 Hz, 2H), 1.81 – 1.73 (m, 2H), 1.64 – 1.51 (m, 4H).

^13^C NMR (126 MHz, CDCl_3_) δ 167.77 (s), 156.16 (s), 154.14 (s), 146.86 (s), 132.57 (s), 131.62 (s), 128.70 (s), 124.39 (s), 120.71 (s), 119.78 (s), 115.52 (s), 113.59 (s), 104.26 (s), 80.33 (s), 55.84 (s), 52.09 (s), 33.81 (s), 32.87 (s), 23.98 (s).

**Benzoic acid, 3-amino-5-methoxy-4-(methylamino)-, methyl ester:** Light yellow solid; Eluent (petroleum ether: ethyl acetate, 20: 1)

^1^H NMR (501 MHz, CDCl_3_) δ 7.12 (d, J = 0.7 Hz, 1H), 7.05 (s, 1H), 3.89 (s, 6H), 2.80 (s, 3H).

^13^C NMR (126 MHz, CDCl_3_) δ 167.16 (s), 151.84 (s), 140.08 (s), 124.86 (s), 111.20 (s), 105.81 – 105.65 (m), 102.62 (s), 56.00 (s), 51.96 (s), 33.66 (s).

**Ethyl benzoate:** GC: t_R_=7.94 min; Light yellow liquid; Eluent (petroleum ether: ethyl acetate, 10: 1)

^1^H NMR (501 MHz, CDCl_3_) δ 8.07 (d, J = 7.5 Hz, 2H), 7.56 (t, J = 7.4 Hz, 1H), 7.45 (t, J = 7.6 Hz, 2H), 4.39 (q, J = 7.1 Hz, 2H), 1.41 (t, J = 7.1 Hz, 3H).

^13^C NMR (126 MHz, CDCl_3_) δ 166.65 (s), 132.80 – 132.64 (m), 130.44 (s), 129.54 (s), 128.27 (s), 60.97 (s), 14.35 (s).

**Butyl benzoate:** GC: t_R_=11.41 min; Colorless liquid; Eluent (petroleum ether: ethyl acetate, 30: 1)

^1^H NMR (501 MHz, CDCl_3_) δ 8.07 (d, J = 7.4 Hz, 2H), 7.57 (t, J = 7.3 Hz, 1H), 7.46 (t, J = 7.7 Hz, 2H), 4.35 (t, J = 6.6 Hz, 2H), 1.82 – 1.74 (m, 2H), 1.50 (dt, J = 14.8, 7.4 Hz, 2H), 1.01 (t, J = 7.4 Hz, 3H).

^13^C NMR (126 MHz, CDCl_3_) δ 166.73 (s), 132.80 (s), 130.60 (s), 129.54 (s), 128.26 (s), 65.02 (s), 30.79 (s), 19.21 – 19.05 (m), 13.78 (s).

**Isobutyl benzoate:** GC: t_R_=10.50 min; Colorless liquid; Eluent (petroleum ether: ethyl acetate, 20: 1)

^1^H NMR (501 MHz, CDCl_3_) δ 8.08 (d, J = 7.3 Hz, 2H), 7.57 (t, J = 7.3 Hz, 1H), 7.46 (t, J = 7.7 Hz, 2H), 4.14 (d, J = 6.6 Hz, 2H), 2.11 (dp, J = 13.4, 6.7 Hz, 1H), 1.05 (d, J = 6.7 Hz, 6H).

^13^C NMR (126 MHz, CDCl_3_) δ 166.56 (s), 132.81 (s), 130.56 (s), 129.55 (s), 128.34 (s), 71.01 (s), 27.93 (s), 19.21 (s).

**2,2-Dimethylpropyl benzoate:** GC: t_R_=11.24 min; Light yellow liquid; Eluent (petroleum ether: ethyl acetate, 30: 1)

^1^H NMR (501 MHz, CDCl_3_) δ 8.09 (d, J = 7.4 Hz, 2H), 7.57 (t, J = 7.4 Hz, 1H), 7.46 (t, J = 7.6 Hz, 2H), 4.04 (s, 2H), 1.07 (s, 9H).

^13^C NMR (126 MHz, CDCl_3_) δ 166.61 (s), 132.84 (s), 130.57 (s), 129.54 (s), 128.37 (s), 74.22 (s), 31.62 (s), 26.60 (s).

**Benzoic acid isopropyl ester:** GC: t_R_=8.46 min; Colorless liquid; Eluent (petroleum ether: ethyl acetate, 20: 1)

^1^H NMR (501 MHz, CDCl_3_) δ 8.07 (d, J = 7.4 Hz, 2H), 7.56 (t, J = 7.4 Hz, 1H), 7.45 (t, J = 7.6 Hz, 2H), 5.27 (dq, J = 12.4, 6.2 Hz, 1H), 1.39 (d, J = 6.3 Hz, 6H).

^13^C NMR (126 MHz, CDCl_3_) δ 166.12 (s), 132.69 (s), 130.93 (s), 129.51 (s), 128.26 (s), 68.40 (s), 21.96 (s).

**S-butyl benzoate:** GC: t_R_=10.50 min; Colorless liquid; Eluent (petroleum ether: ethyl acetate, 20: 1)

^1^H NMR (501 MHz, CDCl_3_) δ 8.07 (d, J = 3.3 Hz, 2H), 7.66 (s, 2H), 7.47 – 7.45 (m, 2H), 5.12 (dt, J = 12.5, 6.2 Hz, 1H), 1.77 – 1.65 (m, 2H), 1.36 (d, J = 6.3 Hz, 3H), 1.05 (d, J = 6.7 Hz, 3H).

^13^C NMR (126 MHz, CDCl_3_) δ 166.28 (s), 133.66 (s), 130.18 (s), 129.53 (d, J = 4.0 Hz), 128.48 (s), 72.87 (s), 28.98 (s), 19.20 (s), 9.72 (s).

**Benzoic acid tert-butyl ester:** GC: t_R_=13.34 min; Colorless liquid; Eluent (petroleum ether: ethyl acetate, 40: 1)

^1^H NMR (501 MHz, CDCl_3_) δ 7.98 (d, J = 7.4 Hz, 2H), 7.61 (t, J = 7.4 Hz, 1H), 7.48 (t, J = 7.7 Hz, 2H), 1.44 (s, 9H).

^13^C NMR (126 MHz, CDCl_3_) δ 164.46 (s), 133.36 (s), 129.89 (s), 129.15 (s), 128.64 (s), 84.01 (s), 26.27 (s).

**2-Hydroxyethyl benzoate:** GC: t_R_=13.06 min; Light yellow liquid; Eluent (petroleum ether: ethyl acetate, 30: 1)

^1^H NMR (501 MHz, CDCl_3_) δ 8.05 (d, J = 7.4 Hz, 2H), 7.55 (t, J = 7.4 Hz, 1H), 7.42 (t, J = 7.7 Hz, 2H), 4.52 – 4.38 (m, 2H), 4.01 – 3.89 (m, 2H), 3.26 (s, 1H).

^13^C NMR (126 MHz, CDCl_3_) δ 167.07 (s), 133.21 (s), 129.99 (s), 129.76 (d, J = 16.0 Hz), 128.43 (s), 66.59 (s), 61.12 (s).

**(E)-but-2-en-1-yl benzoate:** GC: t_R_=11.80 min; Light yellow liquid; Eluent (petroleum ether: ethyl acetate, 30: 1)

^1^H NMR (501 MHz, CDCl_3_) δ 8.08 (d, J = 7.6 Hz, 2H), 7.57 (d, J = 6.5 Hz, 1H), 7.45 (t, J = 7.6 Hz, 2H), 5.90 (td, J = 13.0, 6.4 Hz, 1H), 5.76 – 5.70 (m, 1H), 4.78 (d, J = 6.4 Hz, 2H), 1.78 (d, J = 6.3 Hz, 3H).

^13^C NMR (126 MHz, CDCl_3_) δ 166.46 (s), 132.85 (s), 131.34 (s), 130.43 (s), 129.61 (s), 128.32 (s), 125.20 (s), 65.65 (s), 17.80 (s).

**Cyclohexylmethyl benzoate:** GC: t_R_=20.03 min; Light yellow liquid; Eluent (petroleum ether: ethyl acetate, 30: 1)

^1^H NMR (501 MHz, CDCl_3_) δ 8.03 (d, J = 8.2 Hz, 2H), 7.49 (t, J = 7.3 Hz, 1H), 7.39 (t, J = 7.3 Hz, 2H), 4.10 (t, J = 6.5 Hz, 2H), 1.80 (d, J = 12.9 Hz, 2H), 1.69 (dd, J = 32.4, 12.5 Hz, 4H), 1.28 – 1.12 (m, 3H), 1.03 (dd, J = 23.8, 12.0 Hz, 2H).

^13^C NMR (126 MHz, CDCl_3_) δ 166.46 (s), 132.70 (s), 130.55 (s), 129.49 (s), 128.25 (s), 69.93 (s), 37.27 (s), 29.74 (s), 26.37 (s), 25.71 (s).

**Cyclohexyl benzoate:** GC: t_R_=18.11 min; Colorless liquid; Eluent (petroleum ether: ethyl acetate, 50: 1)

^1^H NMR (501 MHz, CDCl_3_) δ 8.08 (d, J = 7.8 Hz, 2H), 7.56 (t, J = 7.0 Hz, 1H), 7.45 (t, J = 7.6 Hz, 2H), 5.34 – 4.81 (m, 1H), 1.97 (d, J = 5.8 Hz, 2H), 1.86 – 1.77 (m, 2H), 1.62 (dd, J = 20.5, 11.1 Hz, 3H), 1.52 – 1.43 (m, 2H), 1.42 – 1.34 (m, 1H).

^13^C NMR (126 MHz, CDCl_3_) δ 166.00 (s), 132.67 (s), 131.05 (s), 129.53 (s), 128.26 (s), 73.03 (s), 31.65 (s), 25.50 (s), 23.67 (s).

**Oxolan-2-ylmethyl benzoate:** GC: t_R_=18.54 min; Light yellow liquid; Eluent (petroleum ether: ethyl acetate, 30: 1)

^1^H NMR (501 MHz, CDCl_3_) δ 8.09 (dd, J = 22.5, 5.9 Hz, 2H), 7.70 – 7.32 (m, 3H), 4.53 – 4.16 (m, 3H), 3.91 (ddd, J = 22.7, 16.3, 7.6 Hz, 2H), 1.93 (t, J = 84.3 Hz, 4H).

^13^C NMR (126 MHz, CDCl_3_) δ 166.58 (d, J = 5.0 Hz), 133.01 (d, J = 4.8 Hz), 130.12 (d, J = 3.8 Hz), 129.72 (d, J = 6.3 Hz), 128.37 (d, J = 6.1 Hz), 77.36 (d, J = 4.7 Hz), 68.58 (d, J = 2.7 Hz), 66.96 (d, J = 6.0 Hz), 28.14 (d, J = 6.5 Hz), 25.80 (d, J = 6.5 Hz).

**Benzoic acid phenyl ester:** GC: t_R_=19.54 min; White crystal powder; Eluent (petroleum ether: ethyl acetate, 30: 1)

^1^H NMR (501 MHz, CDCl_3_) δ 8.25 (d, J = 7.3 Hz, 2H), 7.67 (t, J = 7.4 Hz, 1H), 7.55 (t, J = 7.7 Hz, 2H), 7.47 (t, J = 7.9 Hz, 2H), 7.31 (t, J = 7.4 Hz, 1H), 7.25 (d, J = 7.7 Hz, 2H).

^13^C NMR (126 MHz, CDCl_3_) δ 165.22 (s), 151.01 (s), 133.60 (s), 130.20 (s), 129.57 (d, J = 13.9 Hz), 128.60 (s), 125.91 (s), 121.75 (s).

**Benzyl benzoate:** GC: t_R_=20.36 min; Colorless liquid; Eluent (petroleum ether: ethyl acetate, 30: 1)

^1^H NMR (501 MHz, CDCl_3_) δ 8.13 (d, J = 7.4 Hz, 2H), 7.59 (t, J = 7.4 Hz, 1H), 7.52 – 7.44 (m, 5H), 7.44 – 7.33 (m, 2H), 5.41 (s, 2H).

^13^C NMR (126 MHz, CDCl_3_) δ 166.47 (s), 136.11 (s), 133.07 (s), 130.19 (s), 129.75 (s), 128.64 (s), 128.52 – 128.13 (m), 66.65 (s).

**1,6-O,O-diacetylbritannilactone:** White crystals; Eluent (petroleum ether: ethyl acetate, 30: 1)

^1^H NMR (399 MHz, CDCl_3_) δ 6.38 (d, J = 2.7 Hz, 1H), 5.94 (d, J = 2.3 Hz, 1H), 5.21 (d, J = 1.7 Hz, 1H), 4.99 – 4.90 (m, 1H), 3.93 (qd, J = 11.0, 5.8 Hz, 2H), 3.49 (s, 1H), 2.70 (ddd, J = 10.3, 6.8, 2.5 Hz, 2H), 2.49 (dd, J = 16.1, 2.1 Hz, 1H), 2.05 (d, J = 1.9 Hz, 6H), 1.80 (s, 3H), 1.45 – 1.35 (m, 1H), 1.28 – 1.24 (m, 2H), 1.07 – 0.98 (m, 1H), 0.88 (d, J = 6.9 Hz, 3H).

^13^C NMR (100 MHz, CDCl_3_) δ 171.25 (s), 170.92 (s), 169.55 (s), 136.28 (s), 133.85 (s), 132.03 (s), 125.06 (s), 74.98 (s), 69.26 (s), 64.26 (s), 42.90 (s), 34.57 (s), 33.09 (s), 31.10 (s), 26.51 (s), 21.32 (s), 21.01 (s), 20.54 (s), 18.46 (s).

**Supplementary Figures**

**Figure S1. The process of catalyst preparation.**

**Figure S2. The FTIR spectra of (NH_4_)_3_[CoMo_6_O_18_(OH)_6_].**

**Figure S3. The XRD spectra of (NH_4_)_3_[CoMo_6_O_18_(OH)_6_].**


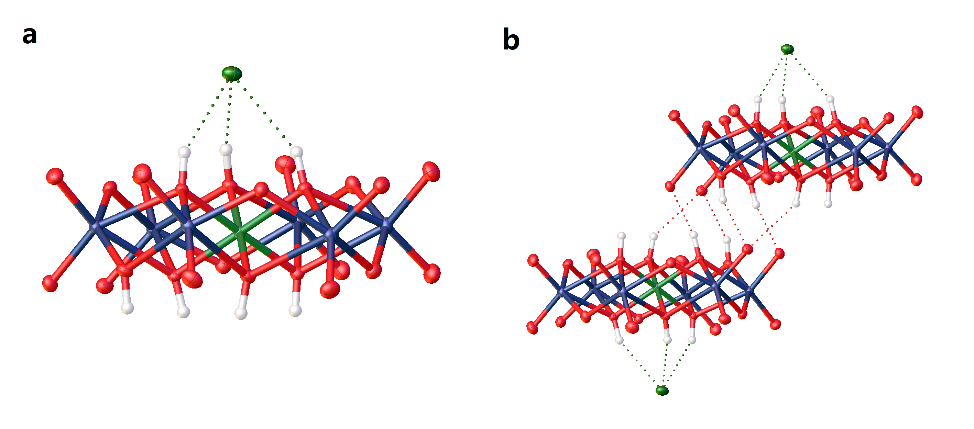


**Figure S4. Cluster structure of the monomer (a) and dimer (b).** Red: O. Green: Cl. Light geen: Co. Blue: Mo. White: H.

**Figure S5.** **Cyclic voltammogram experiments.** Cyclic voltammograms (298 K, scan rate 100mVs^-1^) of a 1.0 mM acetonitrile solution of the CoMo_6_ in the presence of KCl (10 mM).

**Figure S6. Gram scale reactions.**

**Figure S7. Recycling of cobalt catalyst for oxidative esterification of benzyl alcohol and methanol.**

**Figure S8.** **The FTIR spectra of the catalyst before and after the sixth reaction.**

**Figure S9.** **The XRD spectra of the catalyst before and after the sixth reaction. (a)** Fresh. **(b)** The sixth cycling.


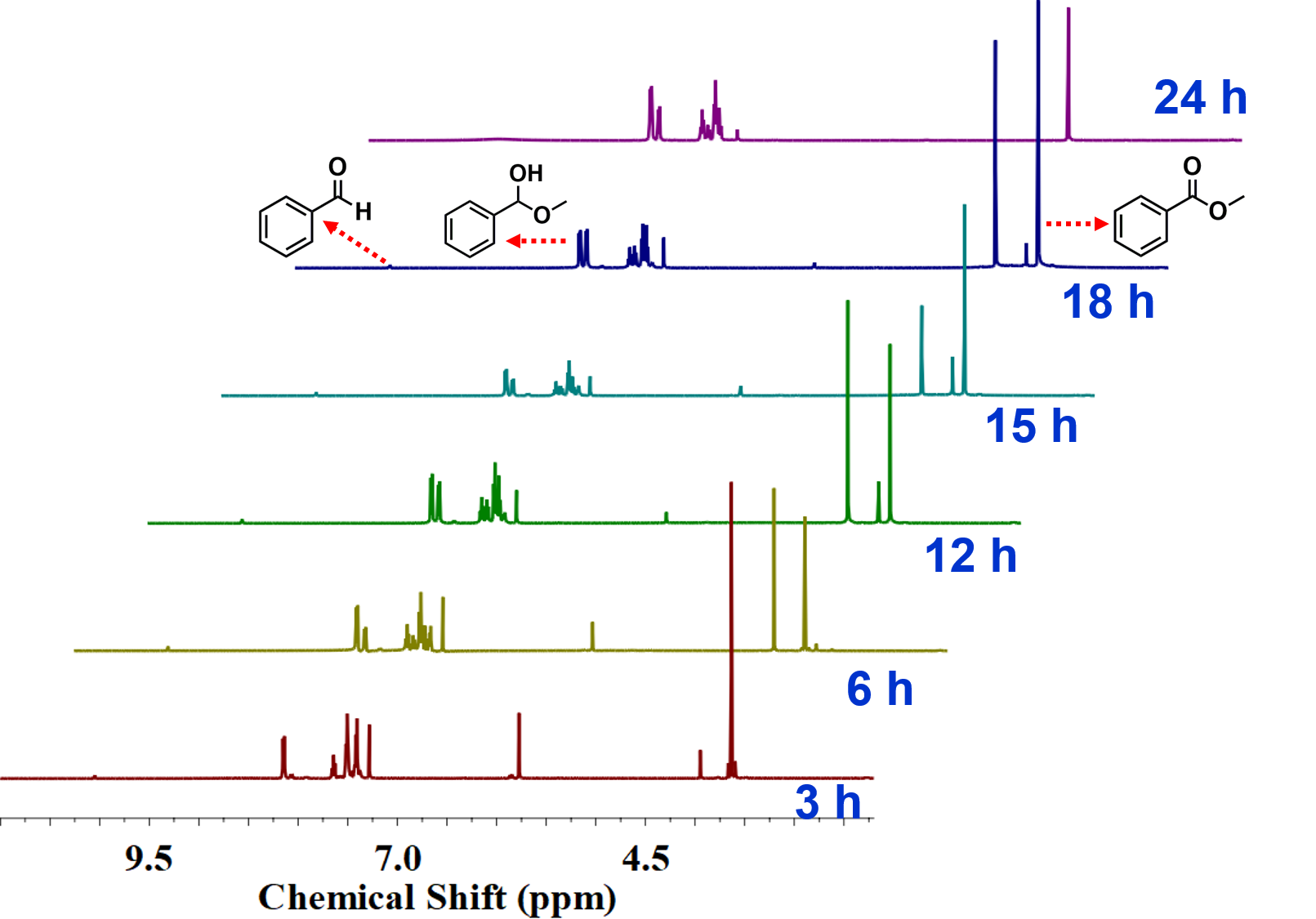


**Figure S10.** ^1^H NMR spectral study of the oxidation of benzaldehyde and methanol in the presence of KCl with time evolving from 3h to 24 h.


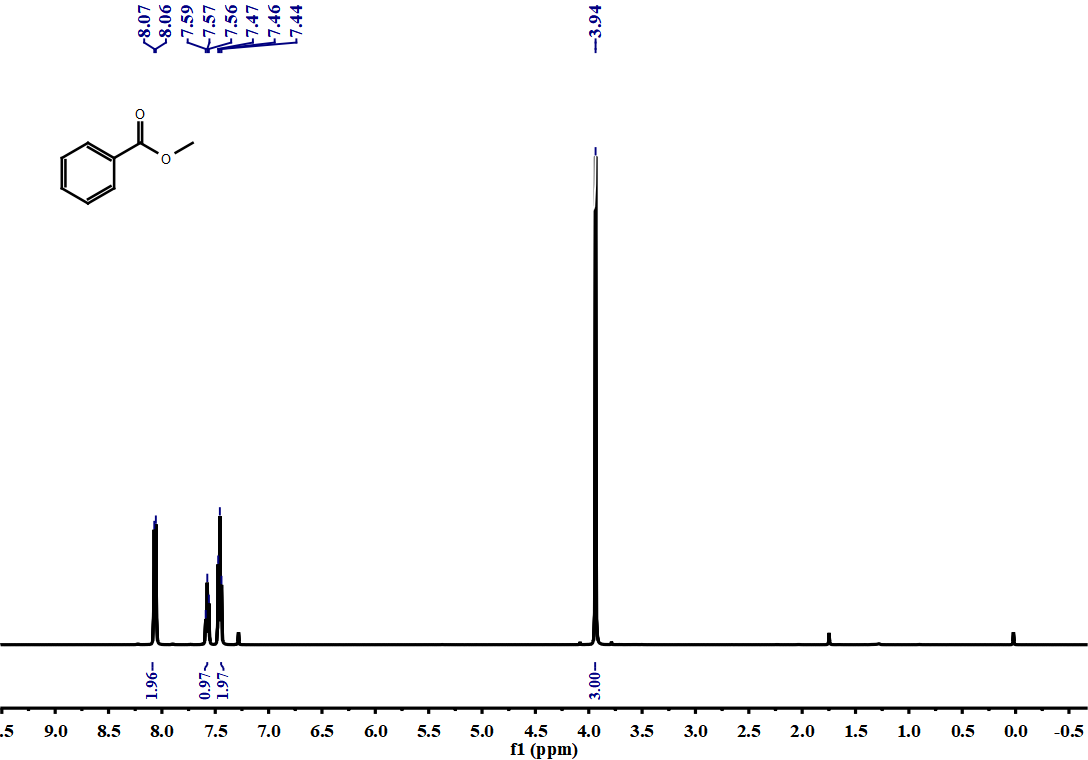


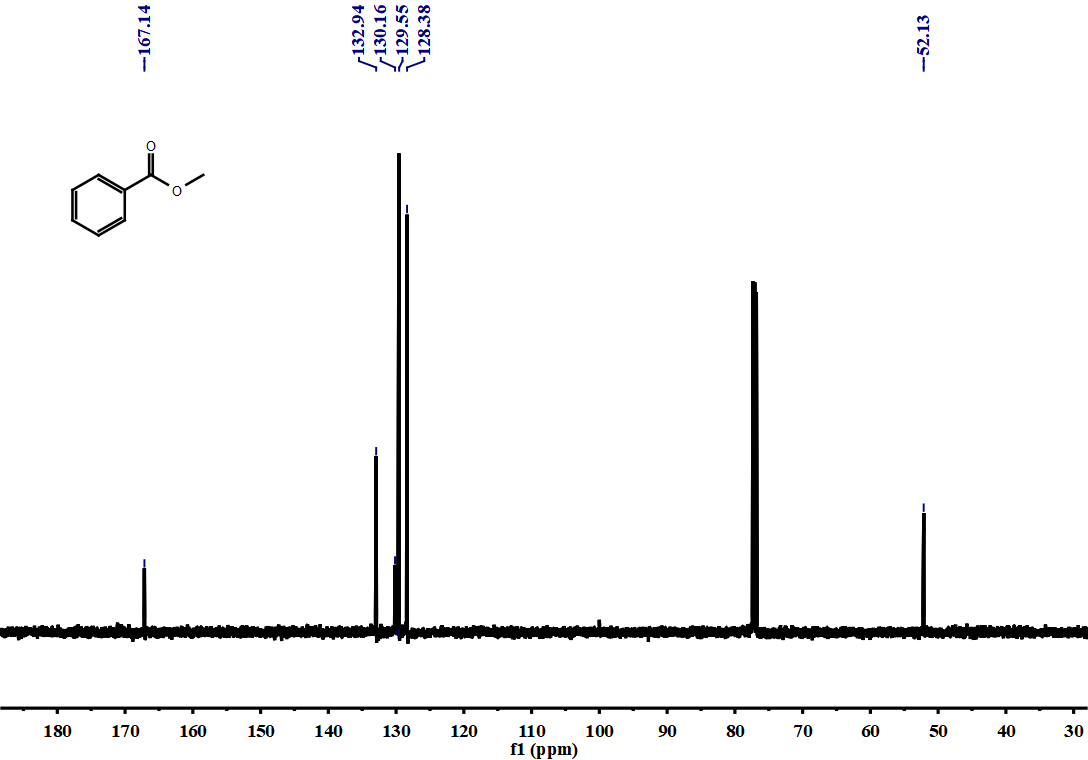


**Figure S11.** ^1^H NMR (top) and ^13^C NMR (bottom) of **2** (CDCl_3_ as the solvent).


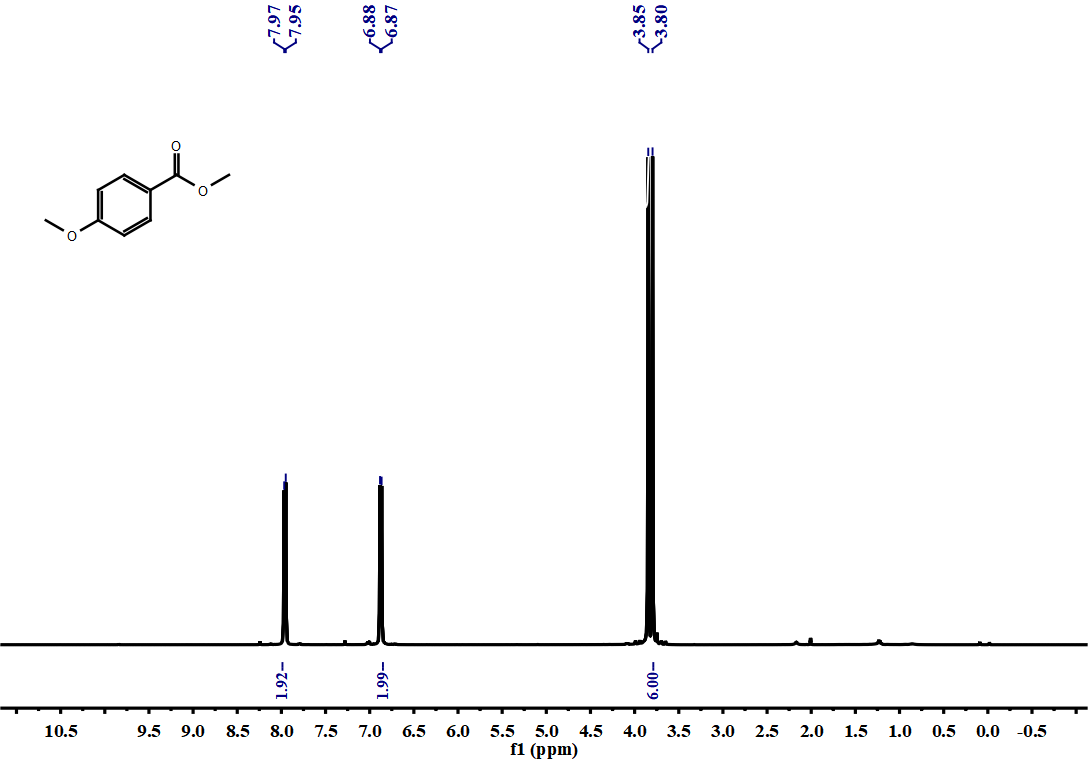


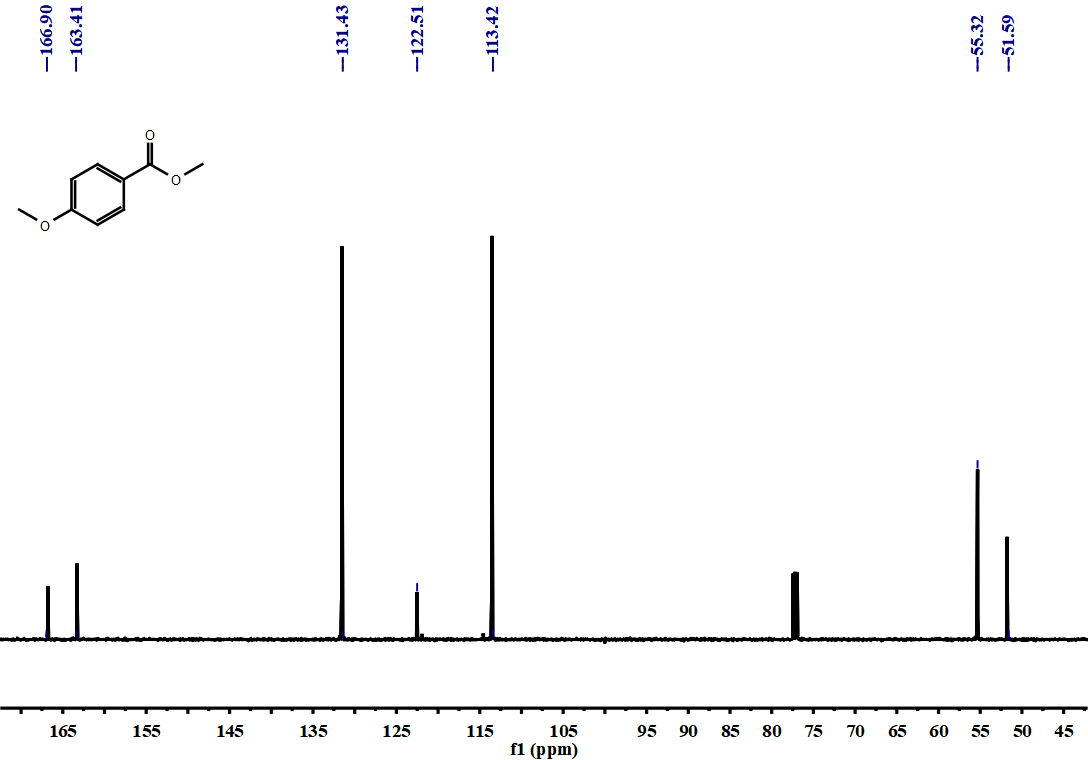


**Figure S12.** ^1^H NMR (top) and ^13^C NMR (bottom) of **3** (CDCl_3_ as the solvent).


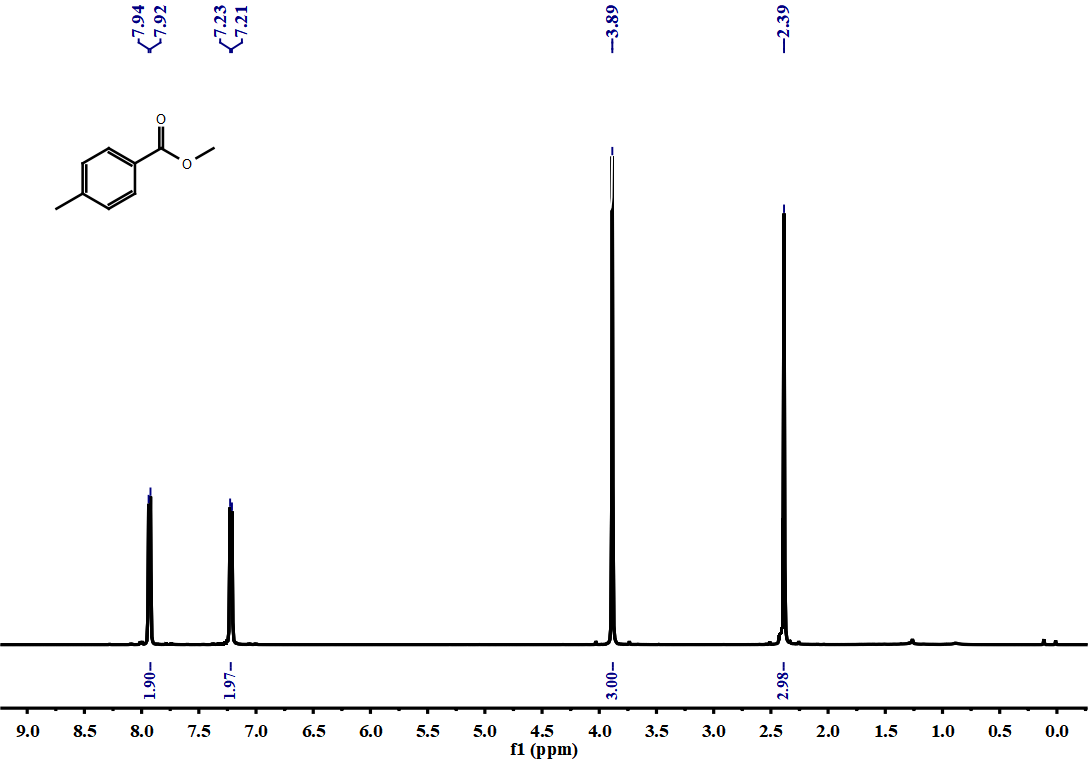


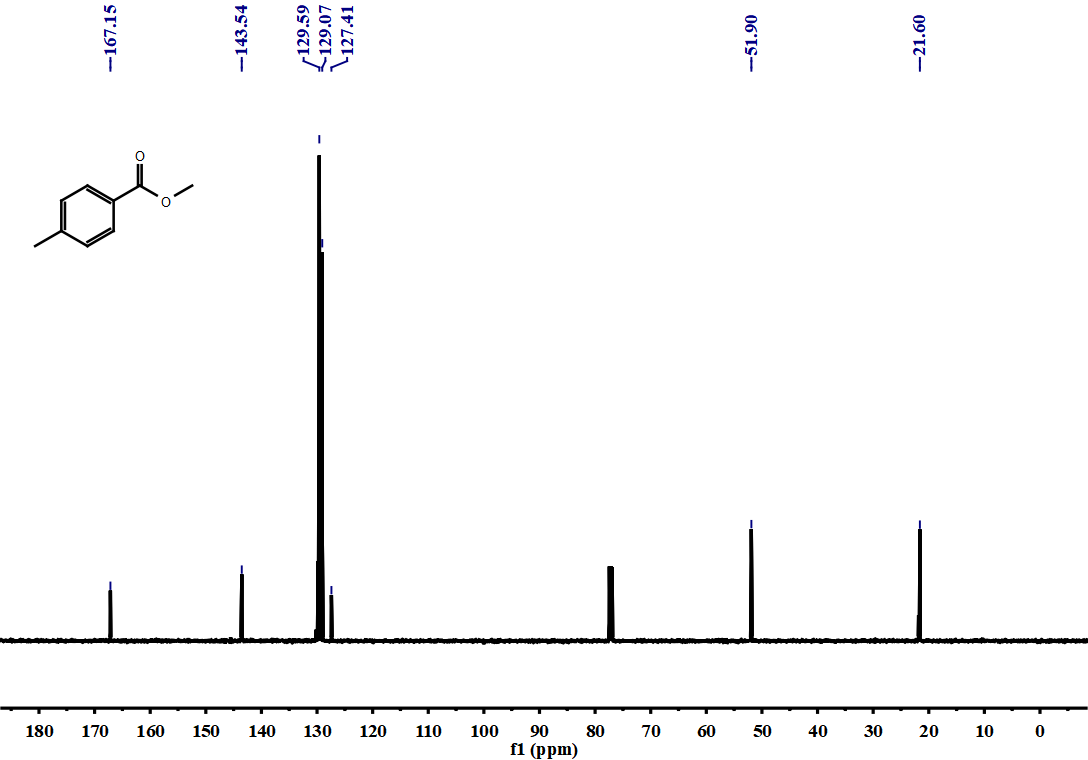


**Figure S13.** ^1^H NMR (top) and ^13^C NMR (bottom) of **4** (CDCl_3_ as the solvent).


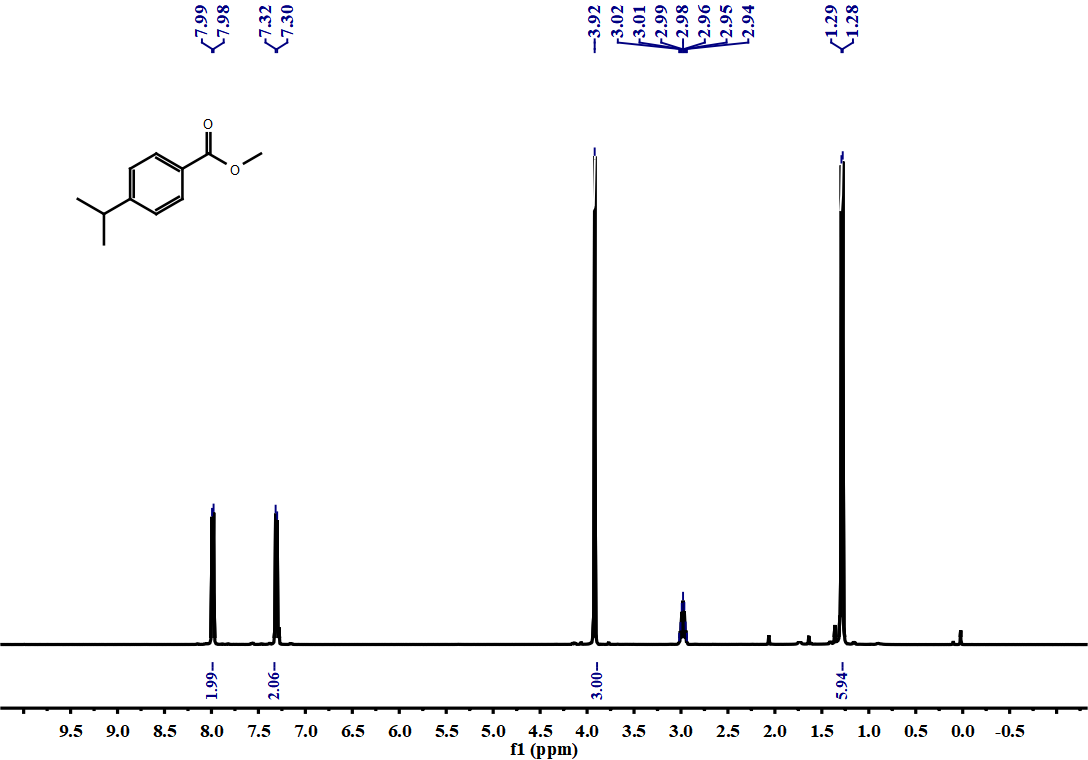


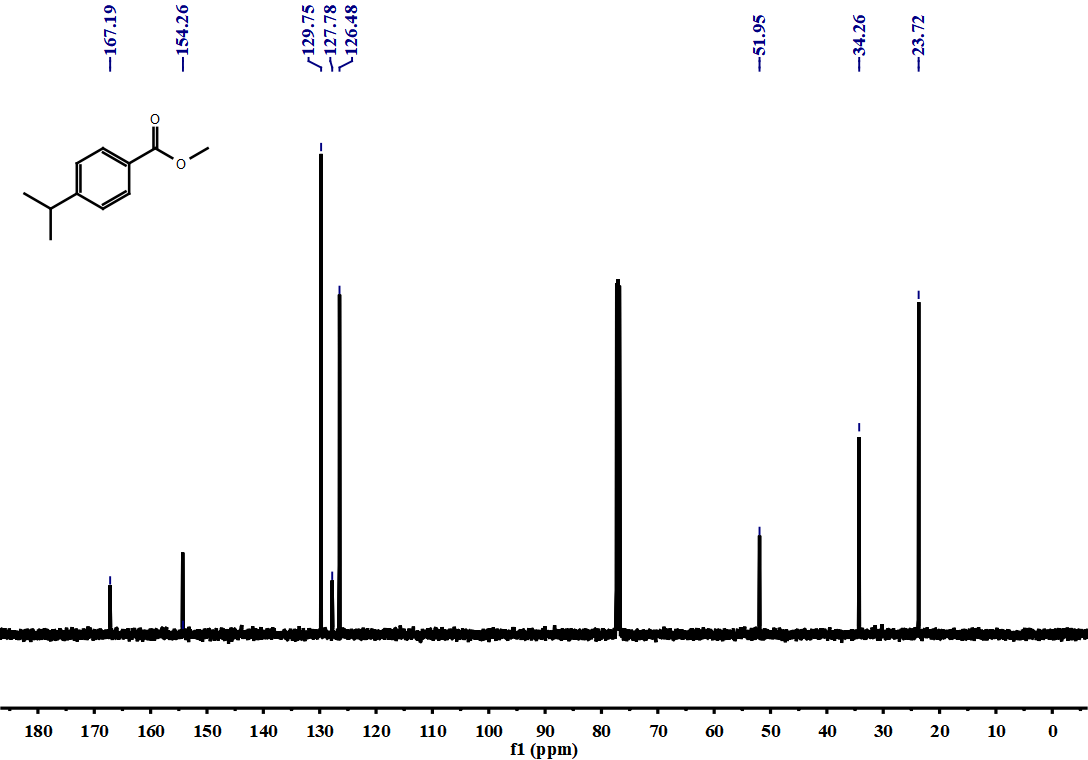


**Figure S14.** ^1^H NMR (top) and ^13^C NMR (bottom) of **5** (CDCl_3_ as the solvent).


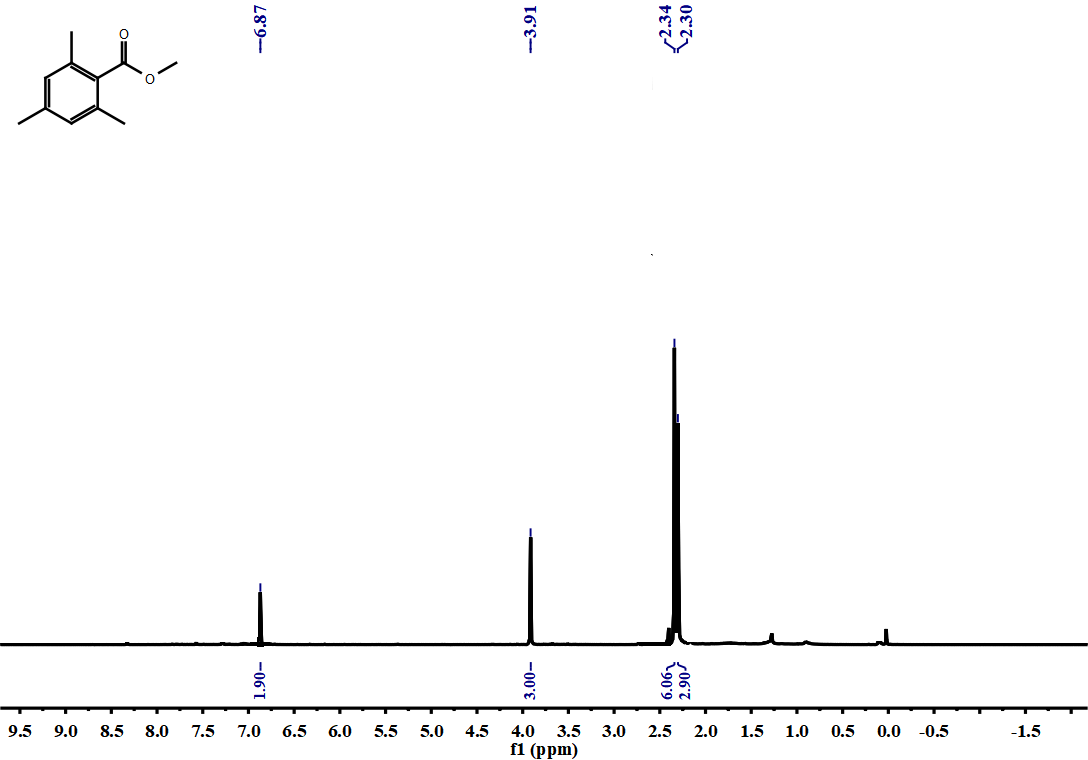


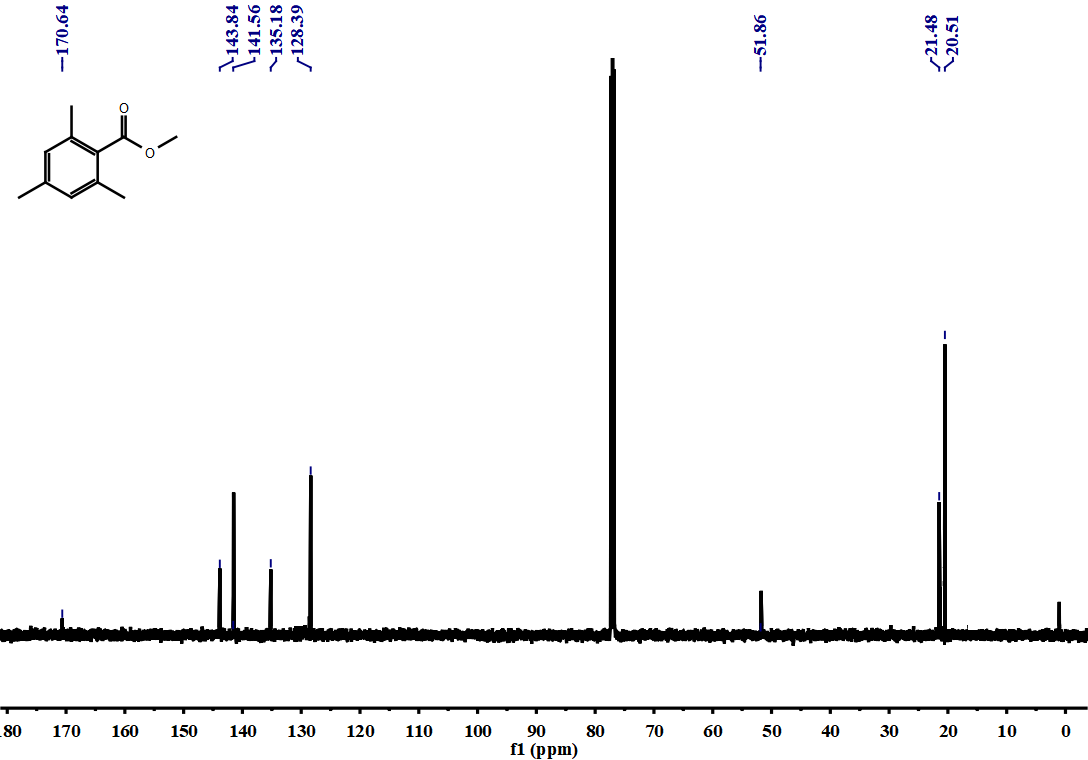


**Figure S15.** ^1^H NMR (top) and ^13^C NMR (bottom) of **6** (CDCl_3_ as the solvent).


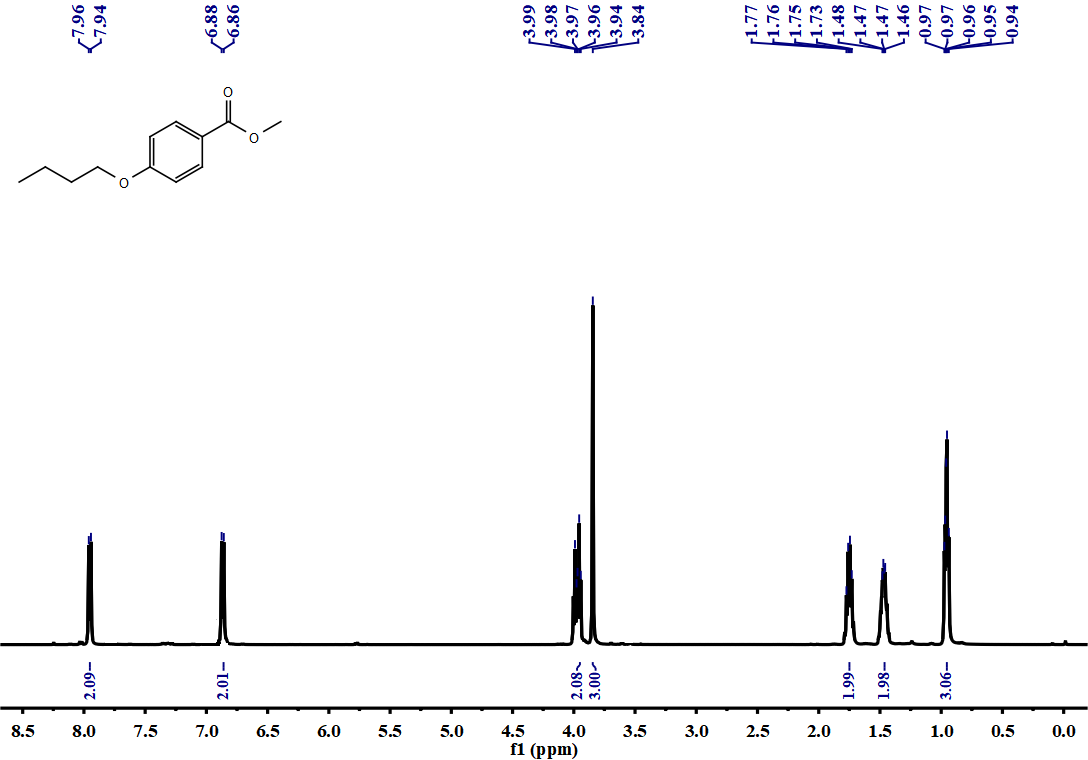


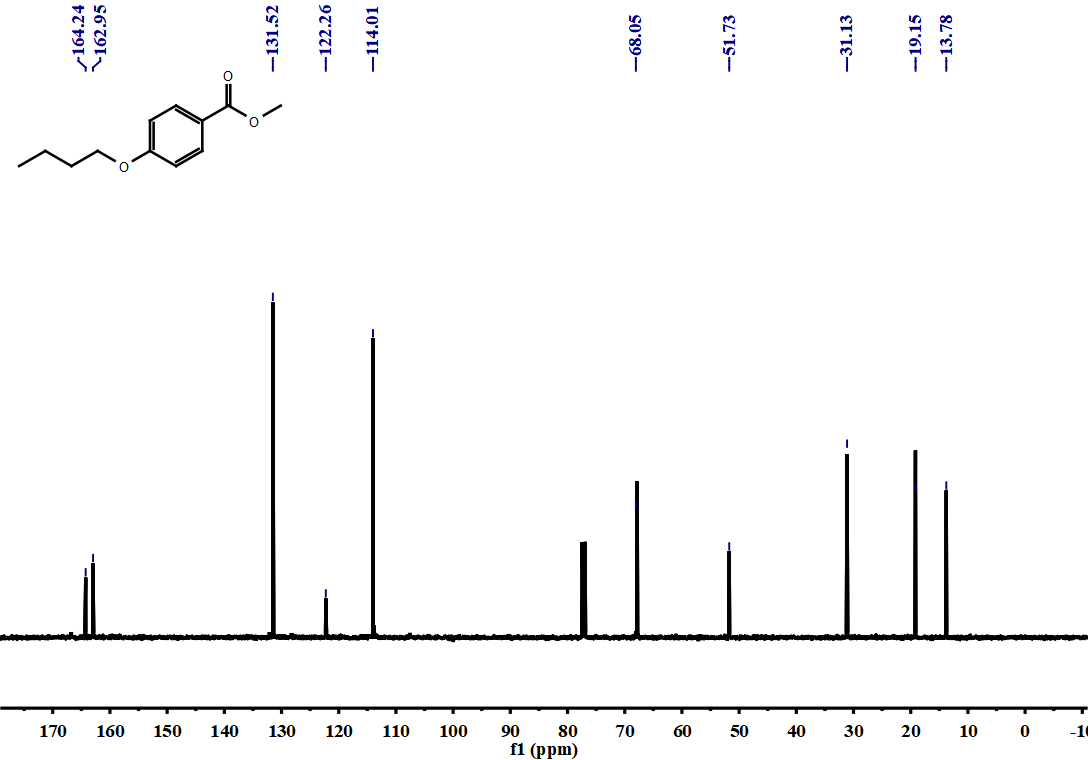


**Figure S16.** ^1^H NMR (top) and ^13^C NMR (bottom) of **7** (CDCl_3_ as the solvent).


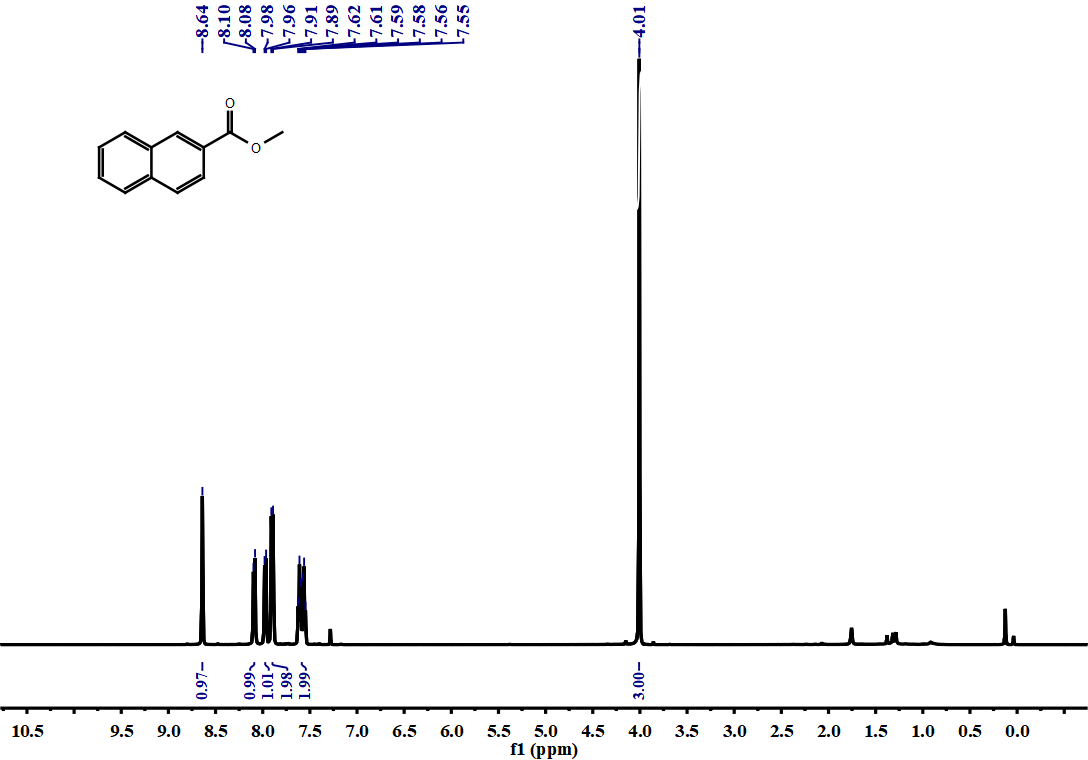


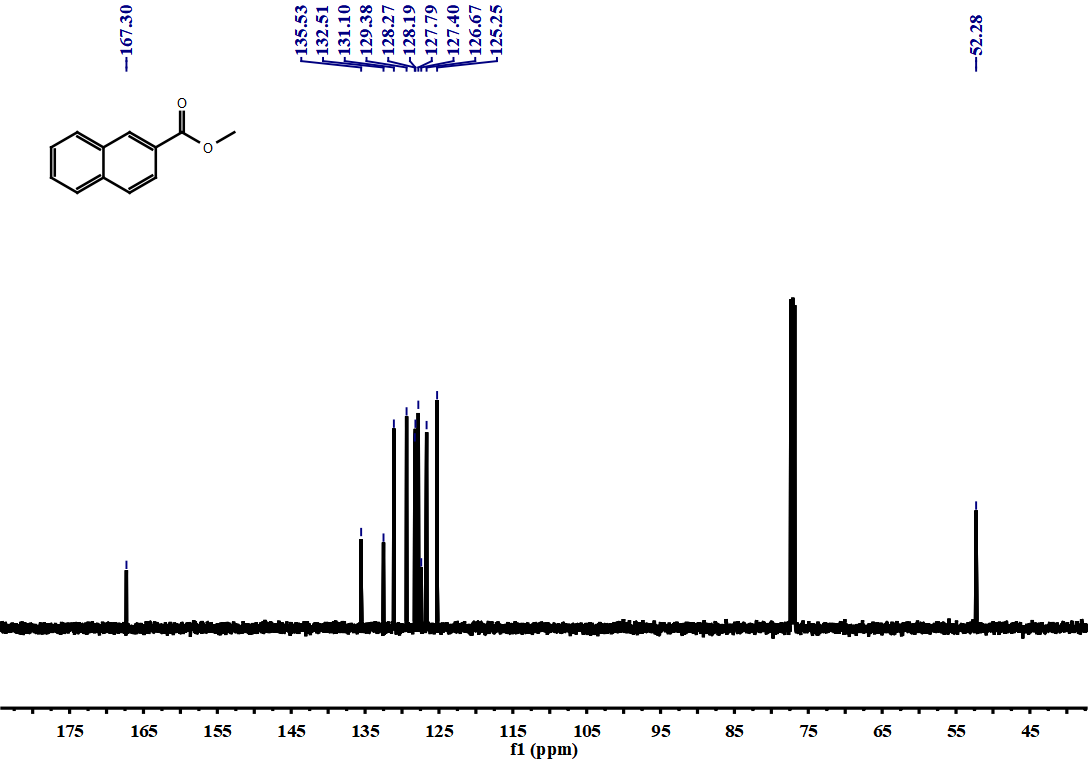


**Figure S17.** ^1^H NMR (top) and ^13^C NMR (bottom) of **8** (CDCl_3_ as the solvent).


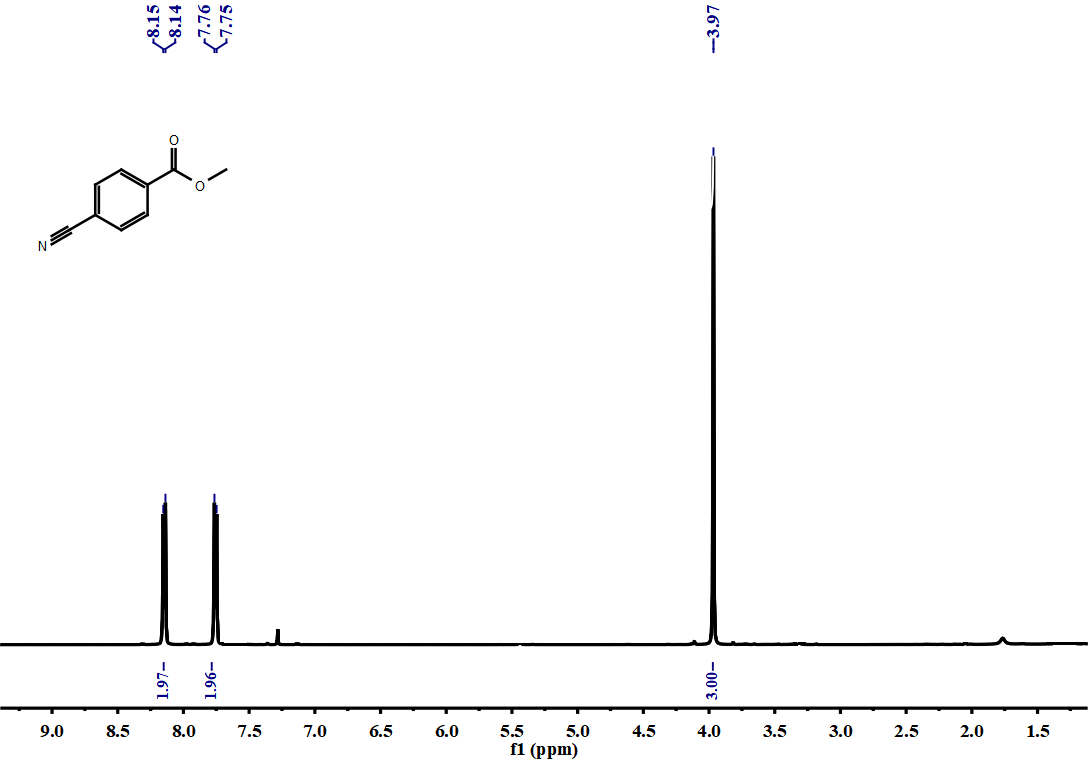


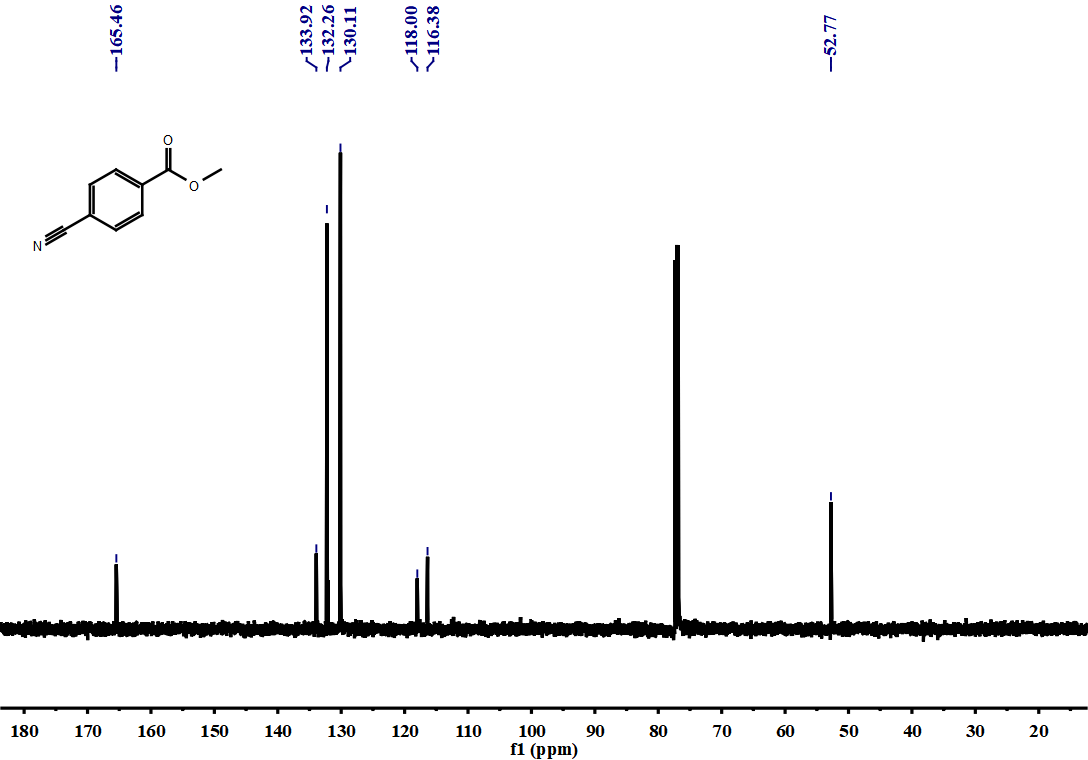


**Figure S18.** ^1^H NMR (top) and ^13^C NMR (bottom) of **9** (CDCl_3_ as the solvent).


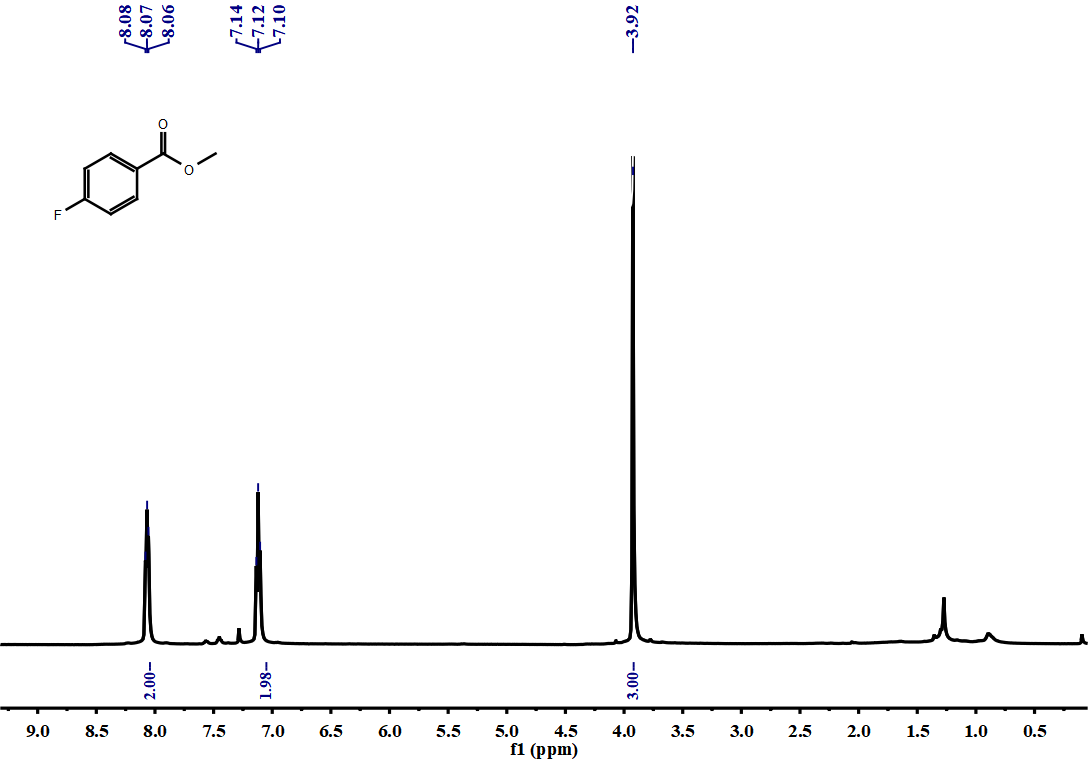


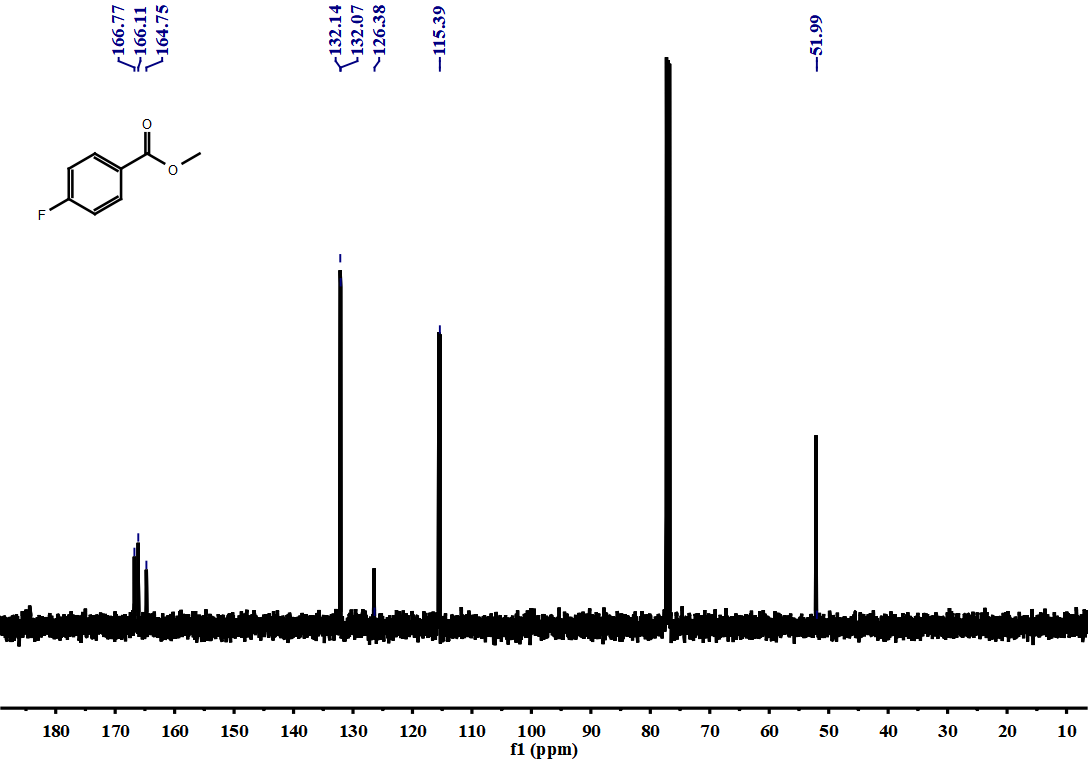


**Figure S19.** ^1^H NMR (top) and ^13^C NMR (bottom) of **10** (CDCl_3_ as the solvent).


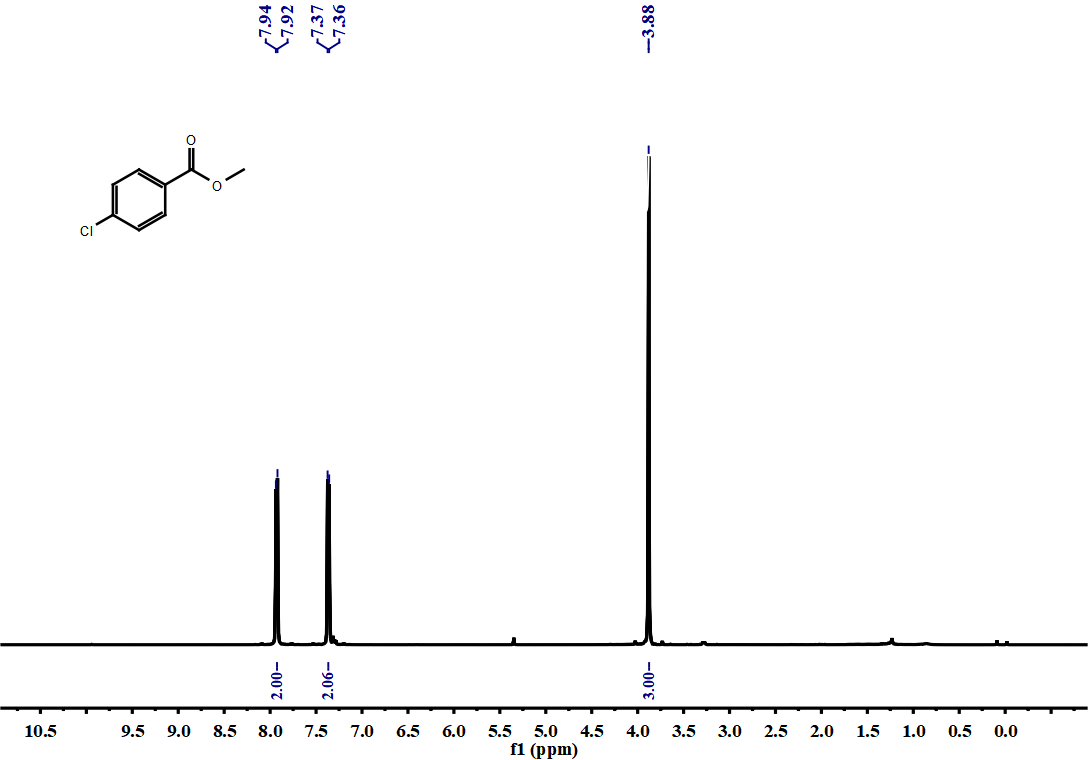


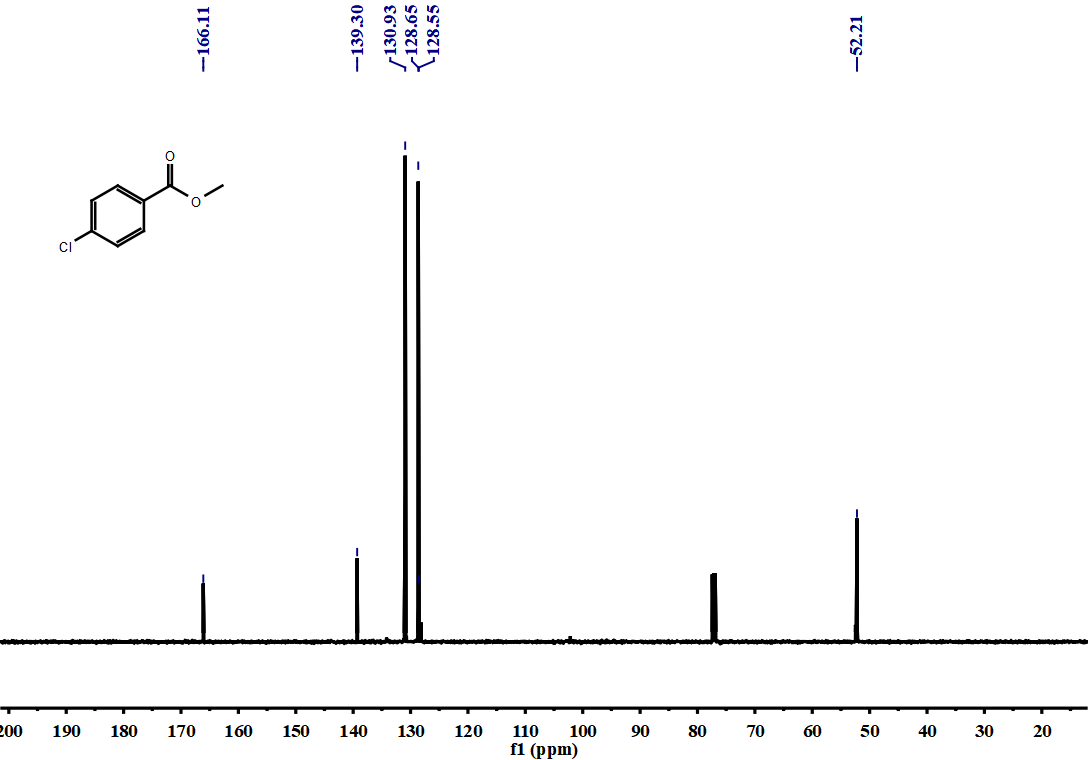


**Figure S20.** ^1^H NMR (top) and ^13^C NMR (bottom) of **11** (CDCl_3_ as the solvent).


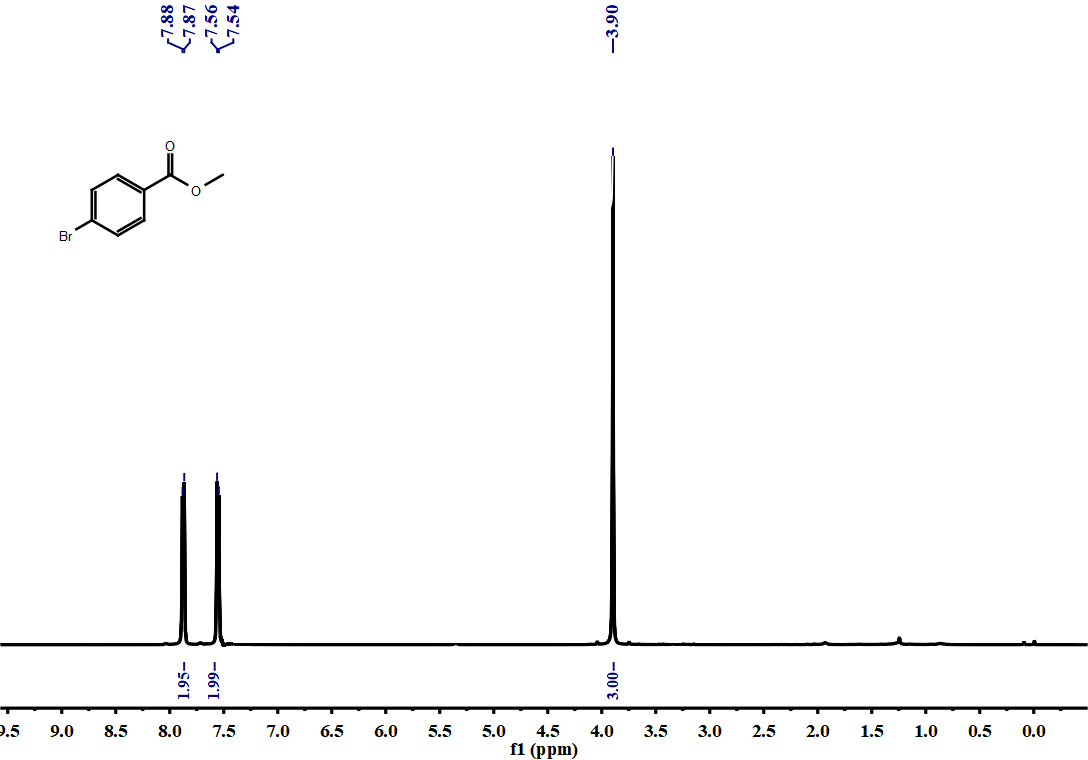


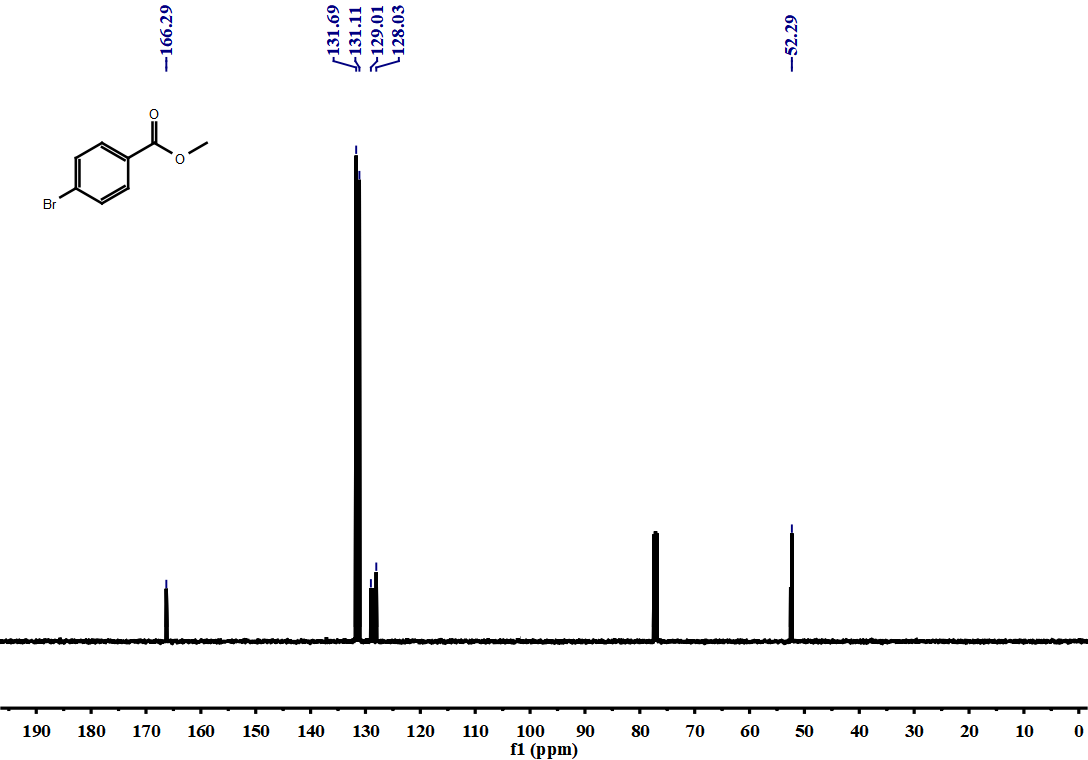


**Figure S21.** ^1^H NMR (top) and ^13^C NMR (bottom) of **12** (CDCl_3_ as the solvent).

**
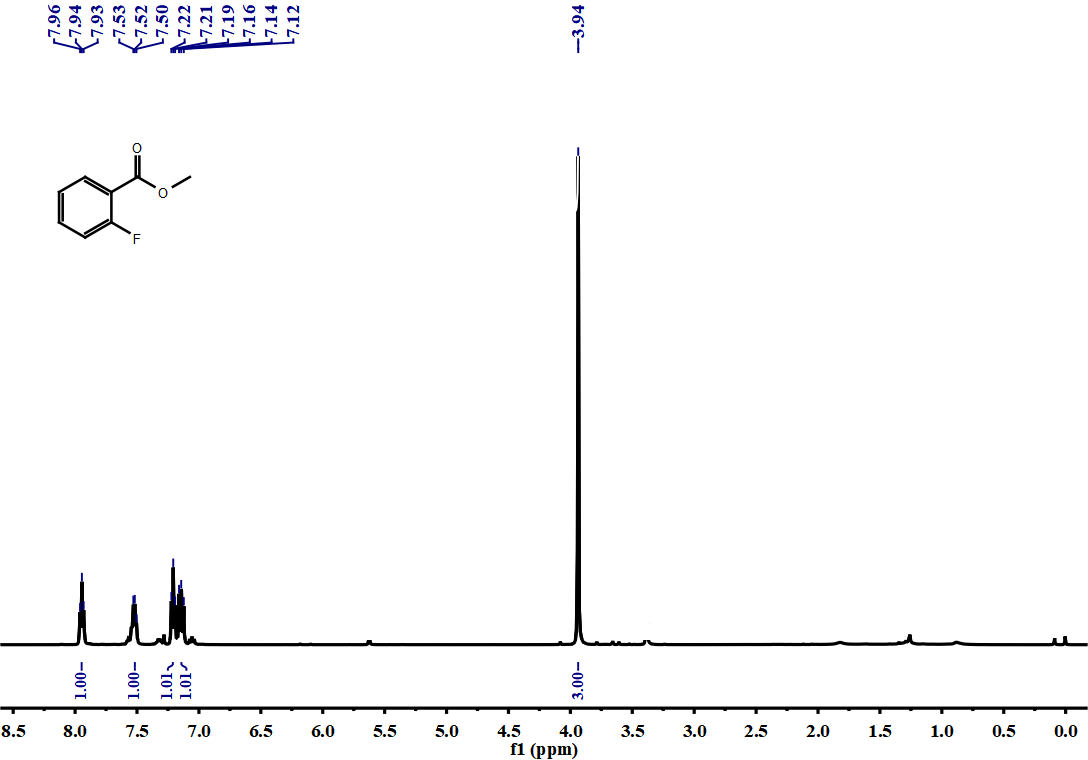
**

**
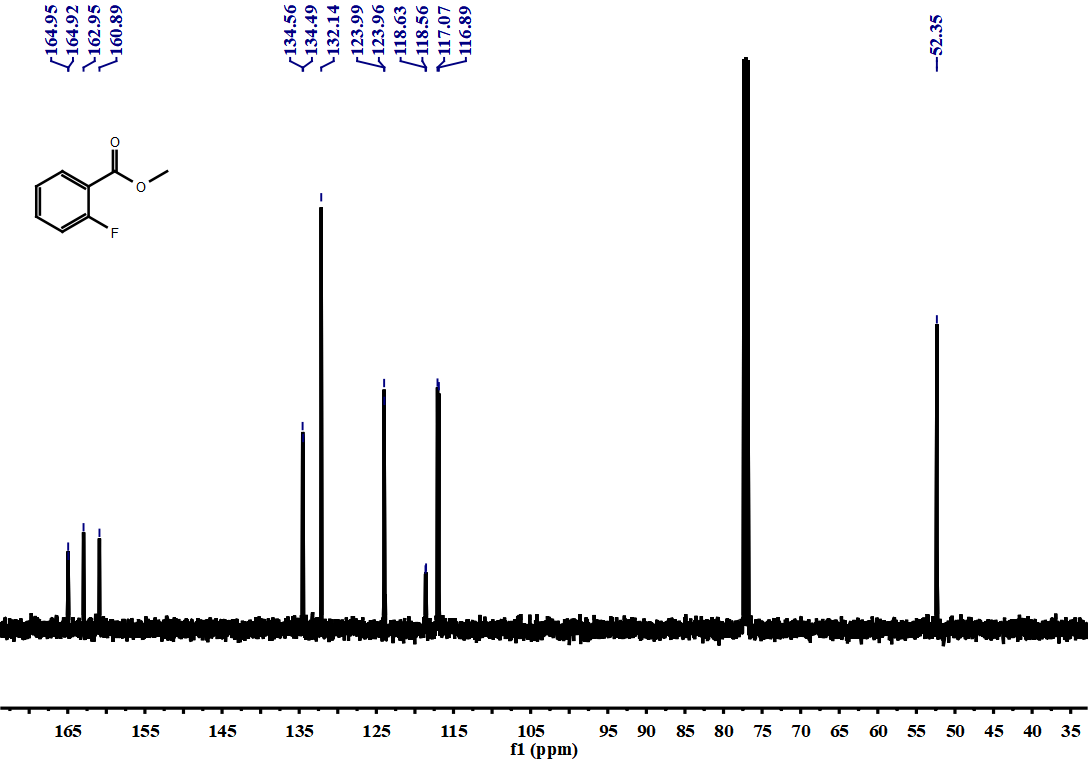
**

**Figure S22.** ^1^H NMR (top) and ^13^C NMR (bottom) of **13** (CDCl_3_ as the solvent).

**
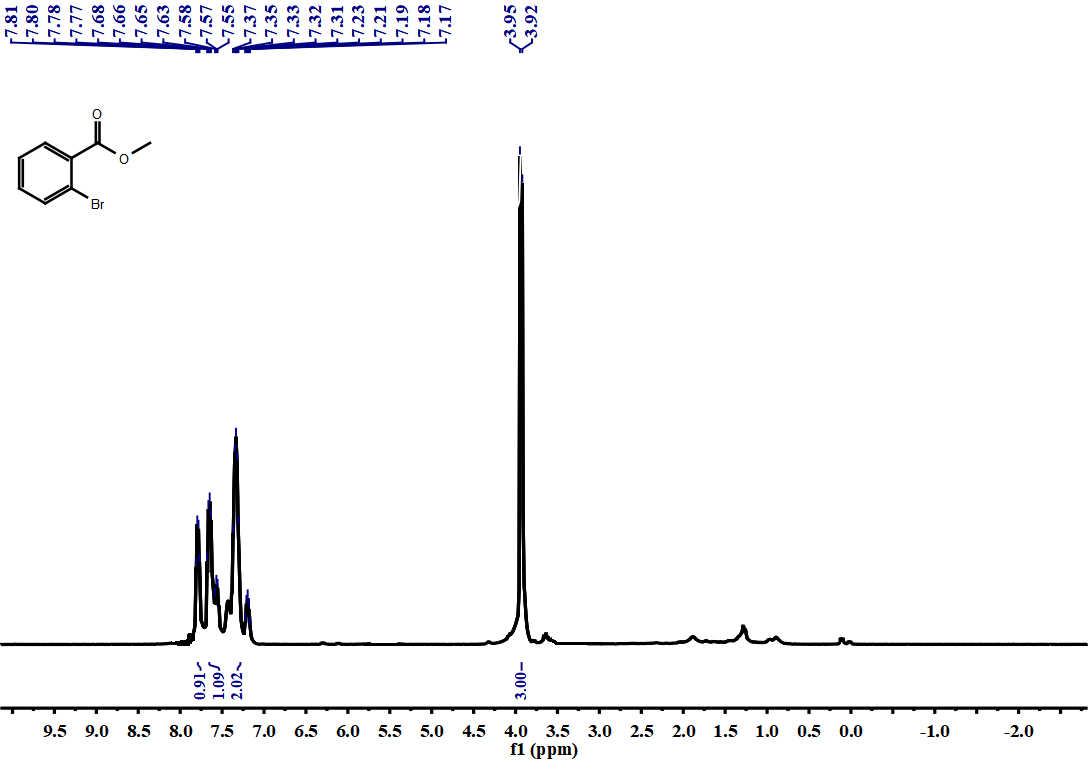
**


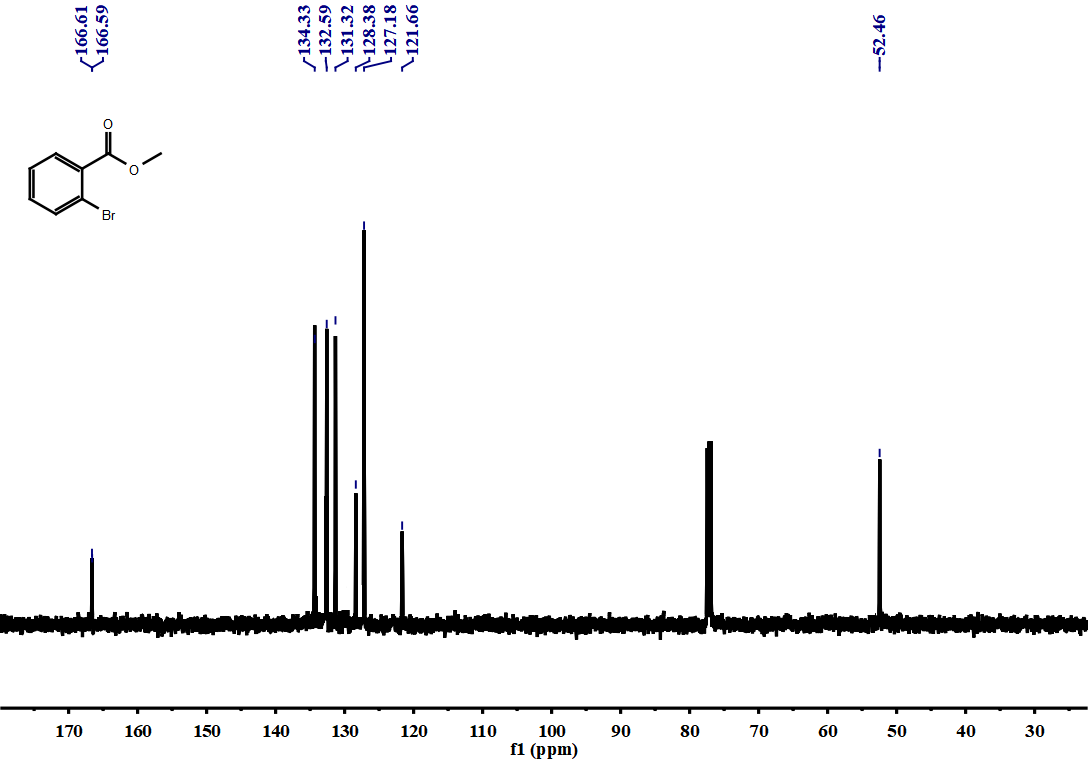


**Figure S23.** ^1^H NMR (top) and ^13^C NMR (bottom) of **14** (CDCl_3_ as the solvent).


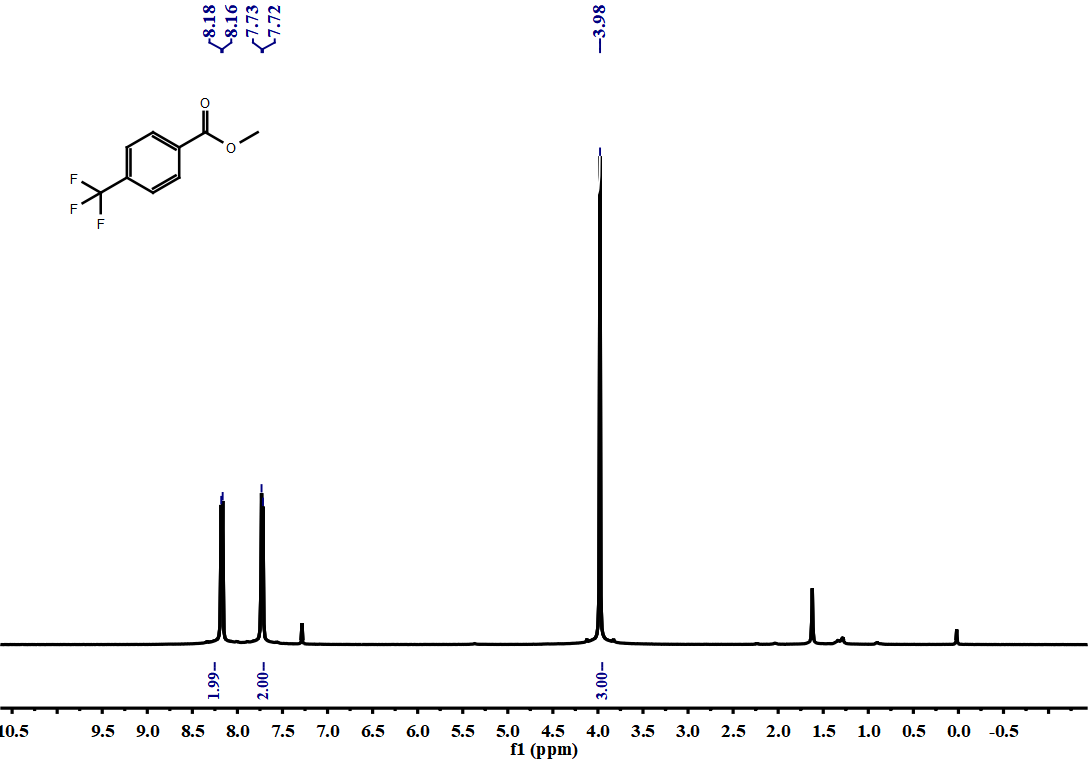


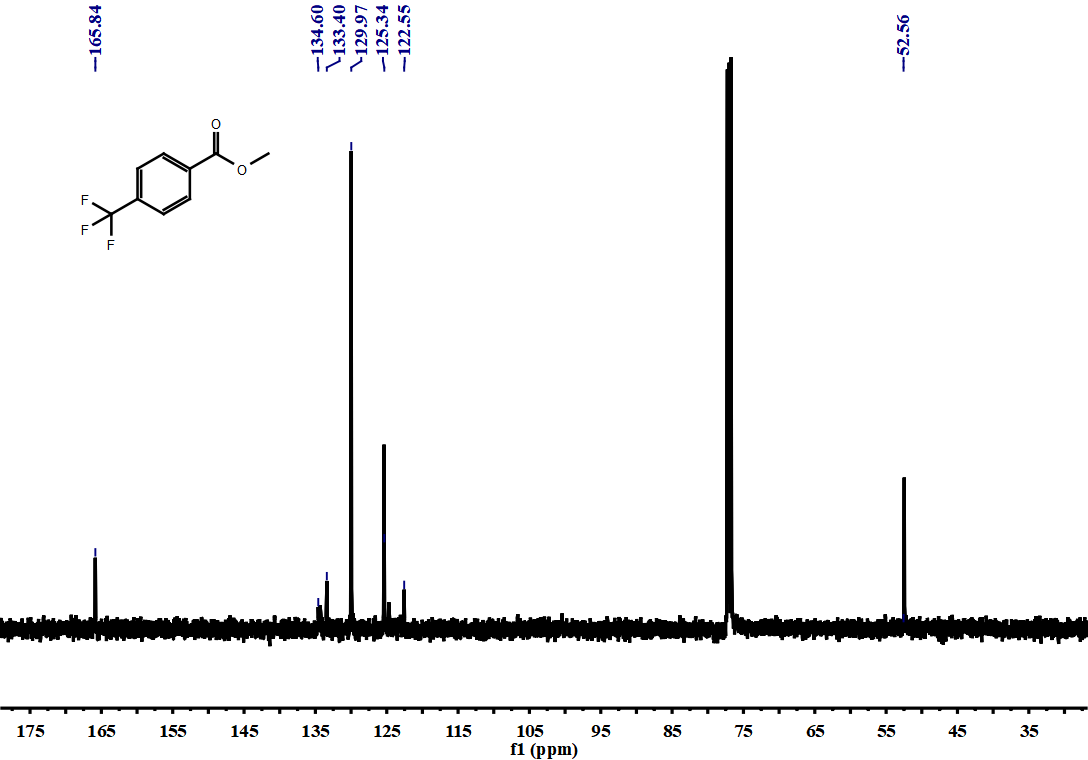


**Figure S24.** ^1^H NMR (top) and ^13^C NMR (bottom) of **15** (CDCl_3_ as the solvent).


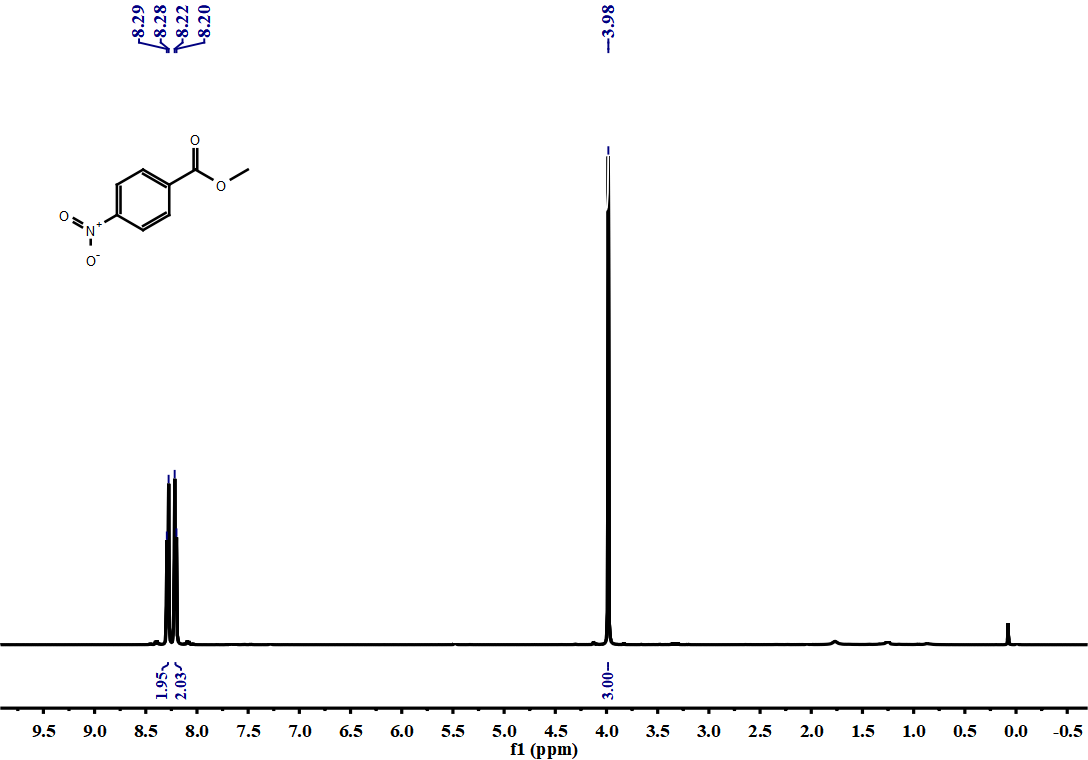

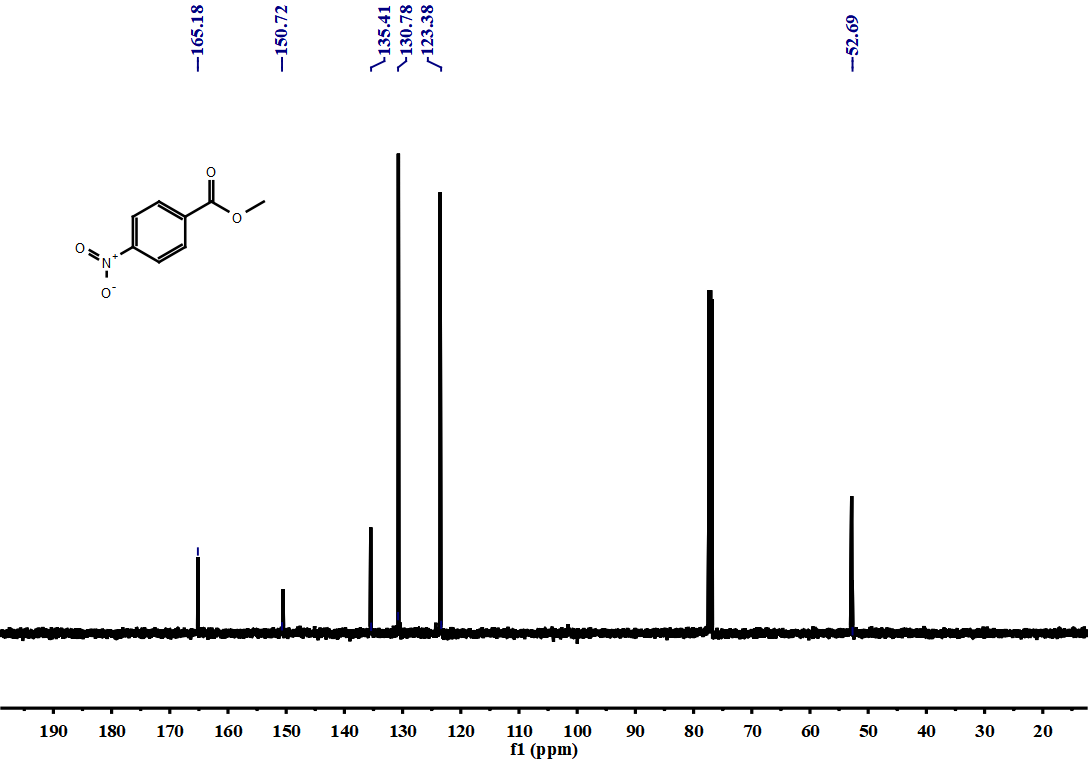


**Figure S25.** ^1^H NMR (top) and ^13^C NMR (bottom) of **16** (CDCl_3_ as the solvent).


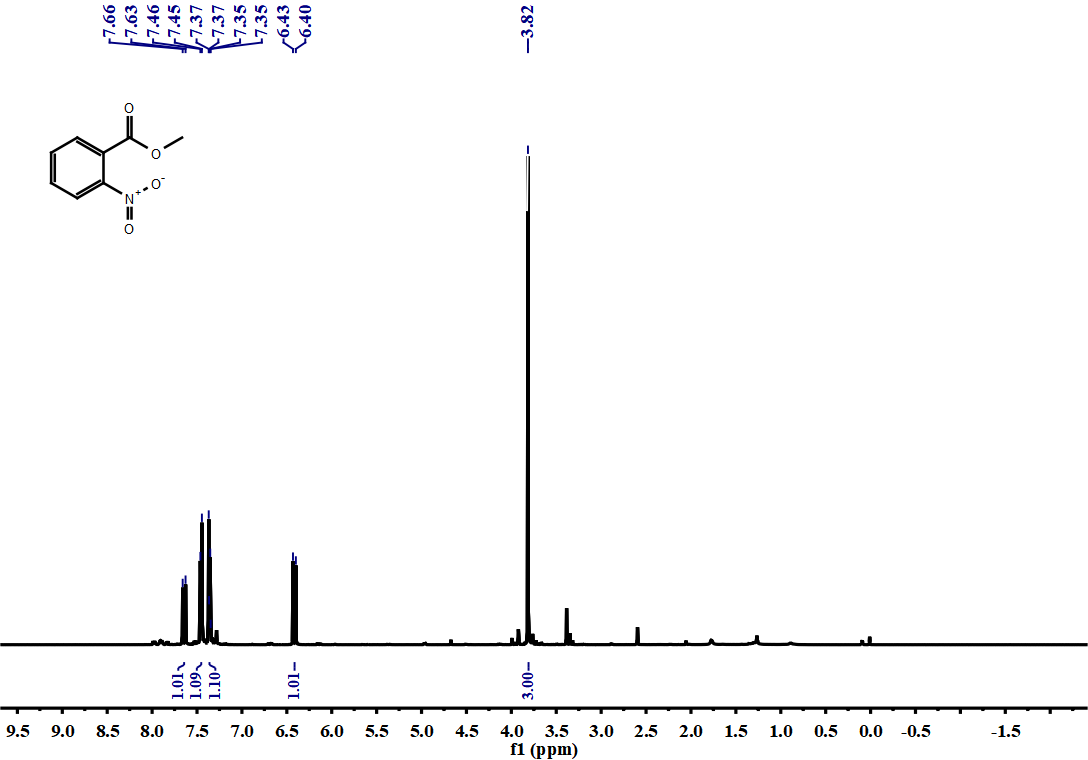


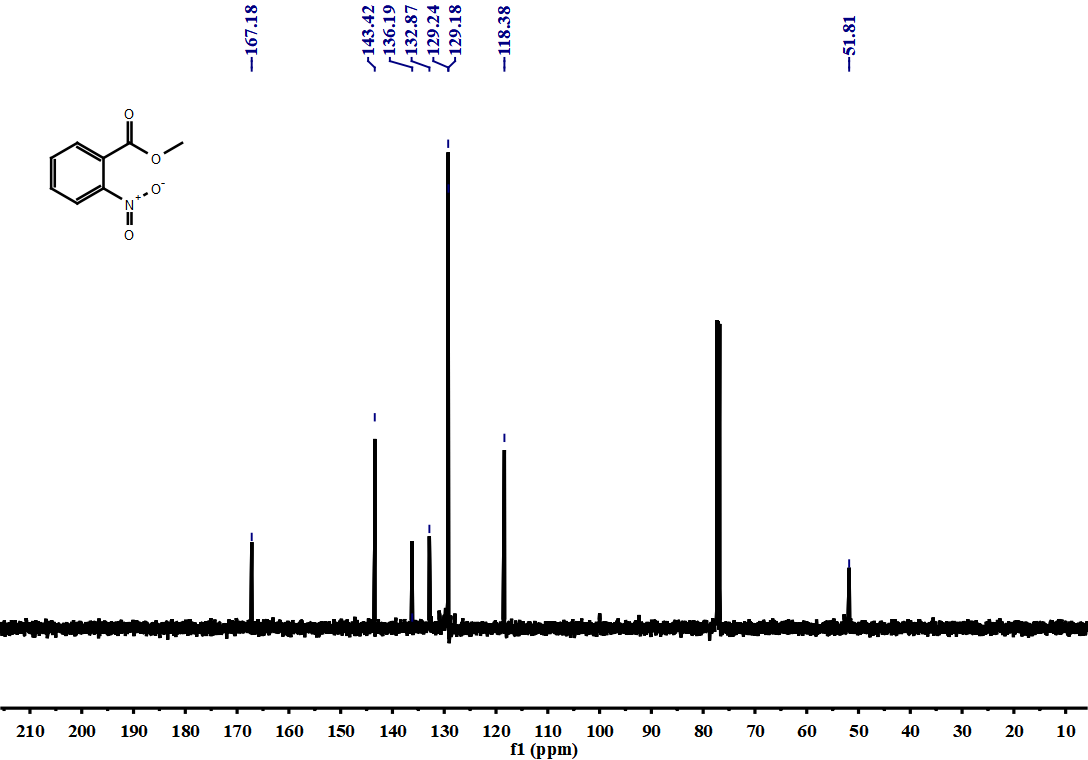


**Figure S26.** ^1^H NMR (top) and ^13^C NMR (bottom) of **17** (CDCl_3_ as the solvent).


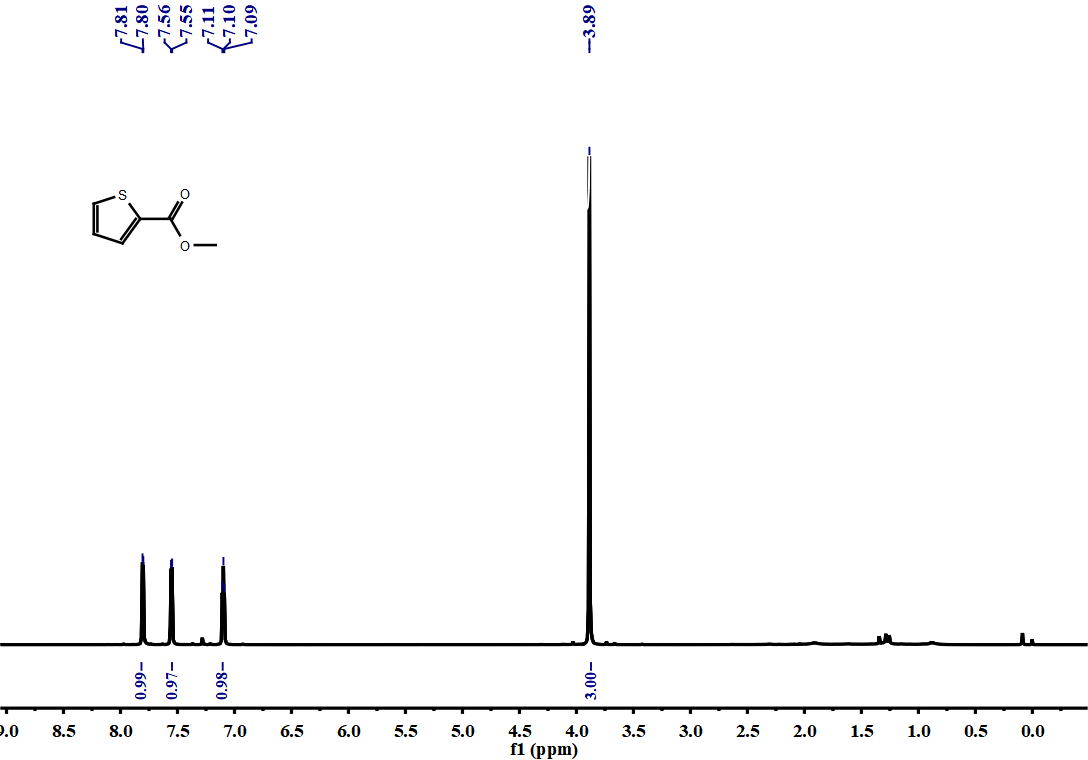


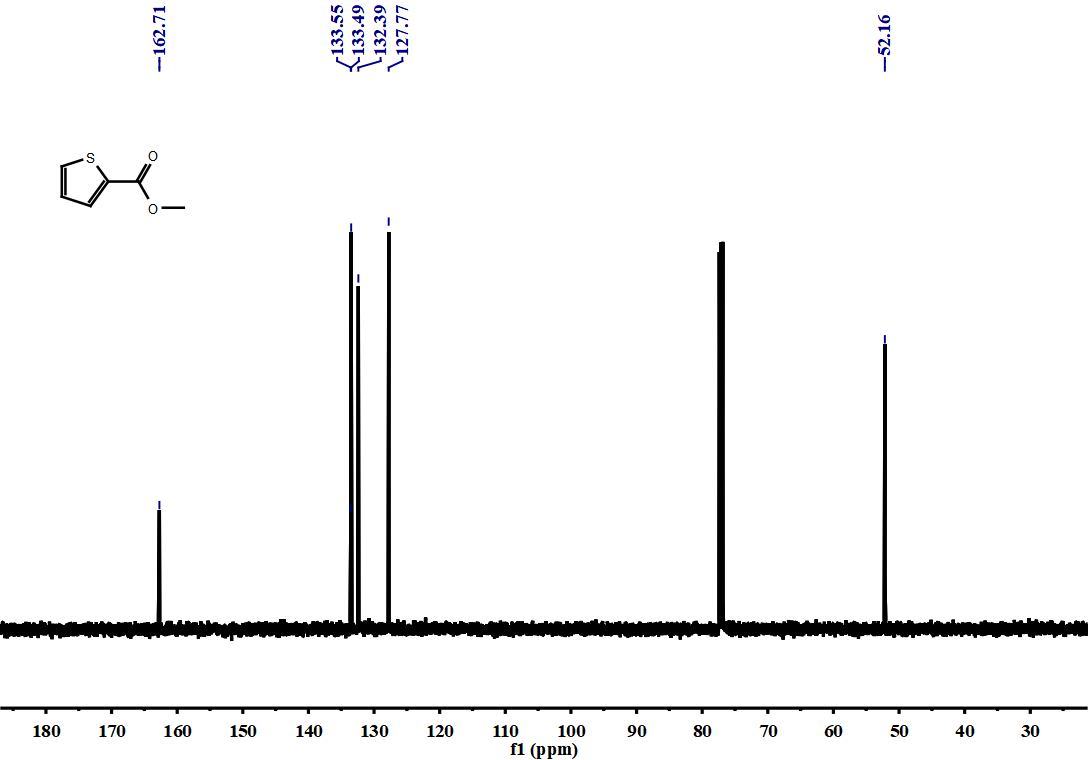


**Figure S27.** ^1^H NMR (top) and ^13^C NMR (bottom) of **18** (CDCl_3_ as the solvent).


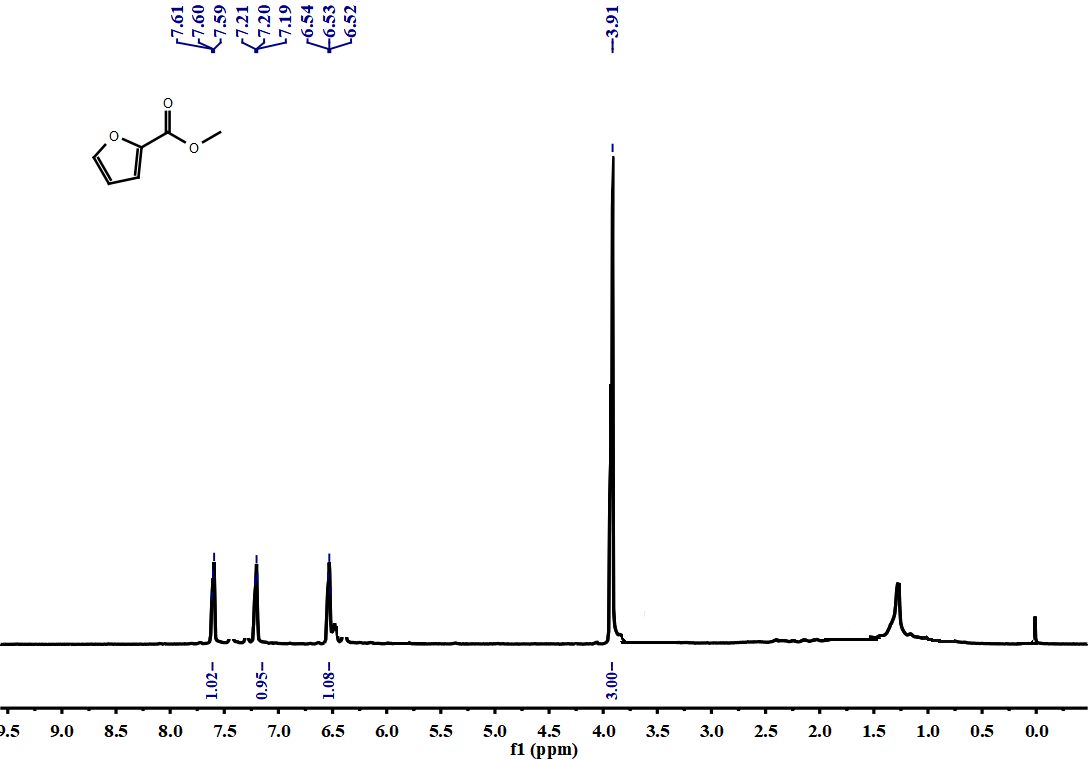


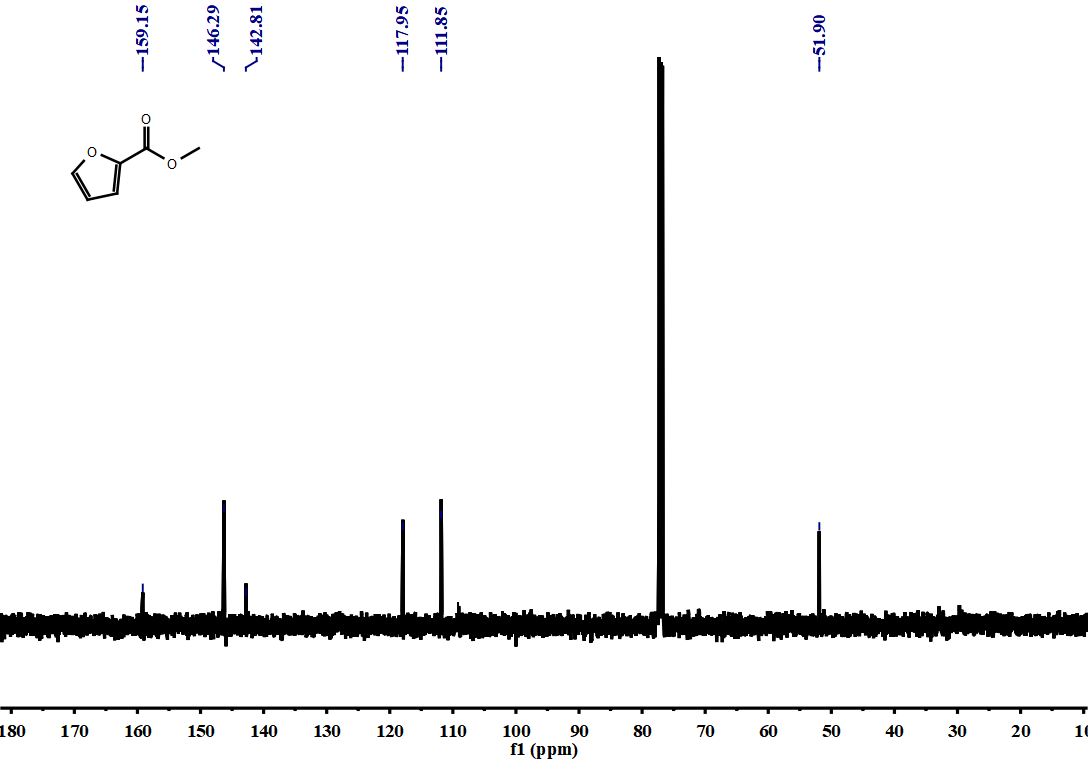


**Figure S28.** ^1^H NMR (top) and ^13^C NMR (bottom) of **19** (CDCl_3_ as the solvent).


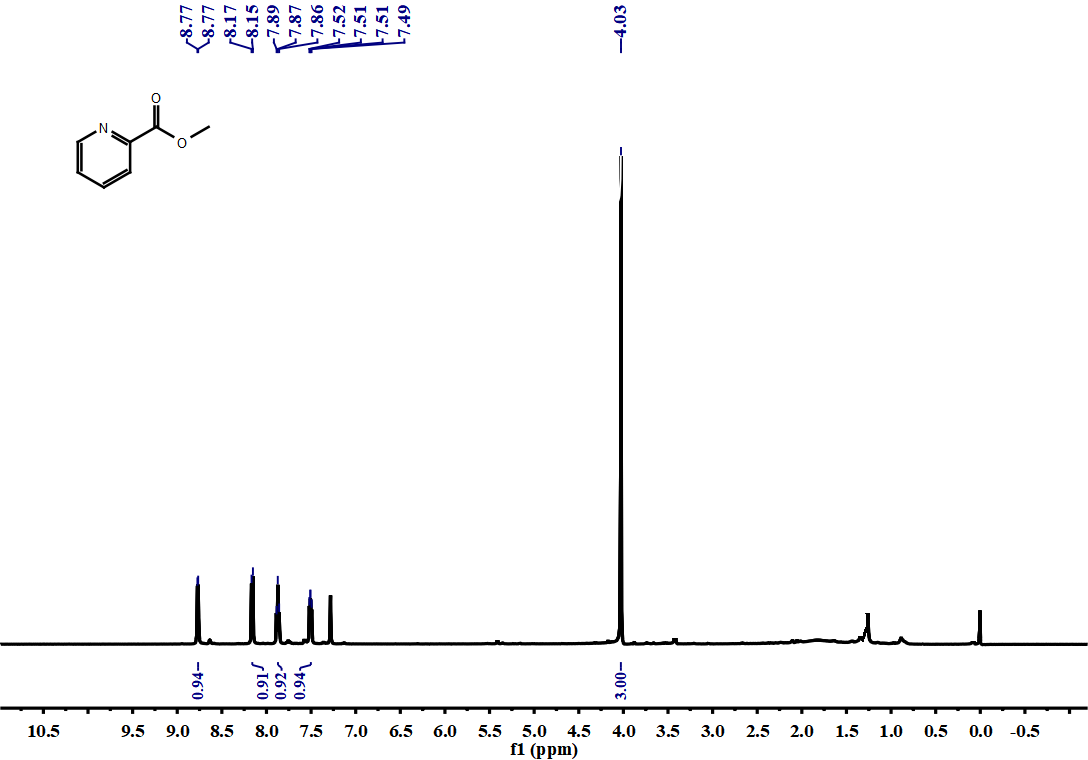


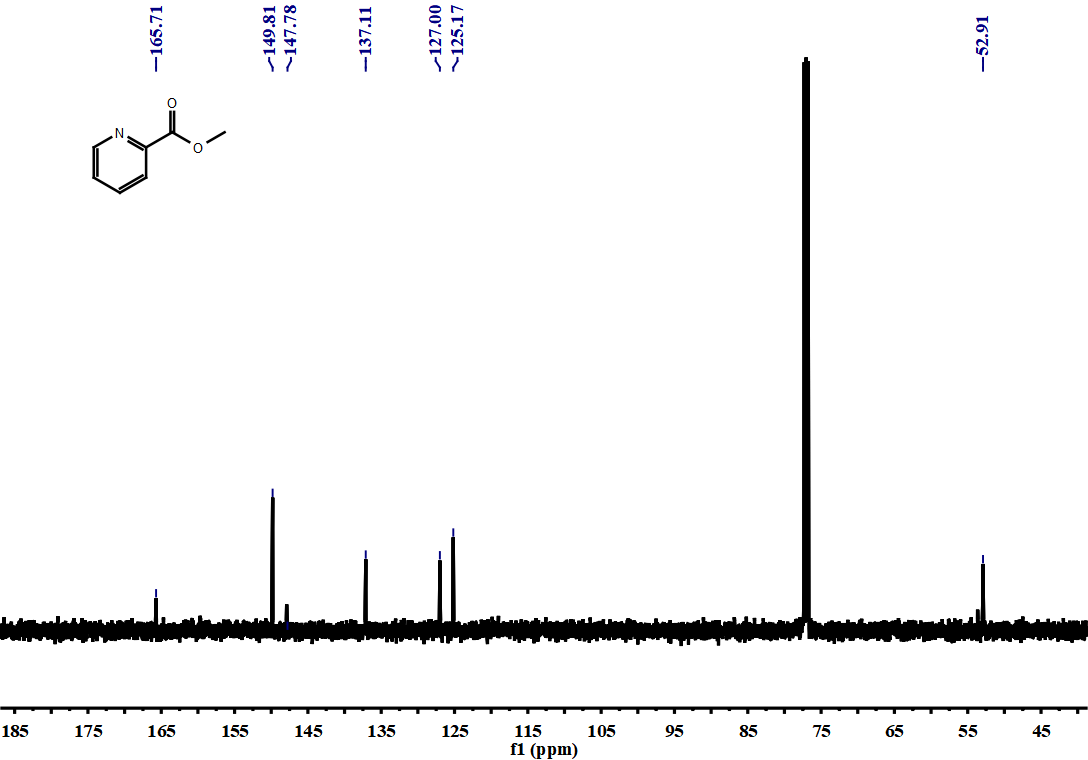


**Figure S29.** ^1^H NMR (top) and ^13^C NMR (bottom) of **20** (CDCl_3_ as the solvent).


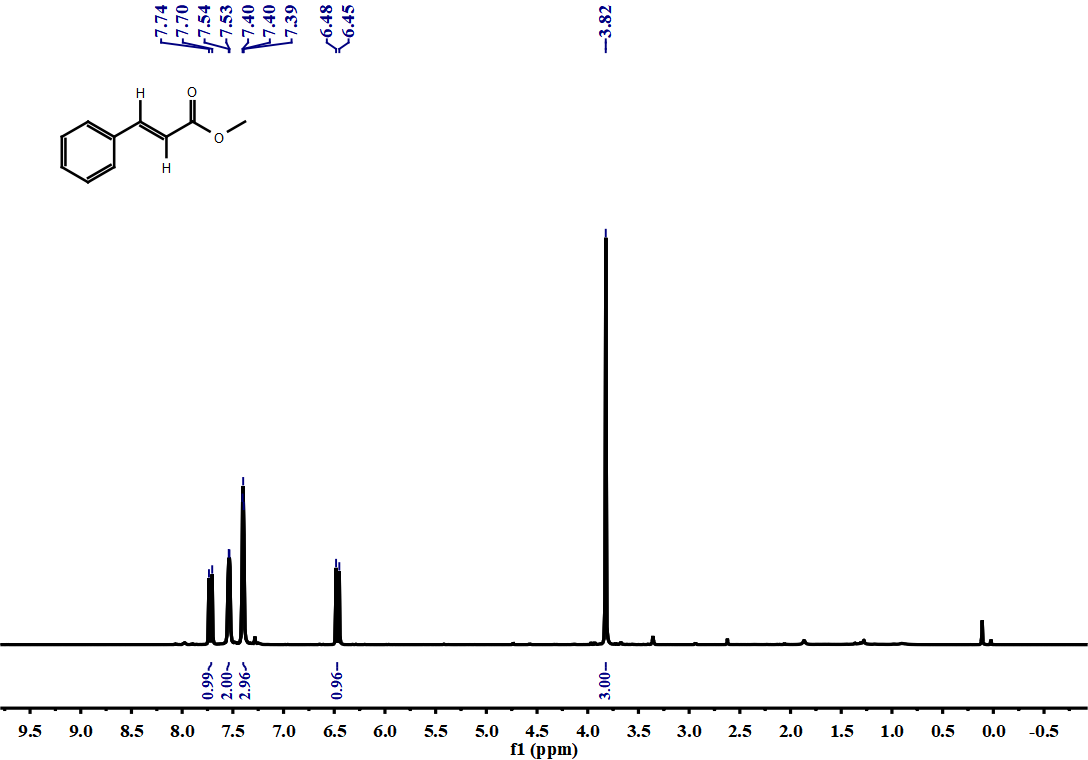


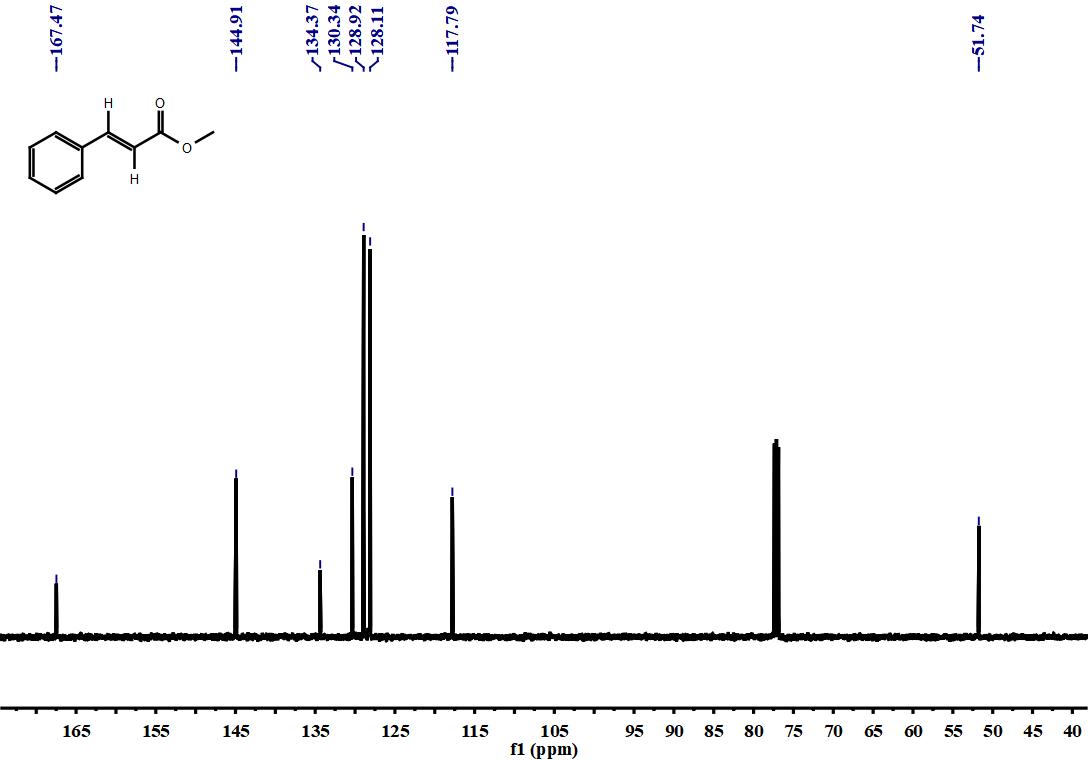


**Figure S30.** ^1^H NMR (top) and ^13^C NMR (bottom) of **21** (CDCl_3_ as the solvent).


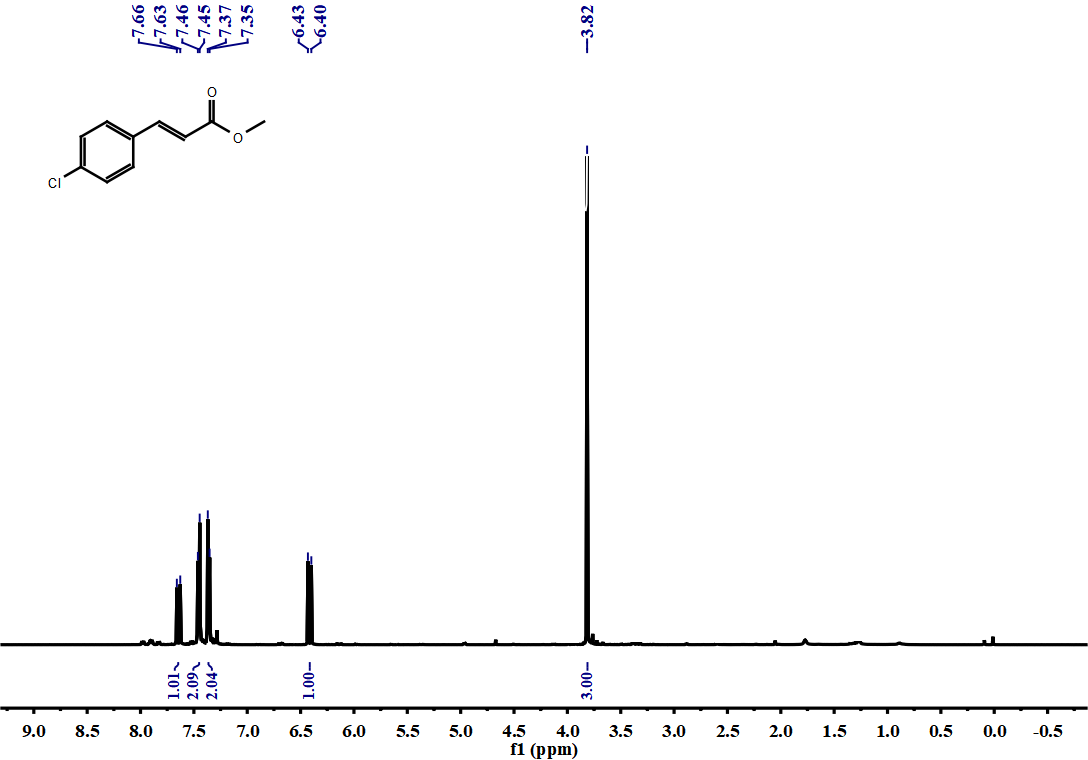


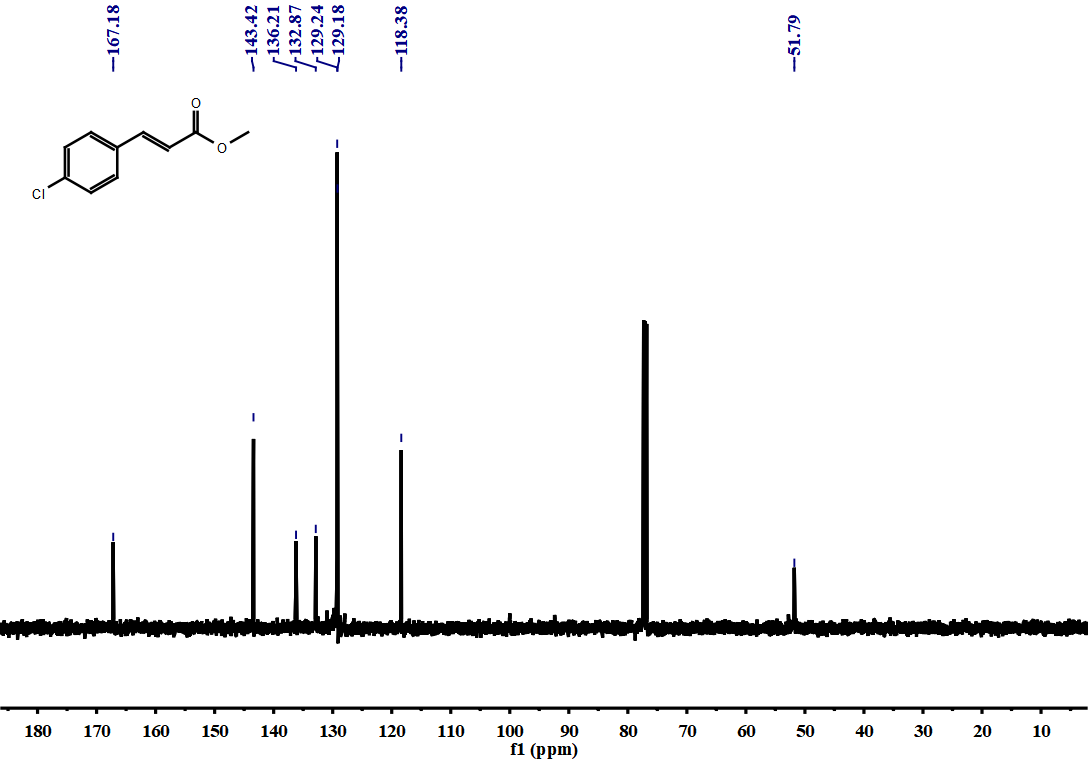


**Figure S31.** ^1^H NMR (top) and ^13^C NMR (bottom) of **22** (CDCl_3_ as the solvent).


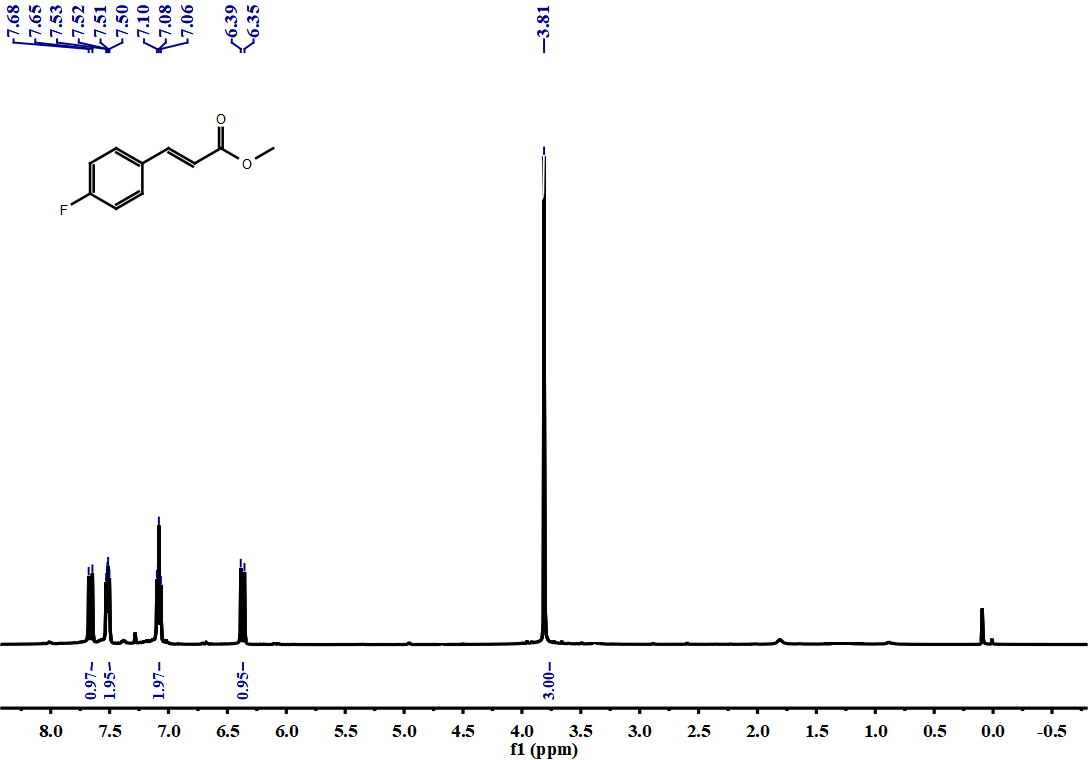


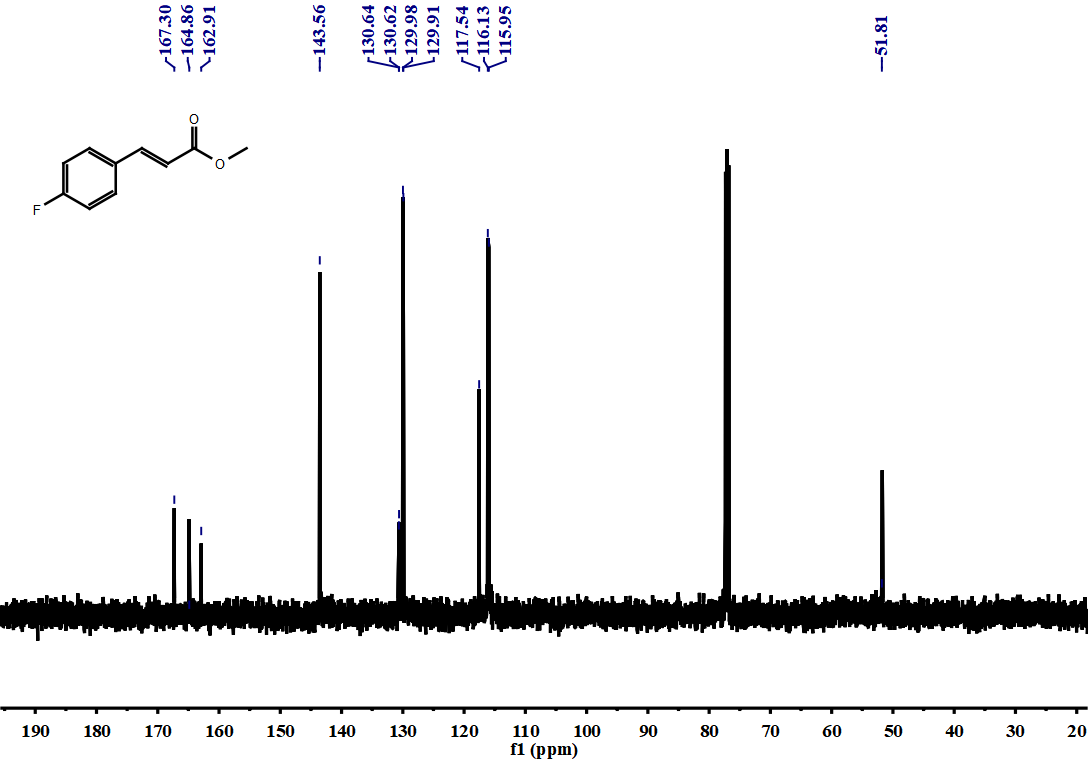


**Figure S32.** ^1^H NMR (top) and ^13^C NMR (bottom) of **23** (CDCl_3_ as the solvent).

**
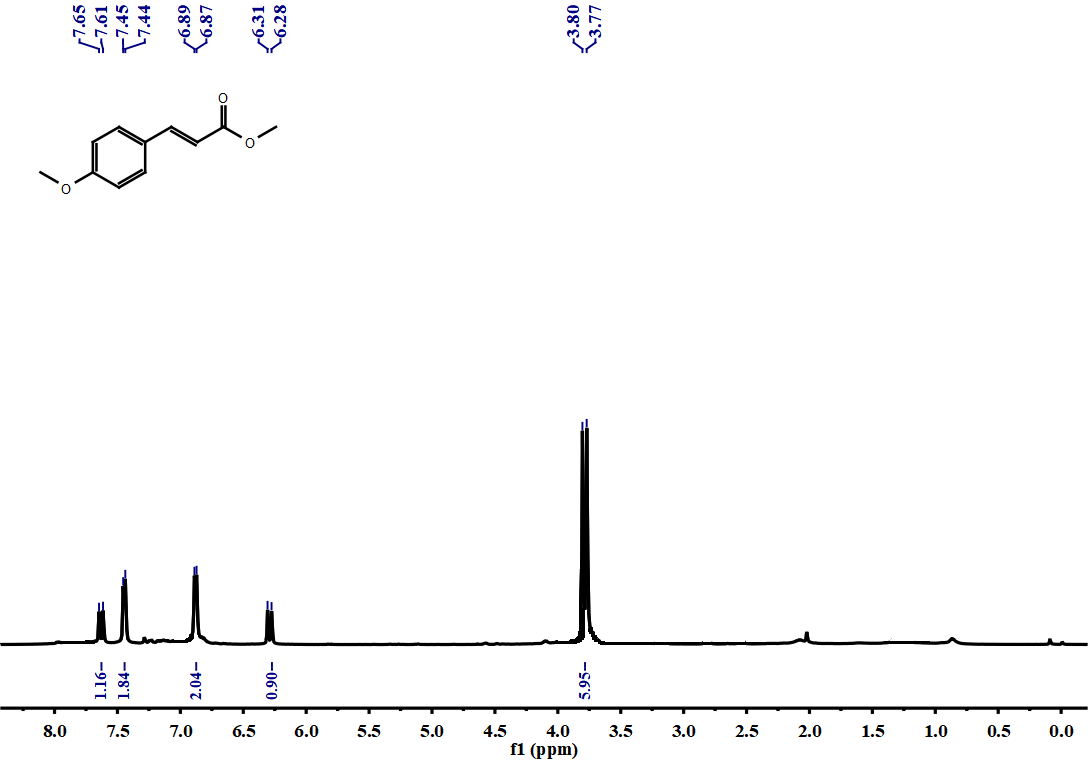
**


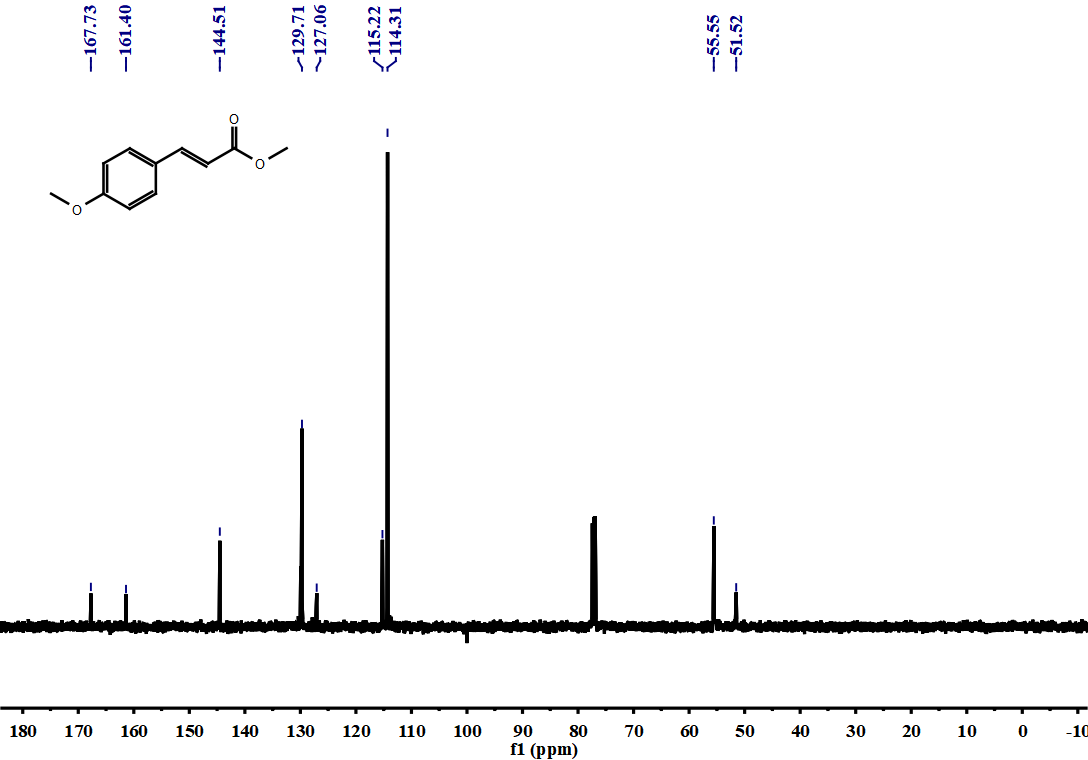


**Figure S33.** ^1^H NMR (top) and ^13^C NMR (bottom) of **24** (CDCl_3_ as the solvent).


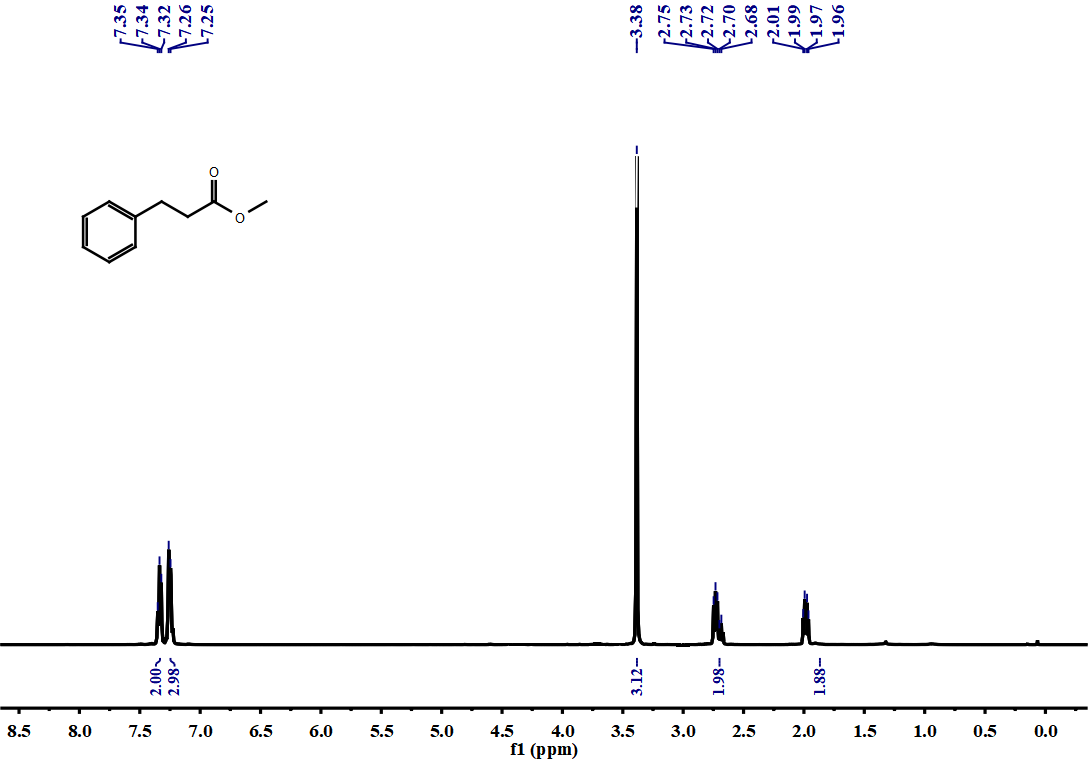


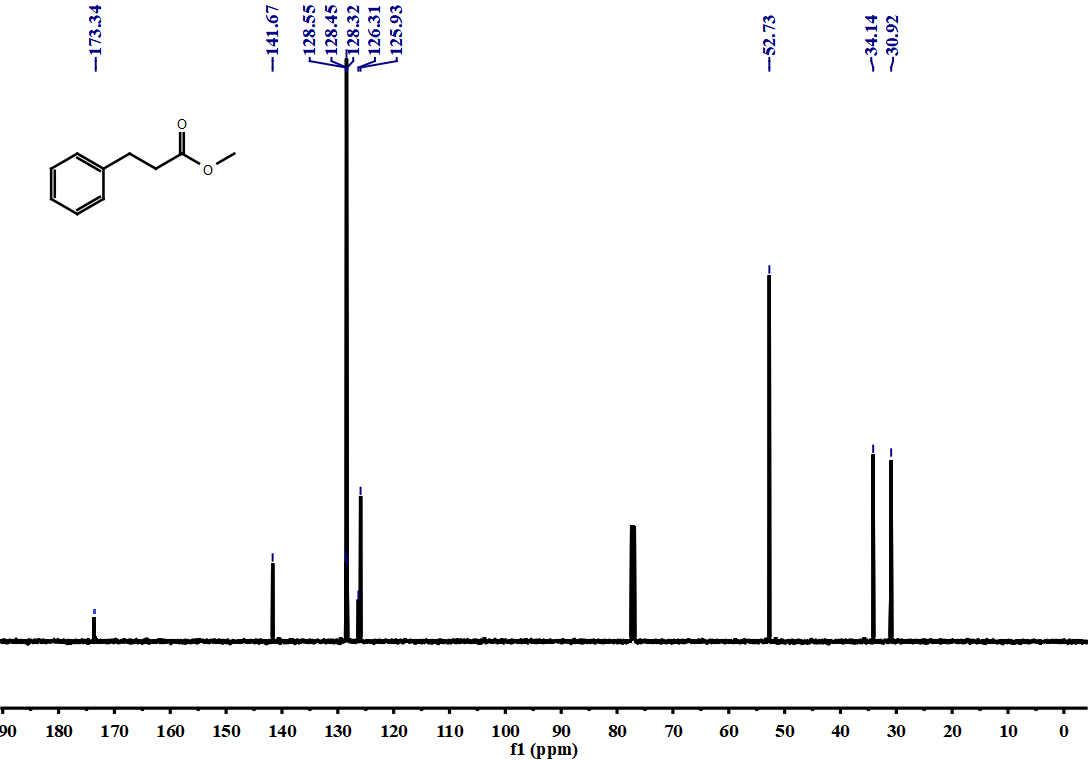


**Figure S34.** ^1^H NMR (top) and ^13^C NMR (bottom) of **25** (CDCl_3_ as the solvent).


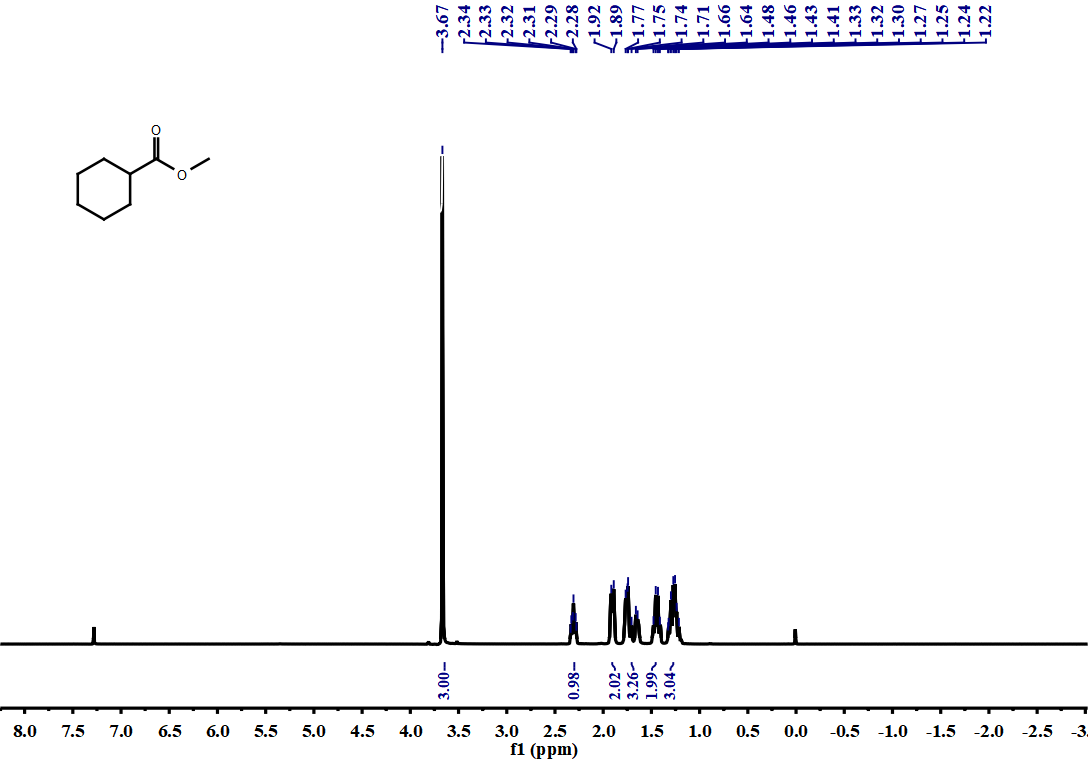


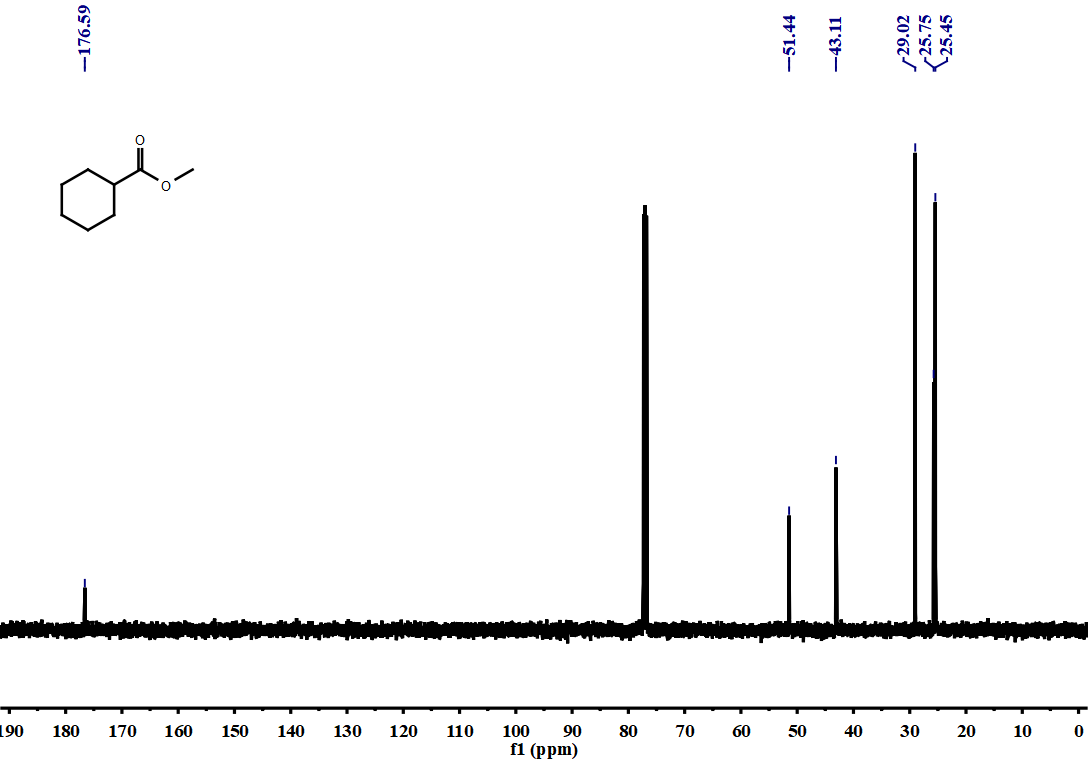


**Figure S35.** ^1^H NMR (top) and ^13^C NMR (bottom) of **26** (CDCl_3_ as the solvent).

**
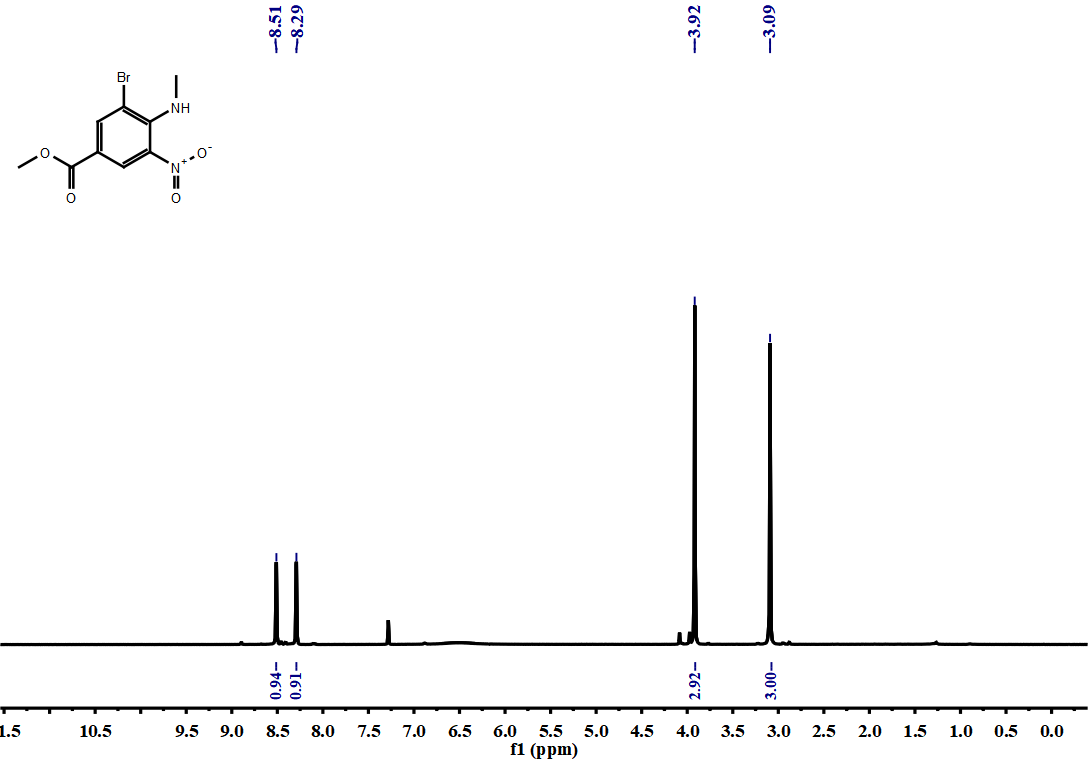
**

**
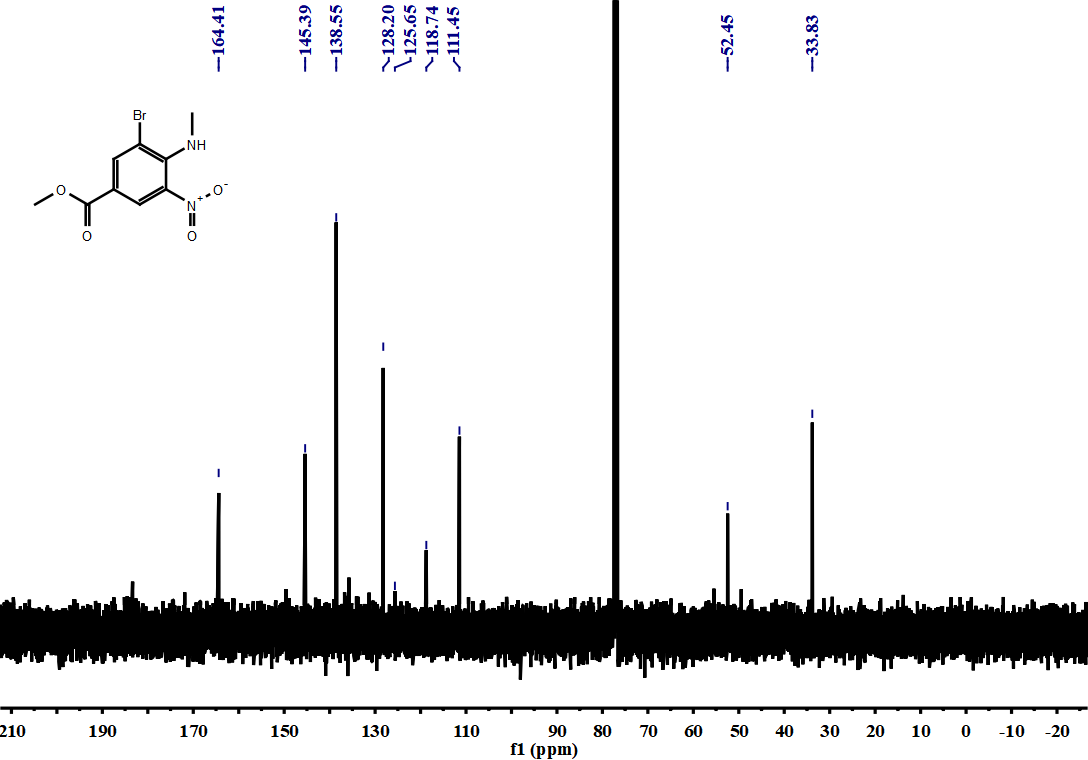
**

**Figure S36.** ^1^H NMR (top) and ^13^C NMR (bottom) of **31** (CDCl_3_ as the solvent).

**
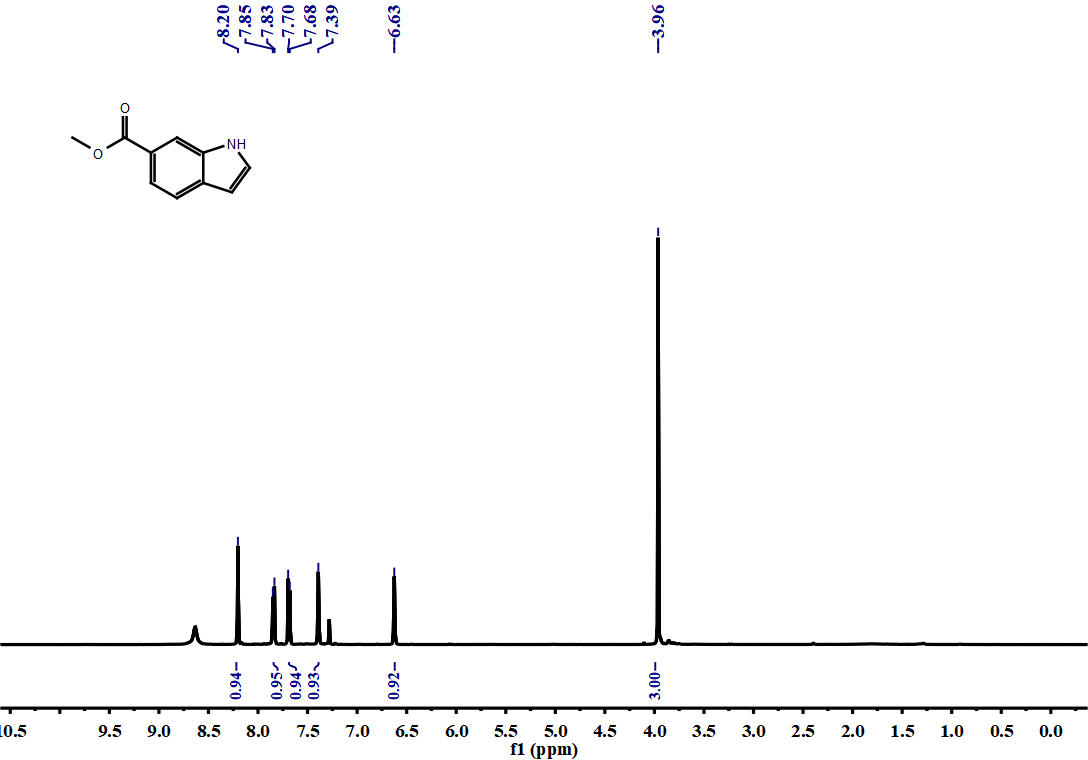
**

**
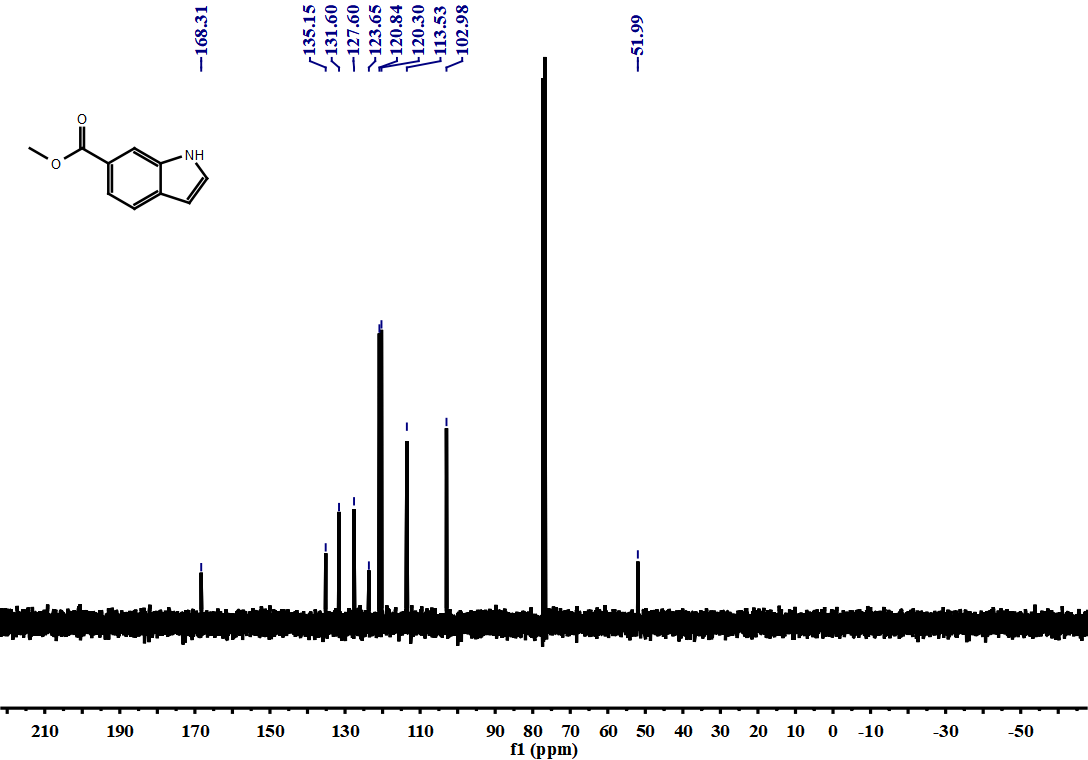
**

**Figure S37.** ^1^H NMR (top) and ^13^C NMR (bottom) of **32** (CDCl_3_ as the solvent).

**
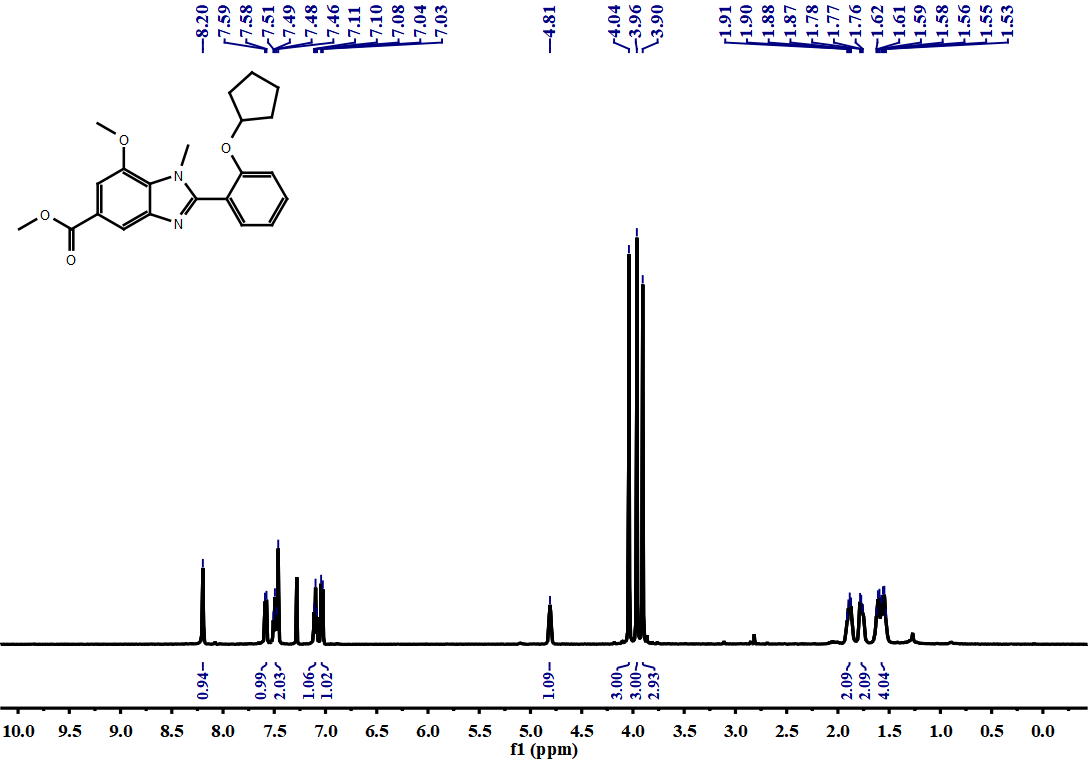
**

**
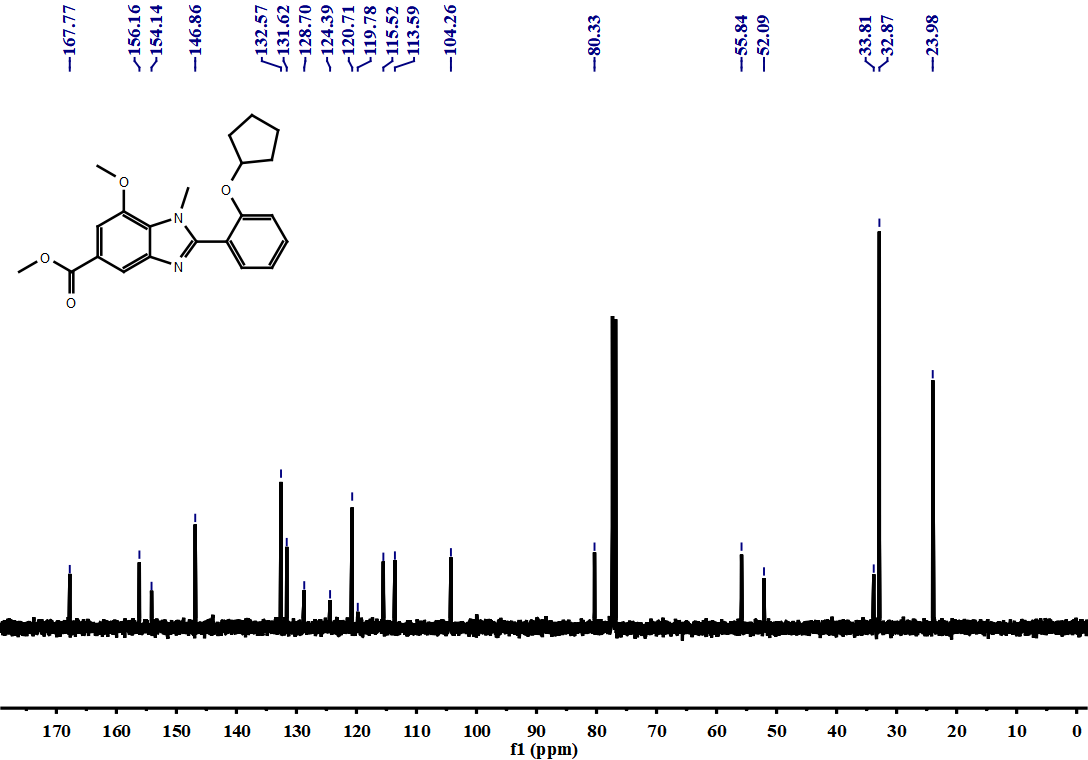
**

**Figure S38.** ^1^H NMR (top) and ^13^C NMR (bottom) of **33** (CDCl_3_ as the solvent).

**
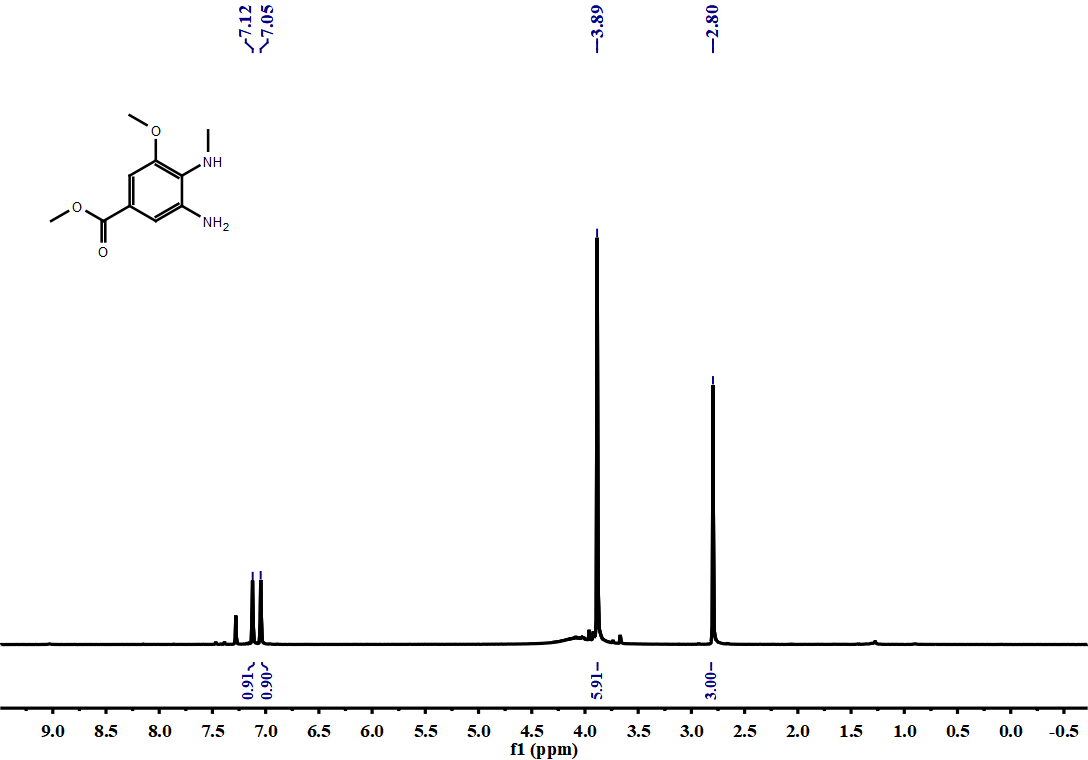
**

**
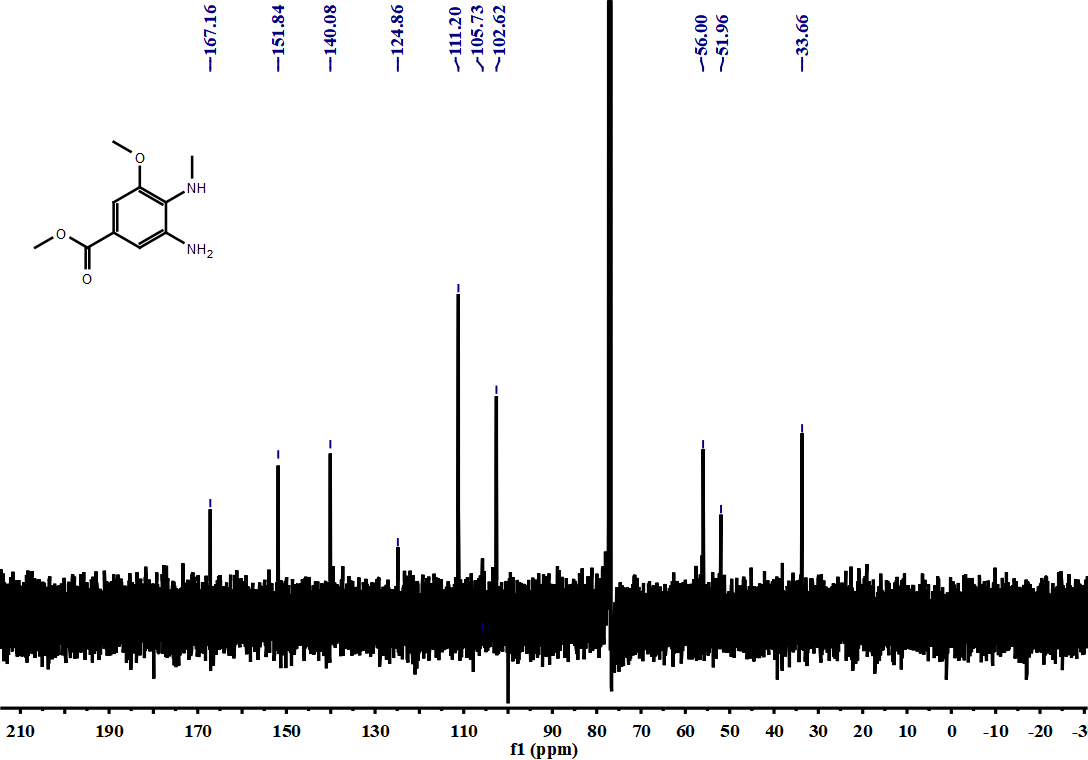
**

**Figure S39.** ^1^H NMR (top) and ^13^C NMR (bottom) of **34** (CDCl_3_ as the solvent).


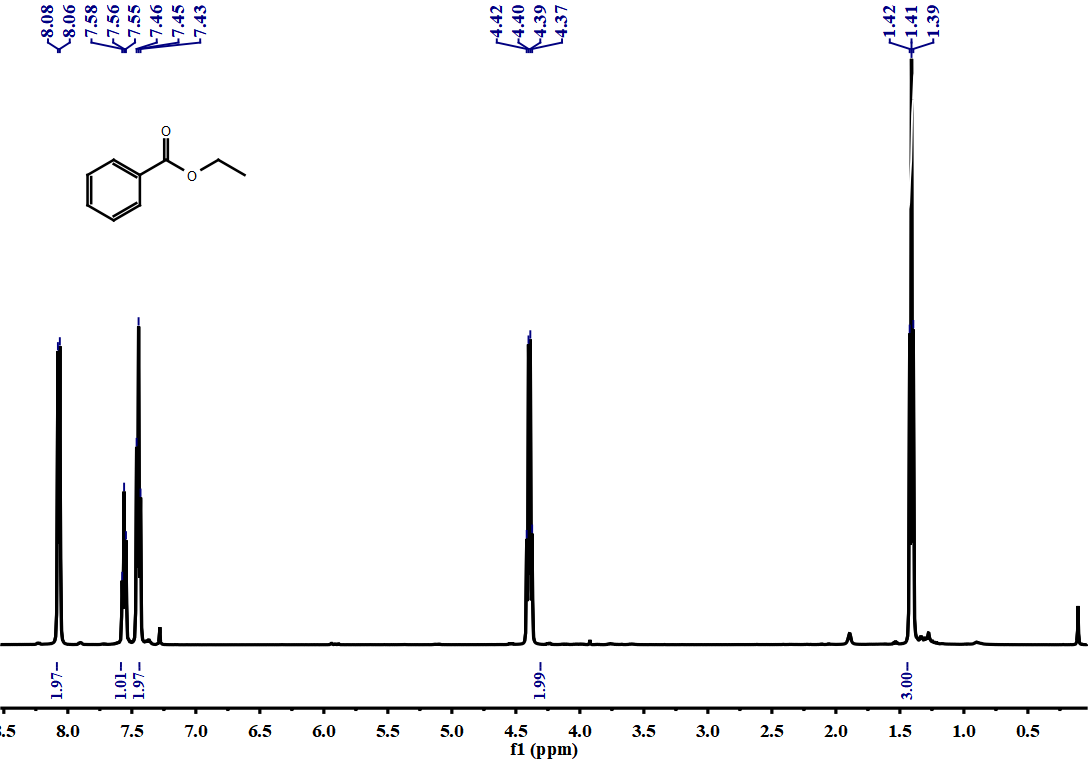


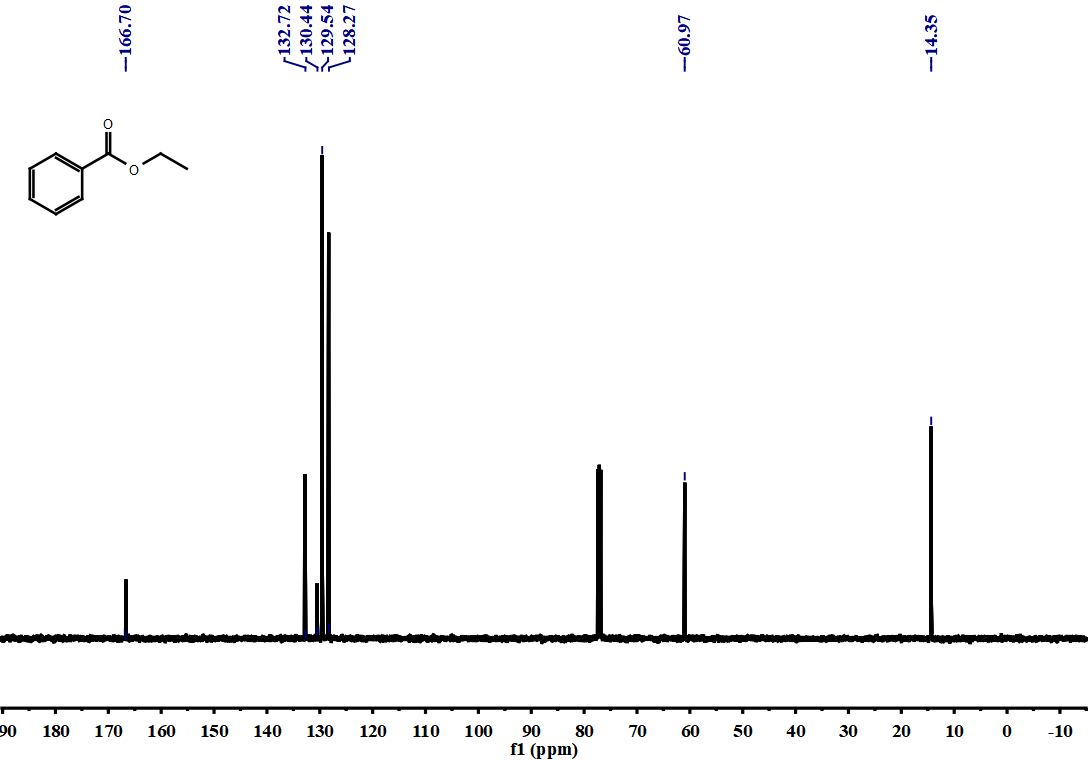


**Figure S40.** ^1^H NMR (top) and ^13^C NMR (bottom) of **35** (CDCl_3_ as the solvent).


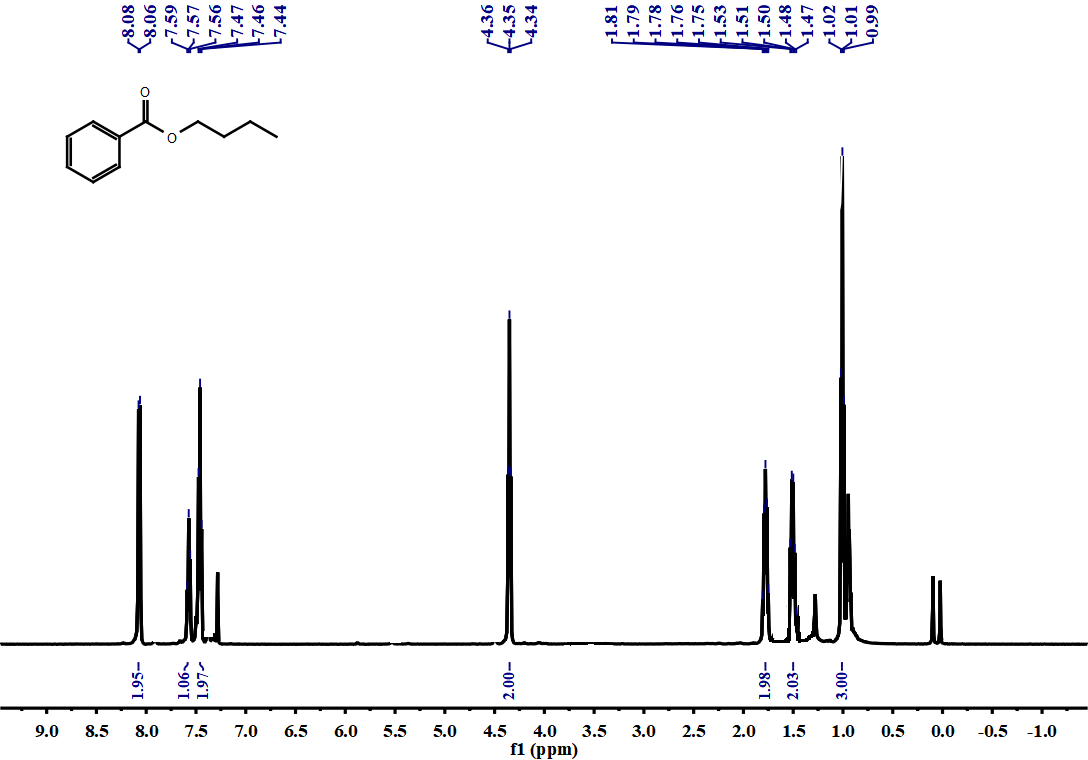


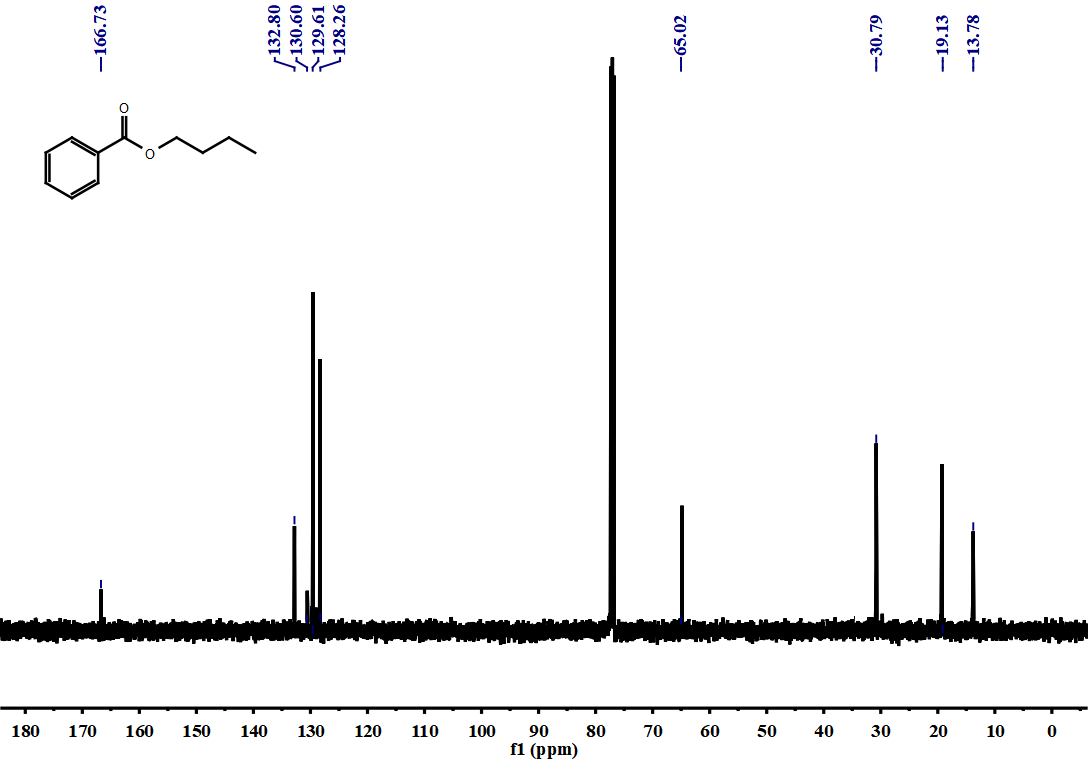


**Figure S41.** ^1^H NMR (top) and ^13^C NMR (bottom) of **36** (CDCl_3_ as the solvent).


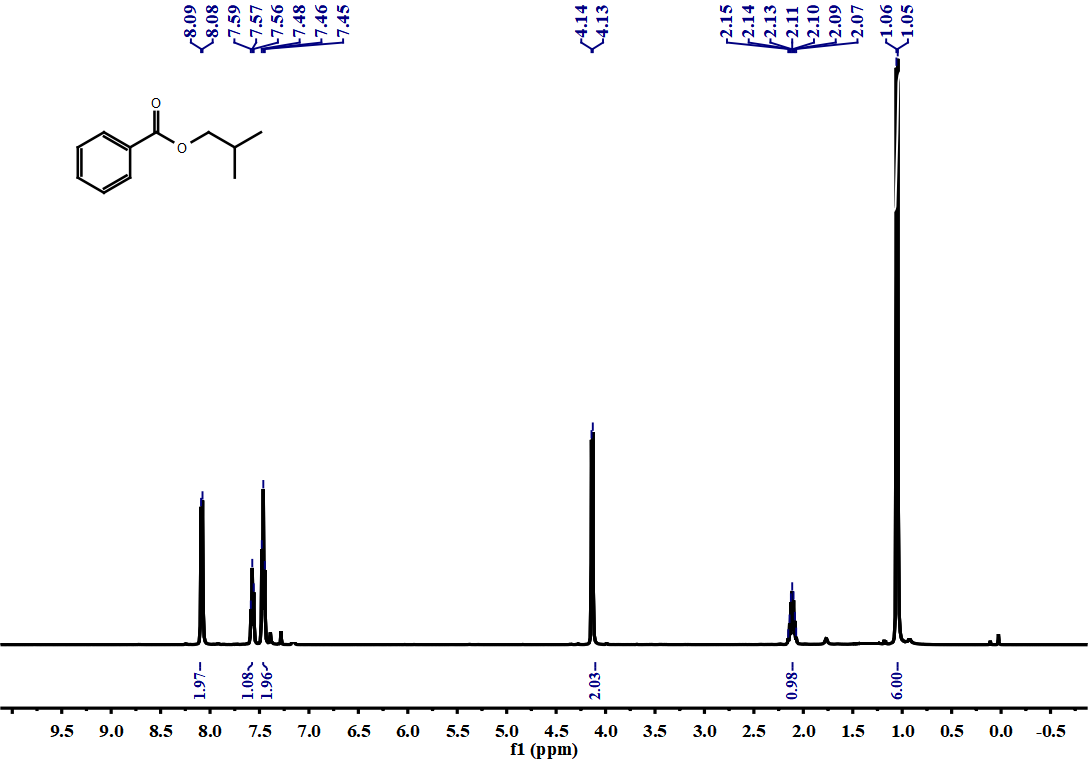


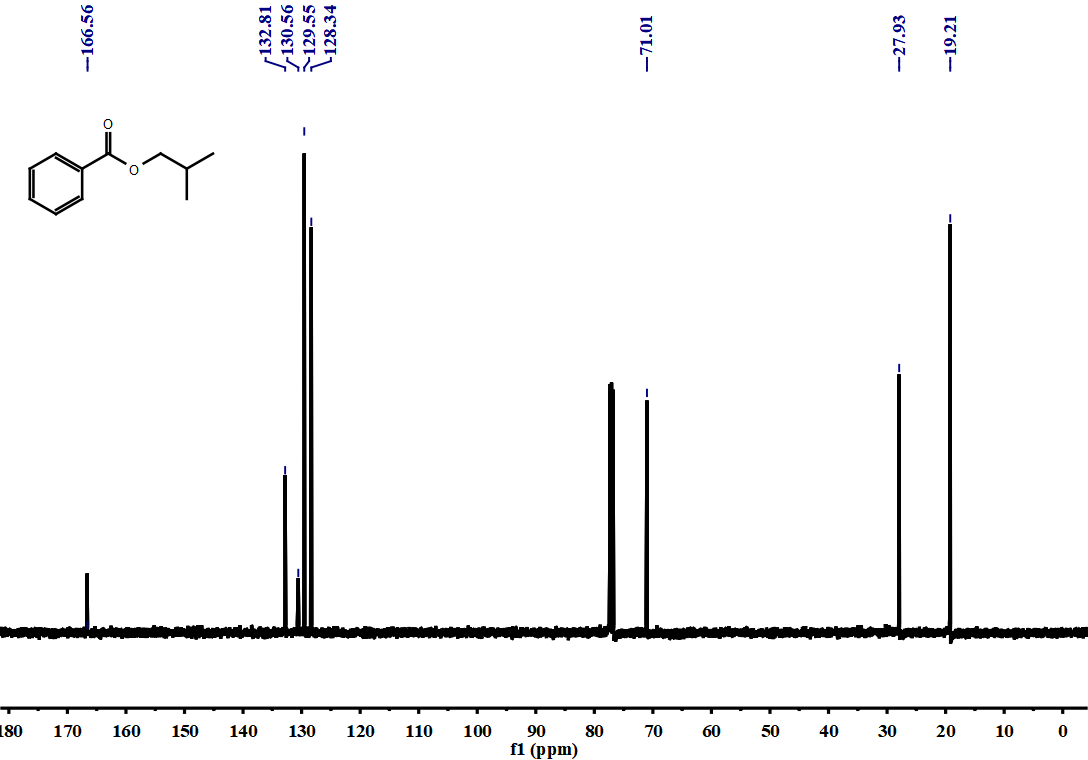


**Figure S42.** ^1^H NMR (top) and ^13^C NMR (bottom) of **37** (CDCl_3_ as the solvent).


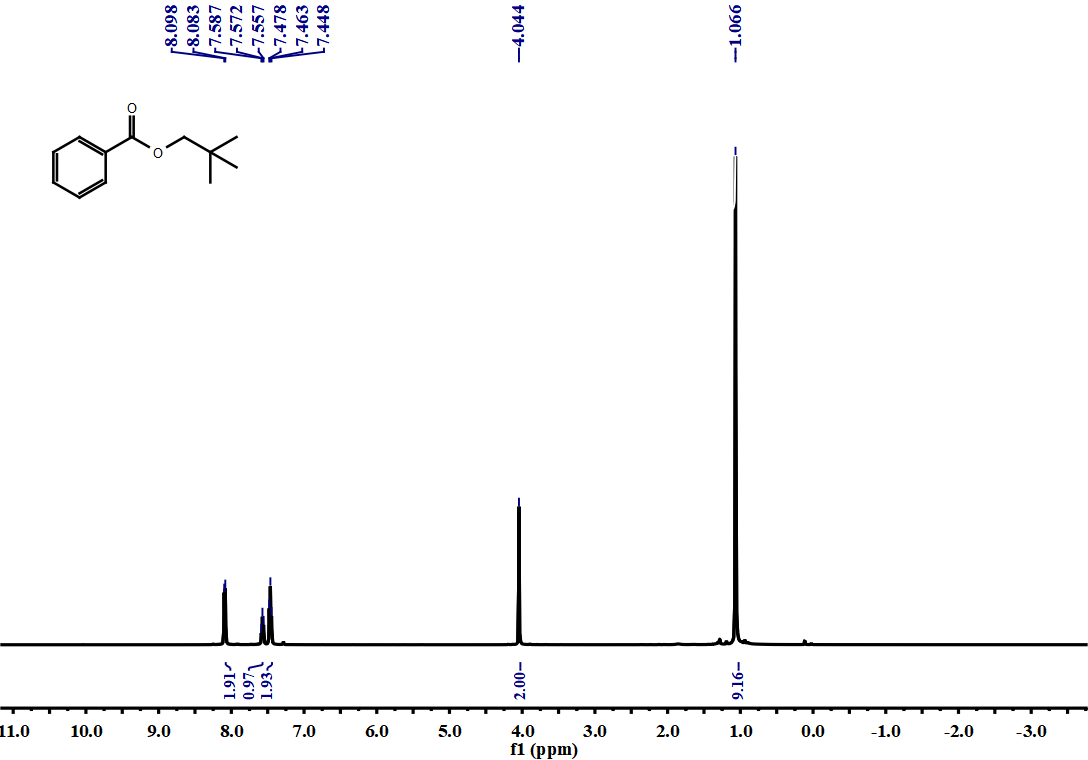


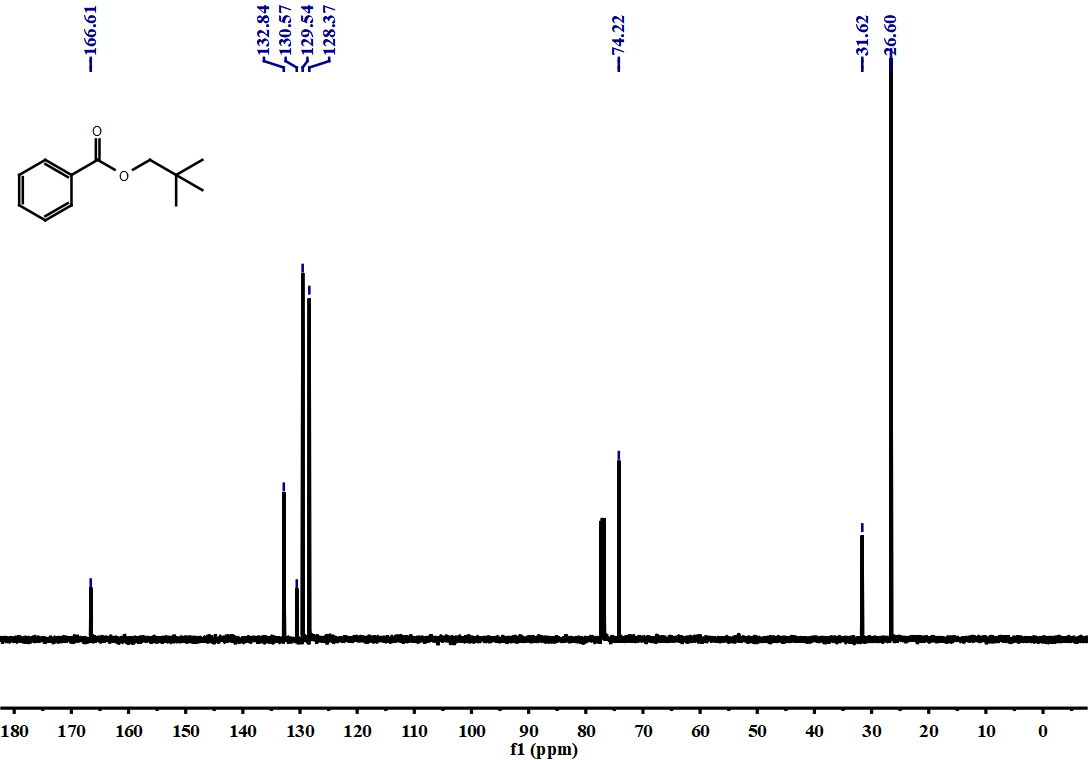


**Figure S43.** ^1^H NMR (top) and ^13^C NMR (bottom) of **38** (CDCl_3_ as the solvent).


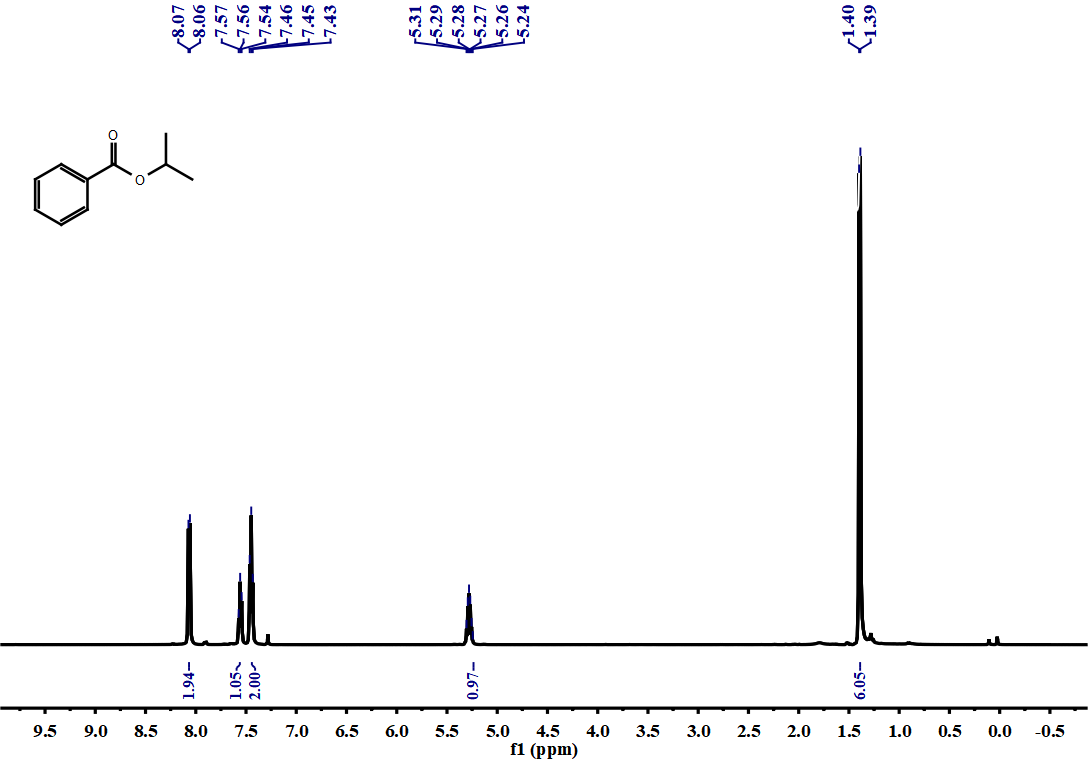


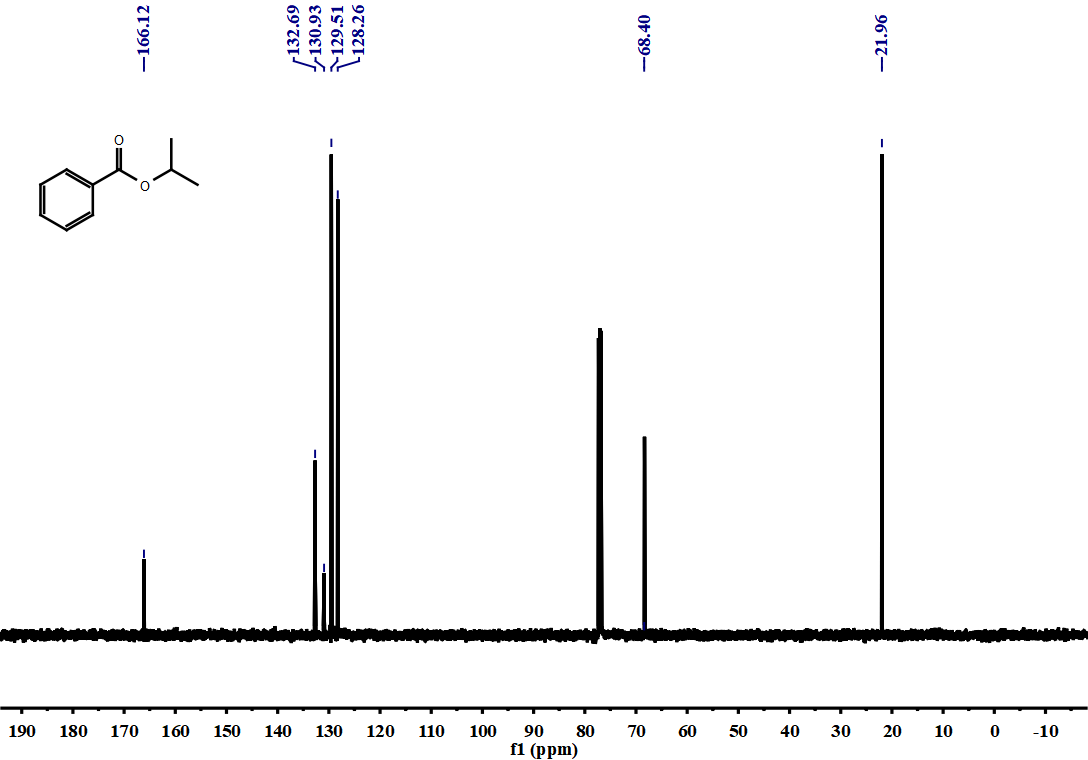


**Figure S44.** ^1^H NMR (top) and ^13^C NMR (bottom) of **39** (CDCl_3_ as the solvent).


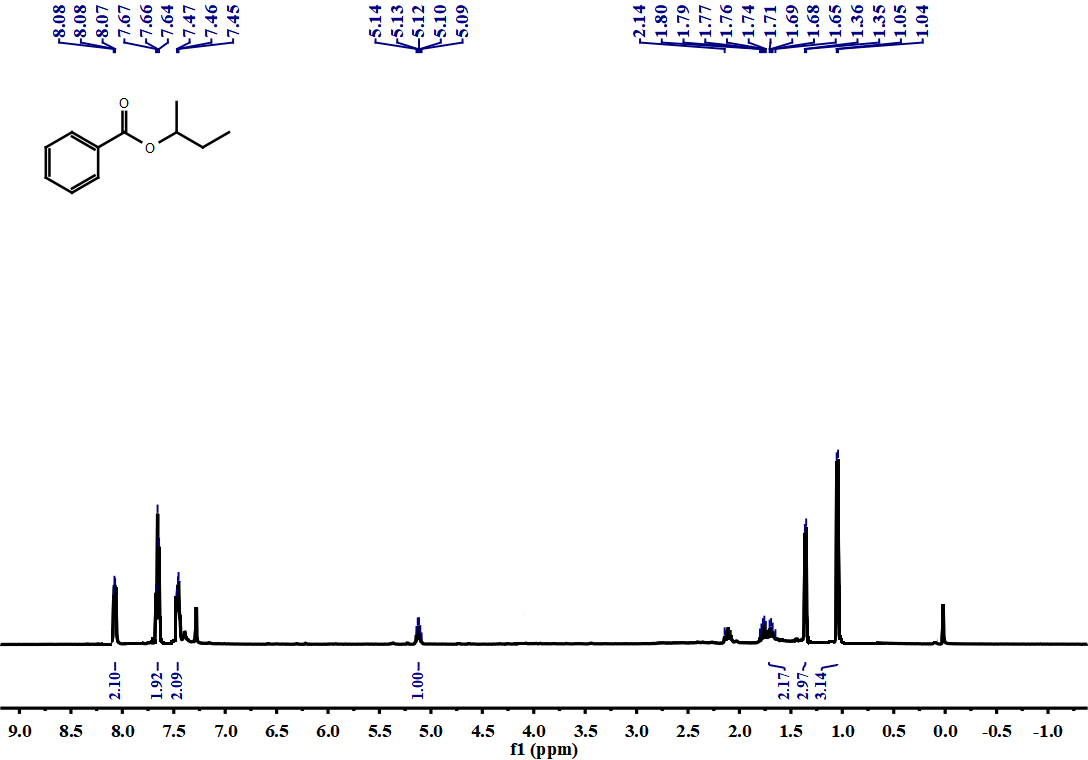


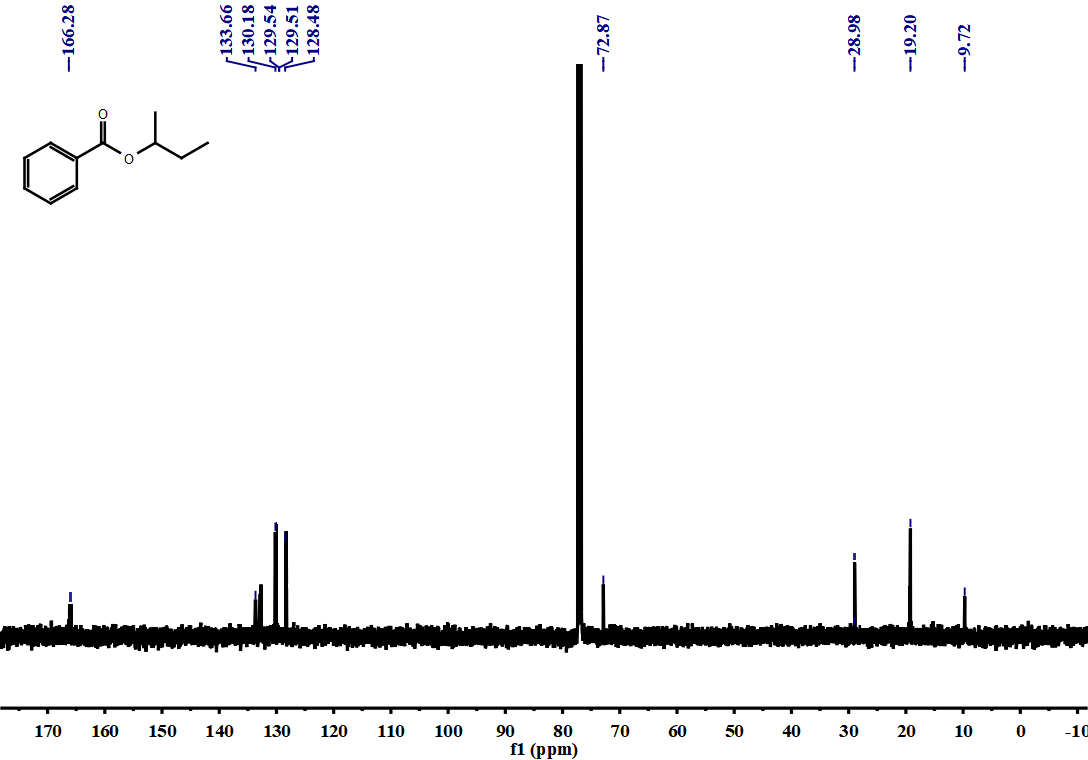


**Figure S45.** ^1^H NMR (top) and ^13^C NMR (bottom) of **40** (CDCl_3_ as the solvent).


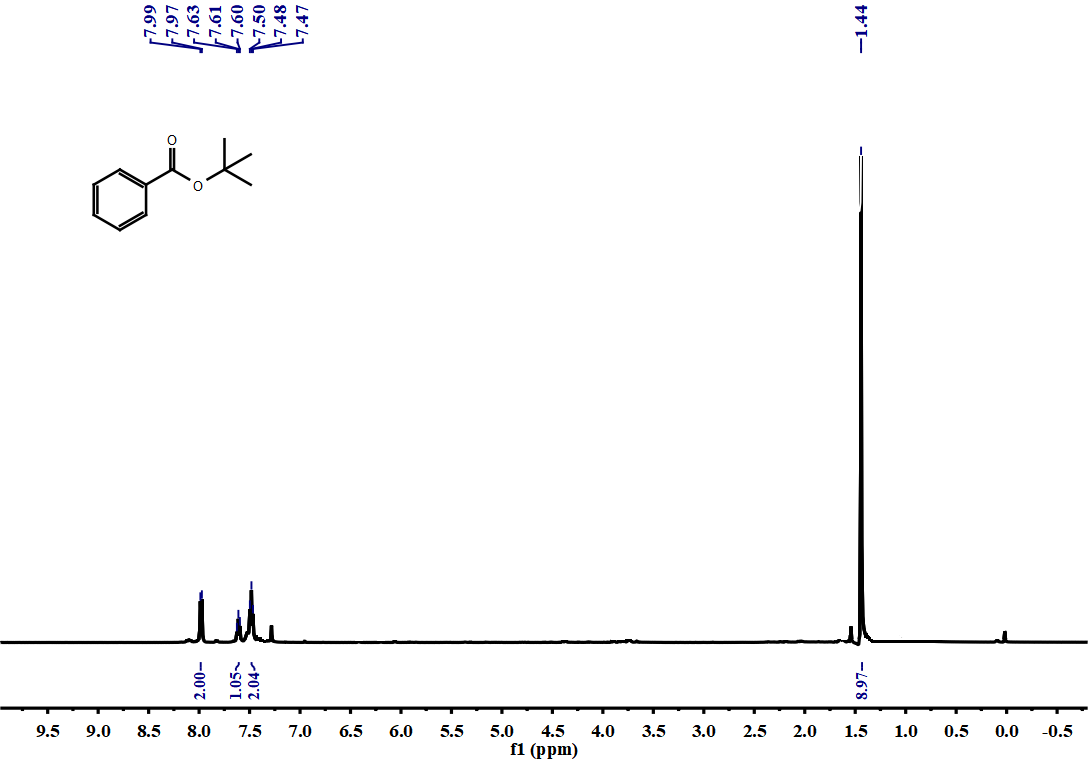


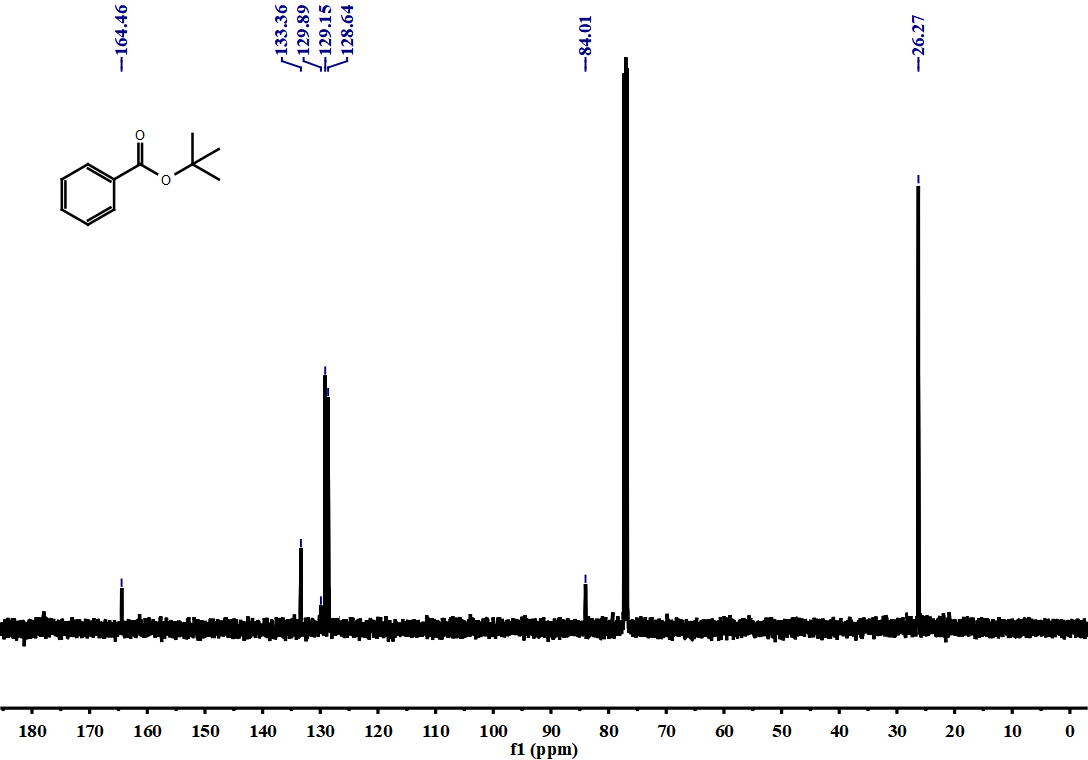


**Figure S46.** ^1^H NMR (top) and ^13^C NMR (bottom) of **41** (CDCl_3_ as the solvent).


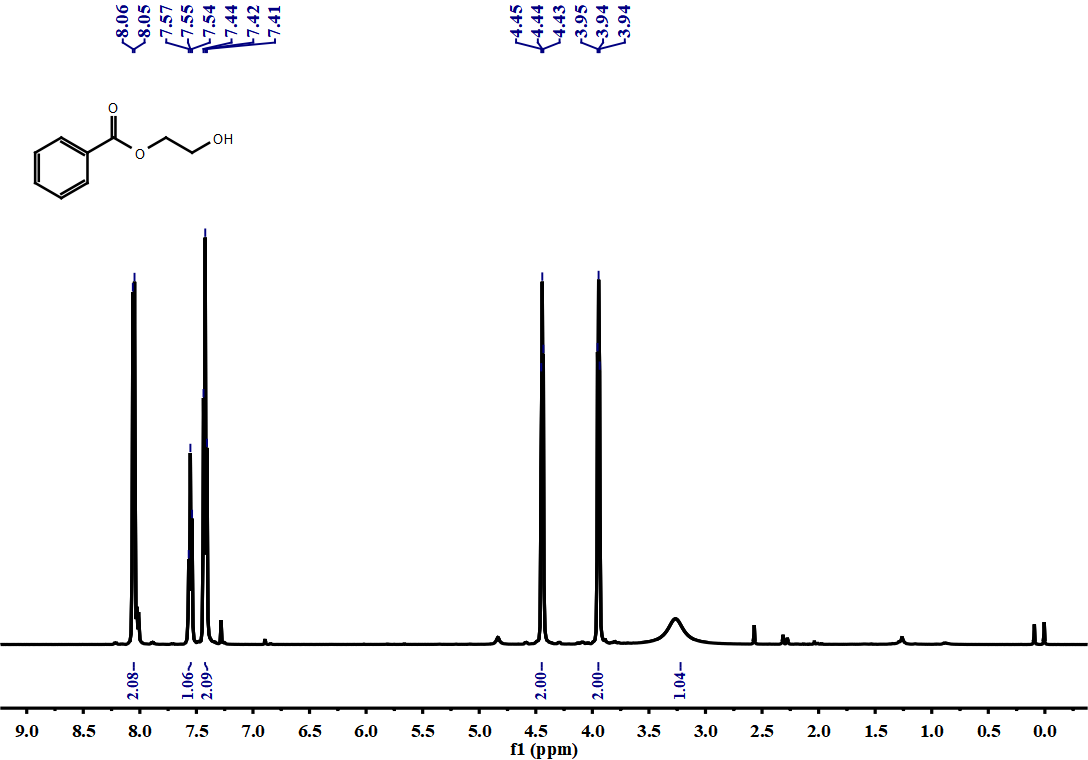


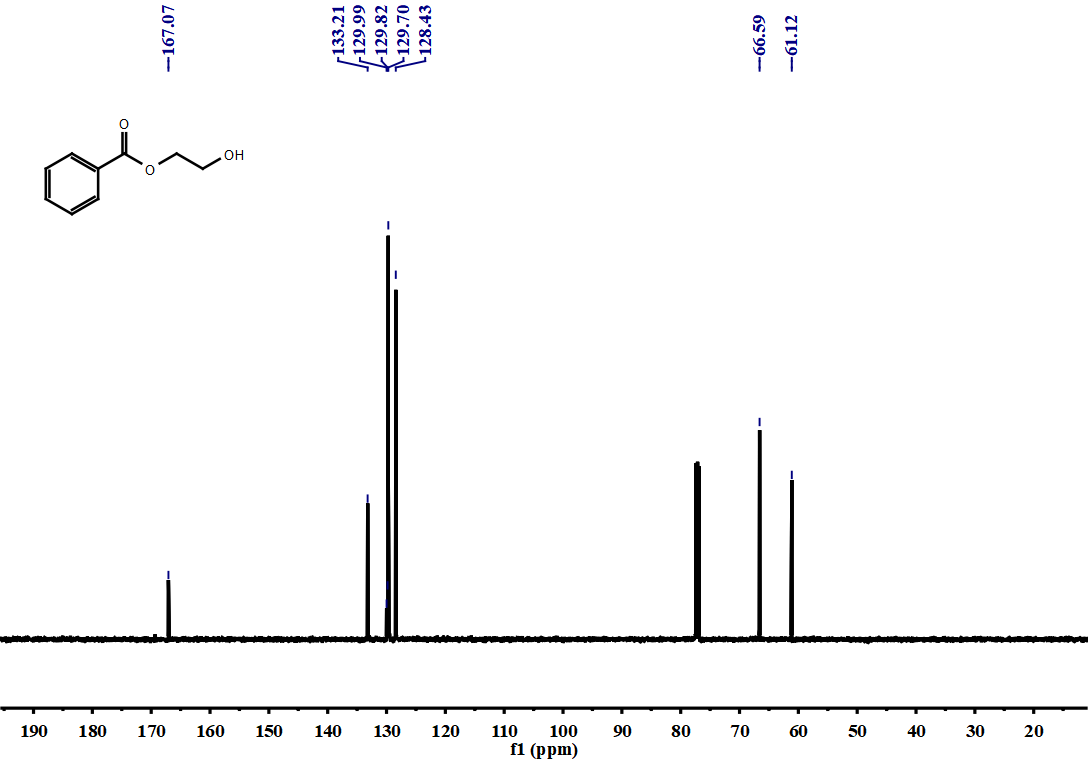


**Figure S47.** ^1^H NMR (top) and ^13^C NMR (bottom) of **42** (CDCl_3_ as the solvent).


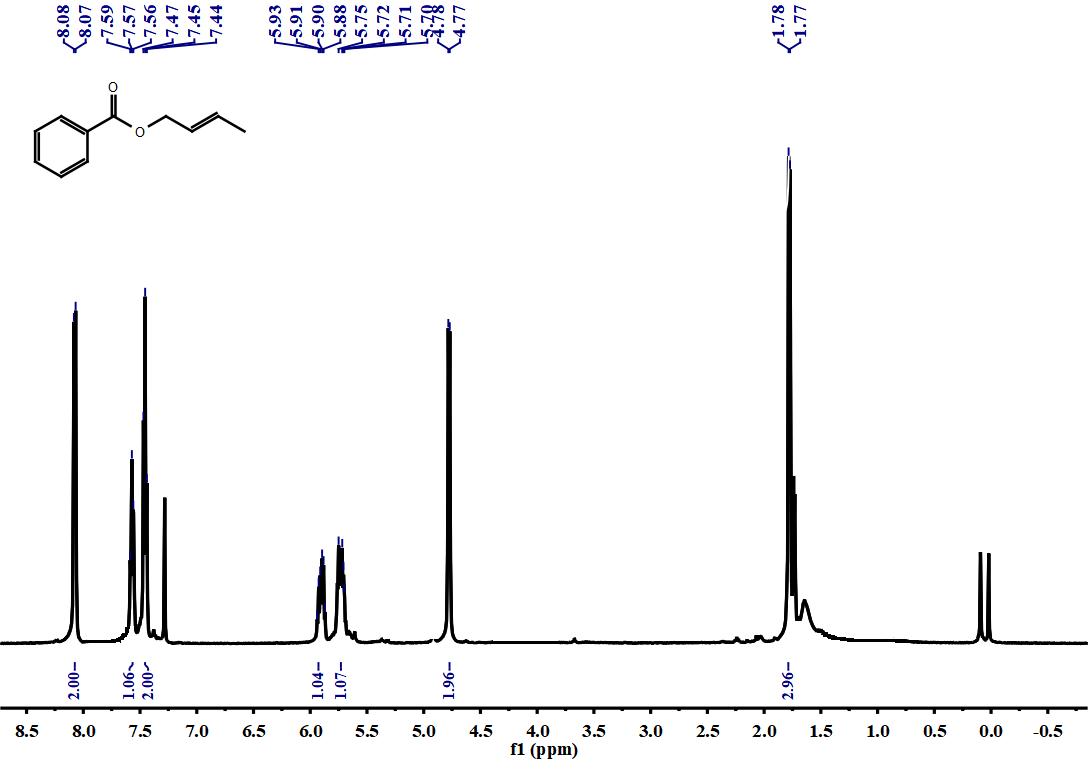


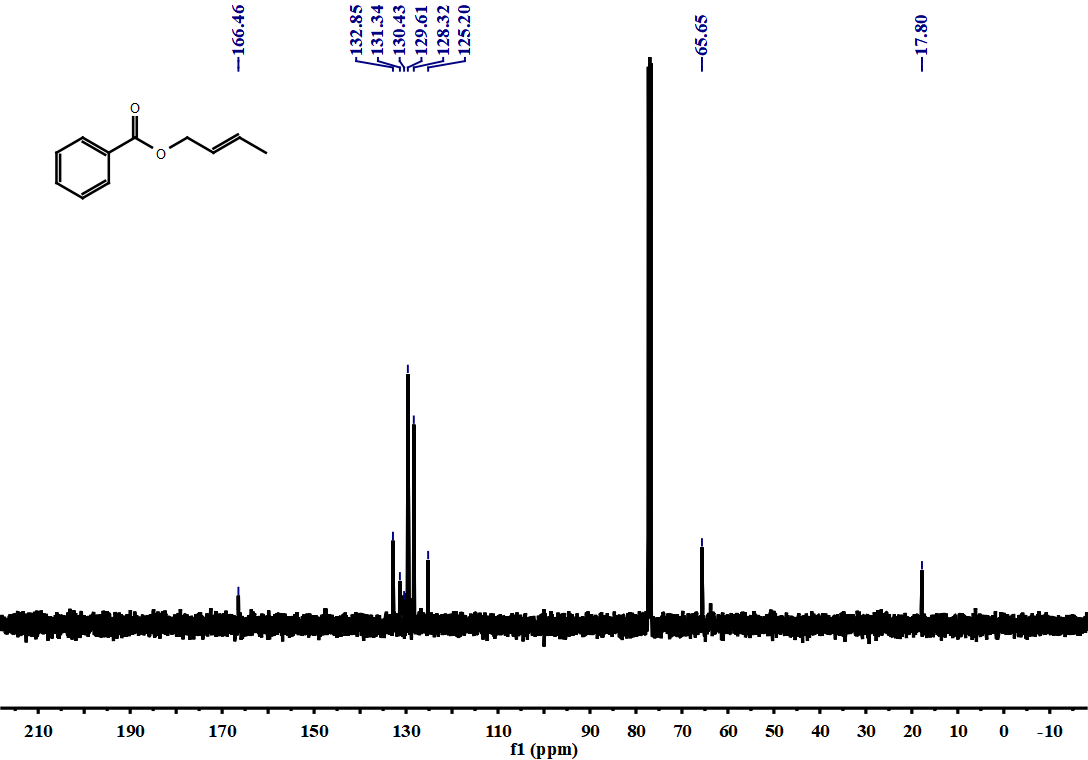


**Figure S48.** ^1^H NMR (top) and ^13^C NMR (bottom) of **43** (CDCl_3_ as the solvent).


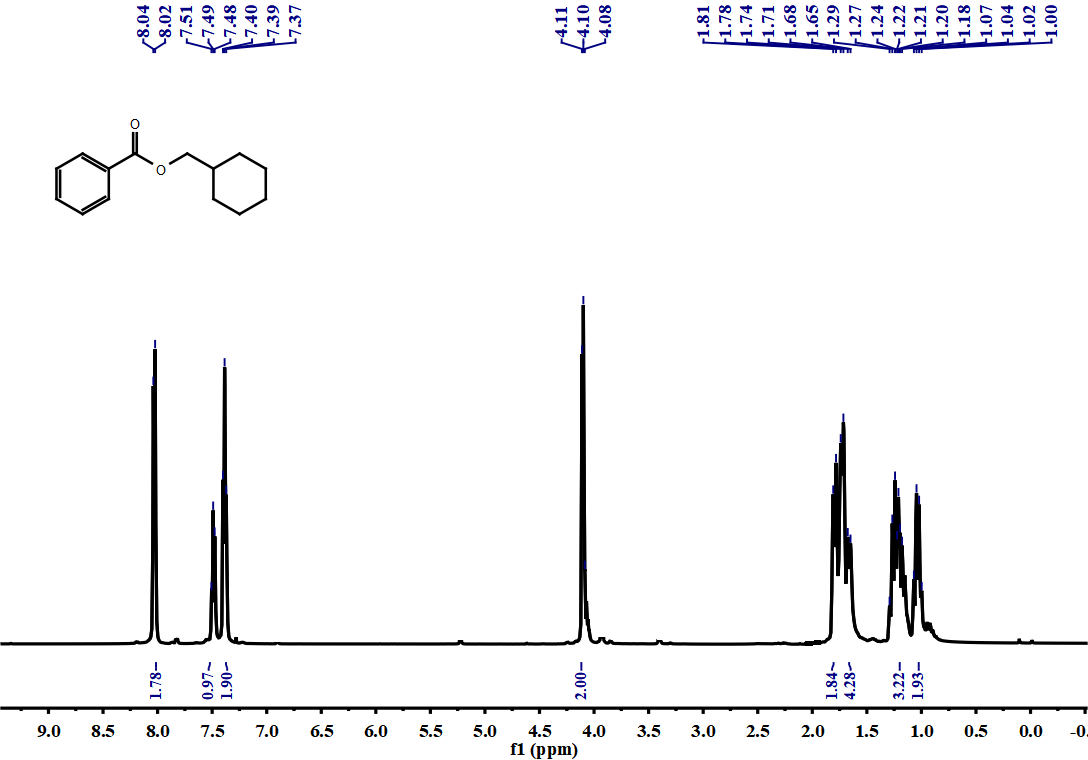


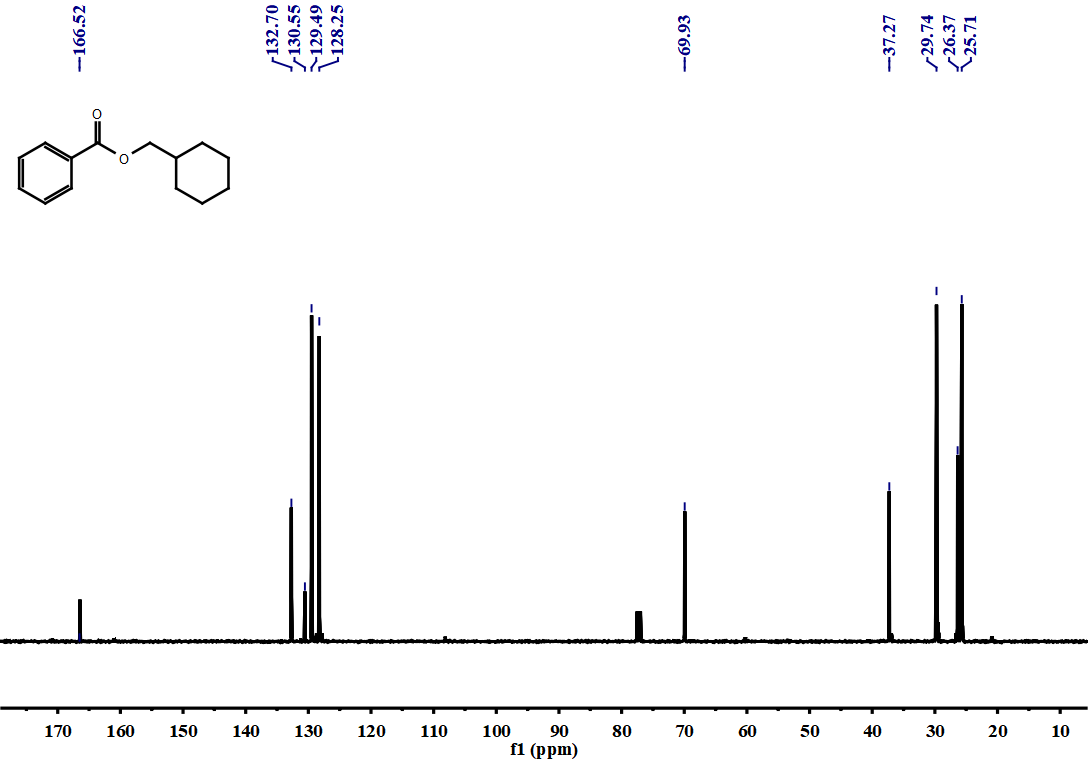


**Figure S49.** ^1^H NMR (top) and ^13^C NMR (bottom) of **44** (CDCl_3_ as the solvent).


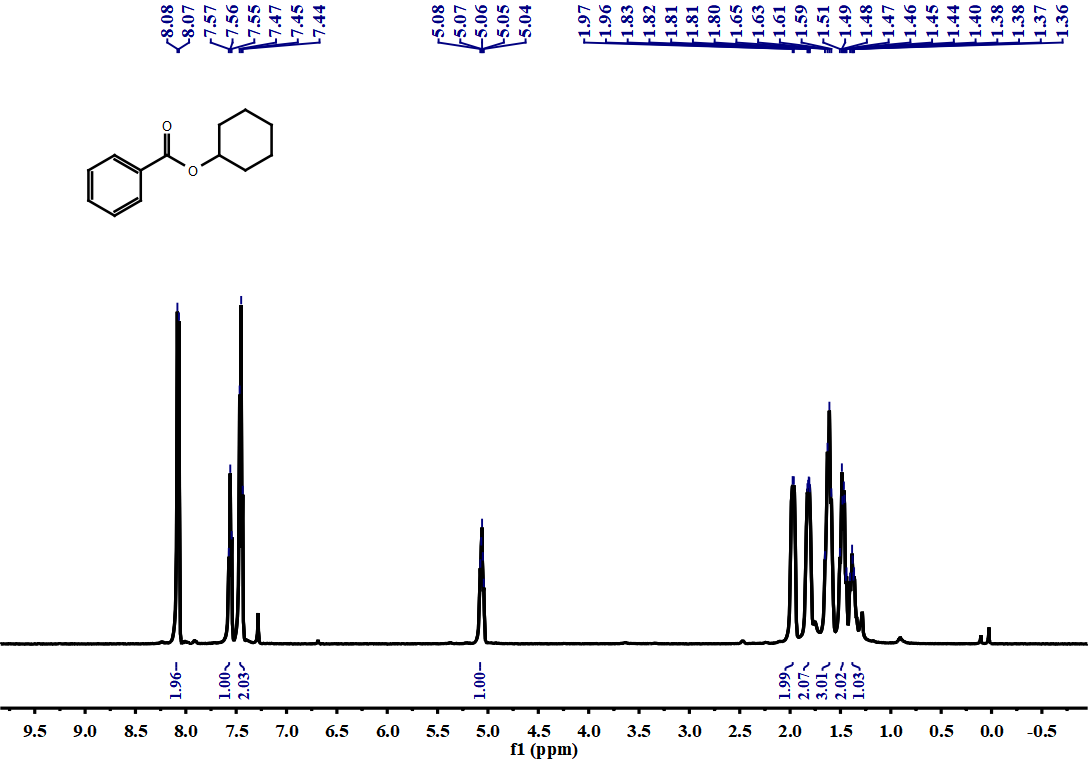


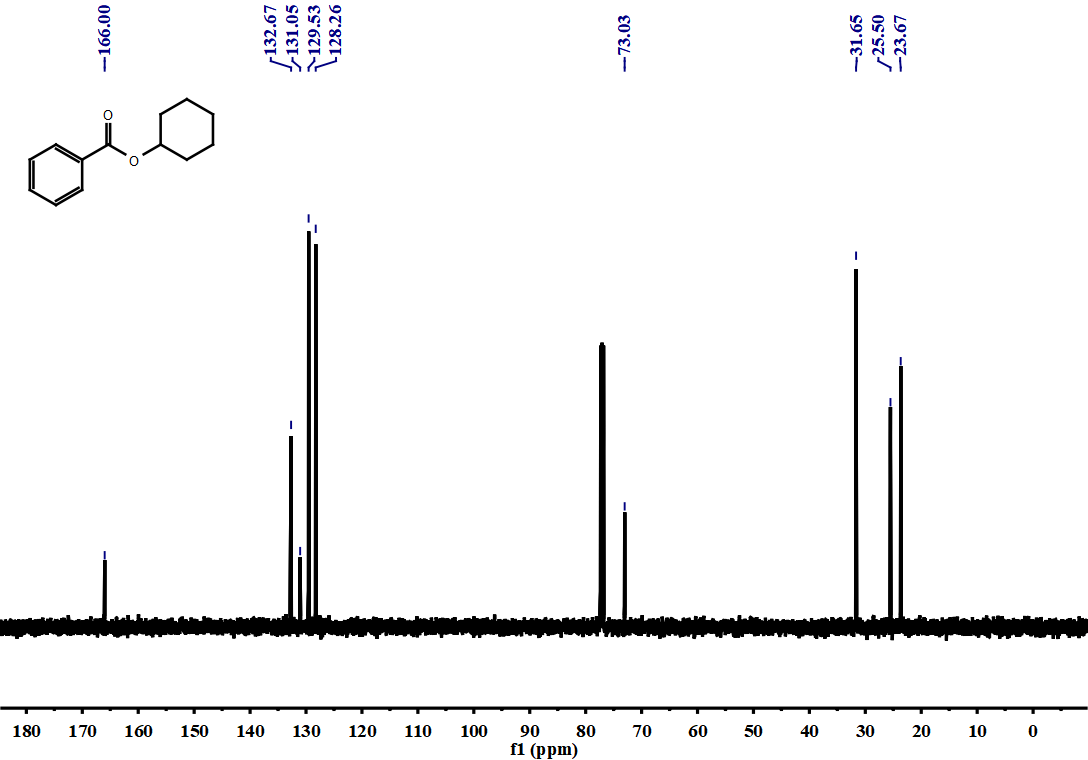


**Figure S50.** ^1^H NMR (top) and ^13^C NMR (bottom) of **45** (CDCl_3_ as the solvent).


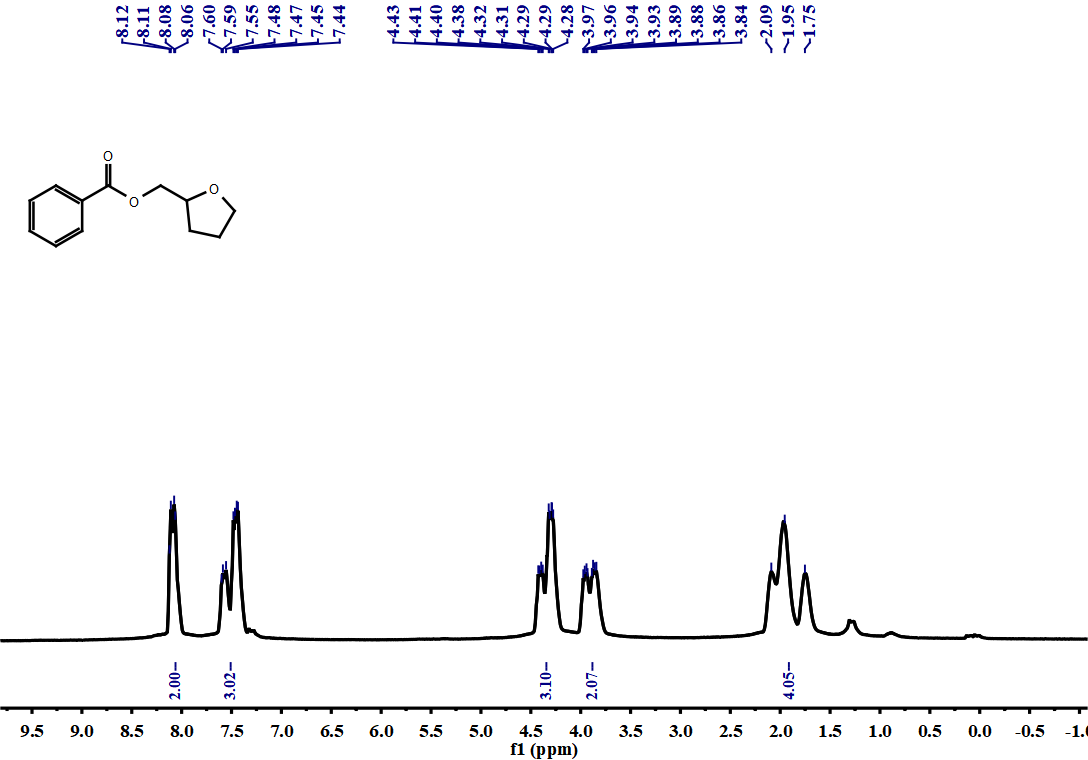


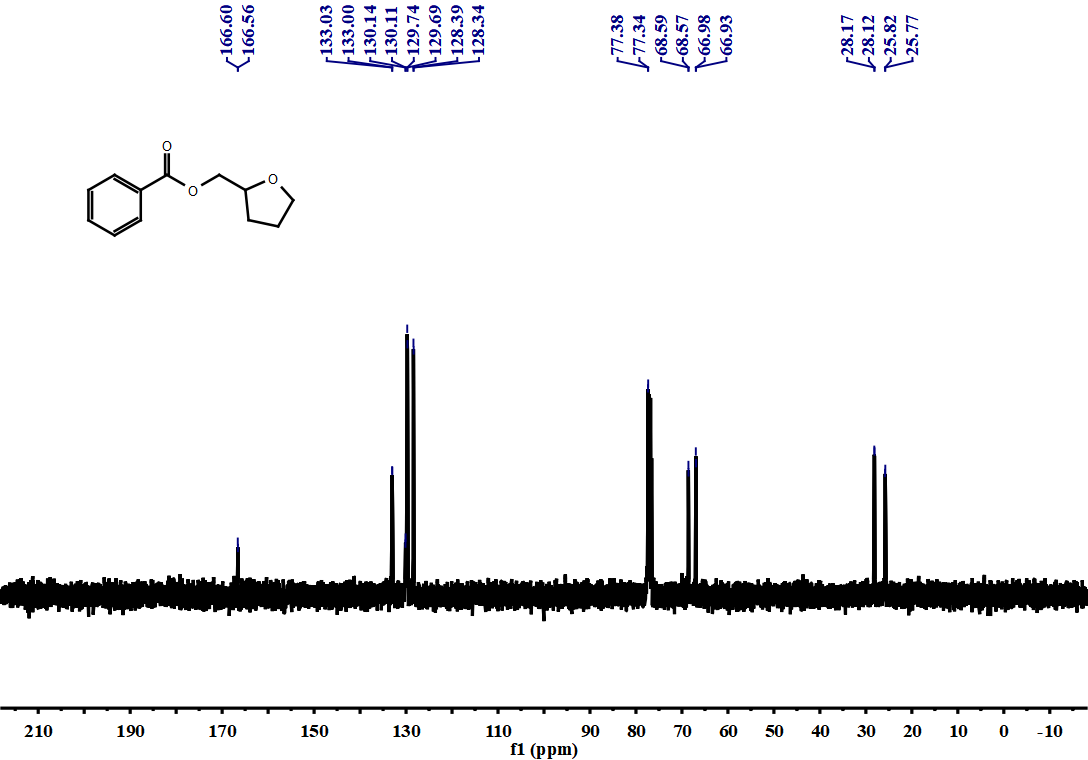


**Figure S51.** ^1^H NMR (top) and ^13^C NMR (bottom) of **46** (CDCl_3_ as the solvent).


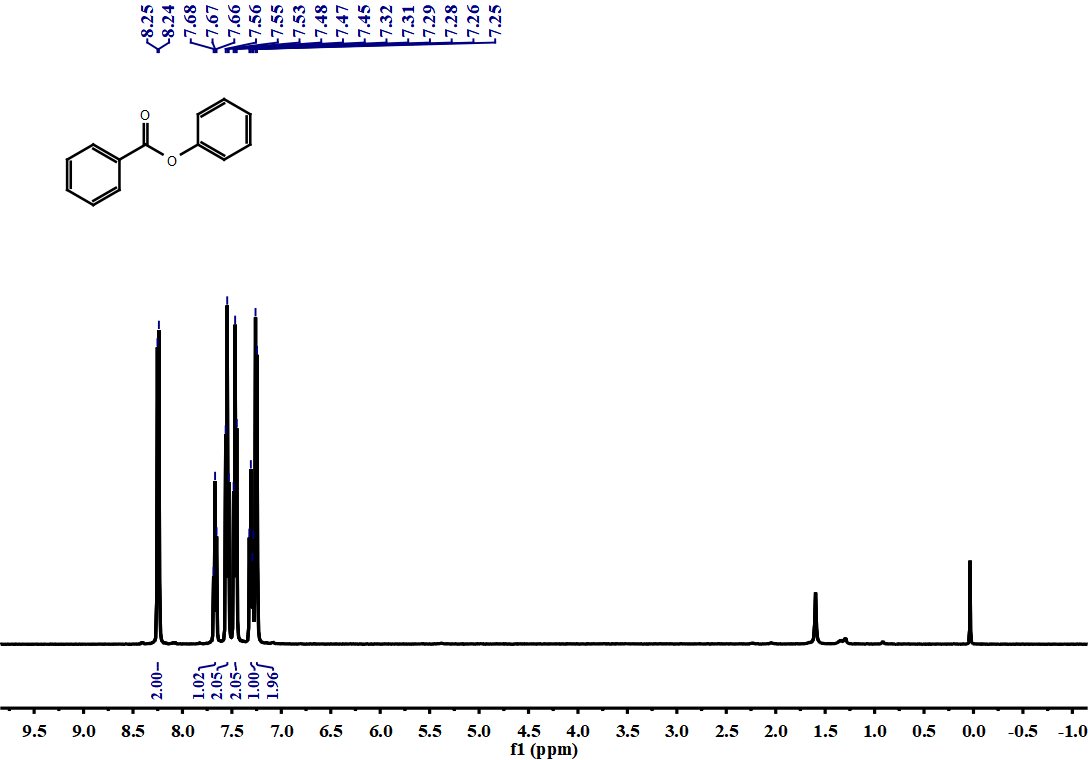


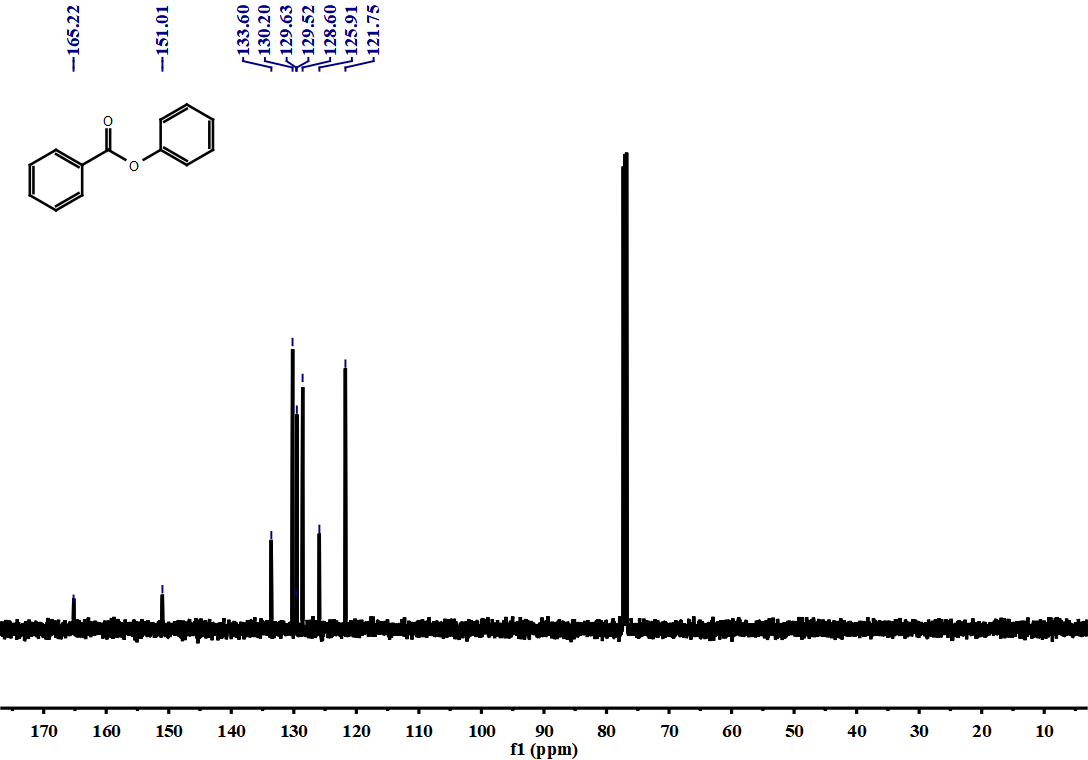


**Figure S52.** ^1^H NMR (top) and ^13^C NMR (bottom) of **47** (CDCl_3_ as the solvent).


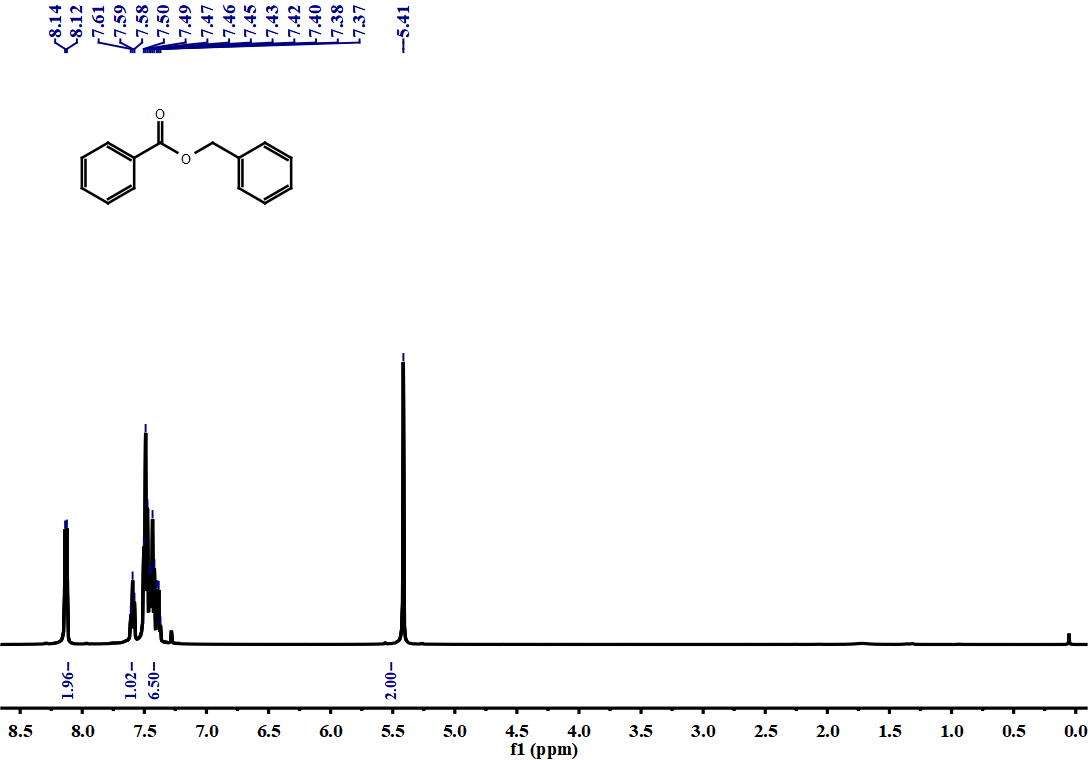


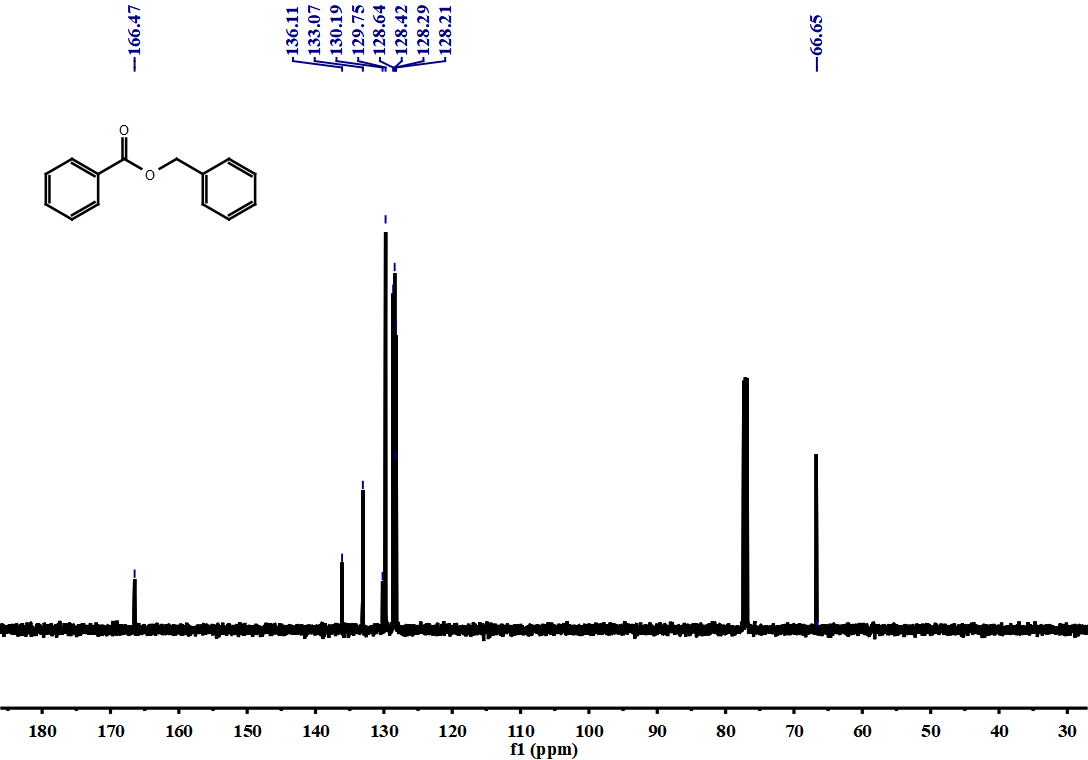


**Figure S53.** ^1^H NMR (top) and ^13^C NMR (bottom) of **48** (CDCl_3_ as the solvent).


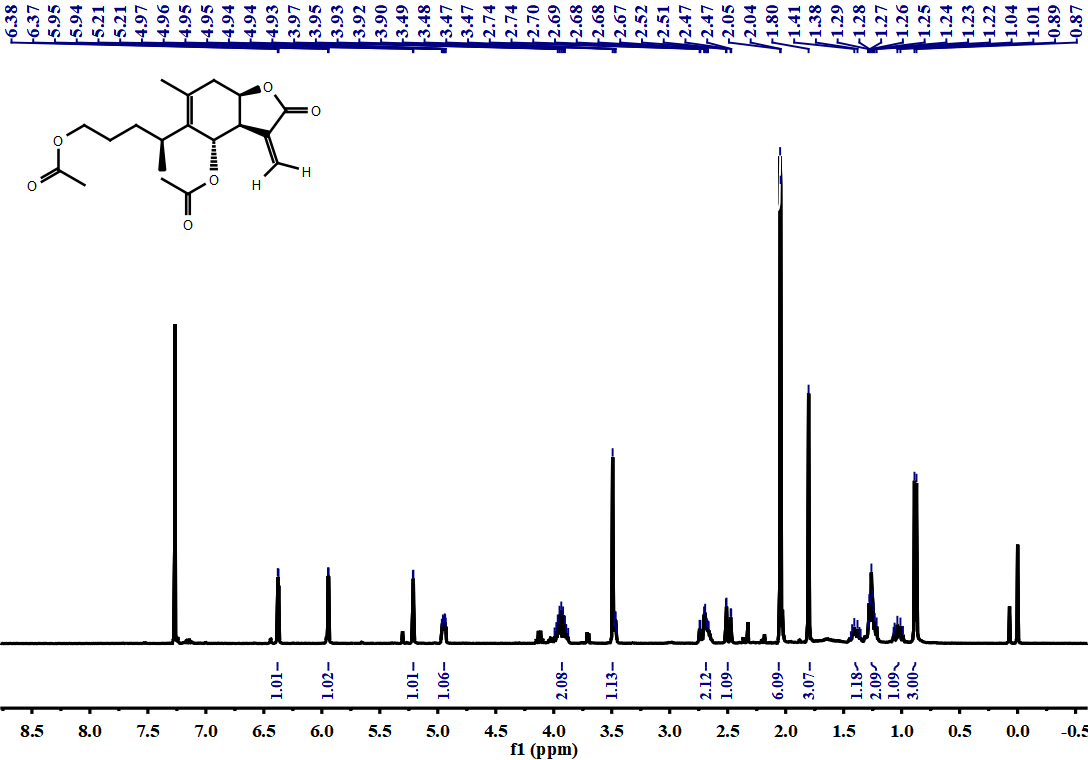


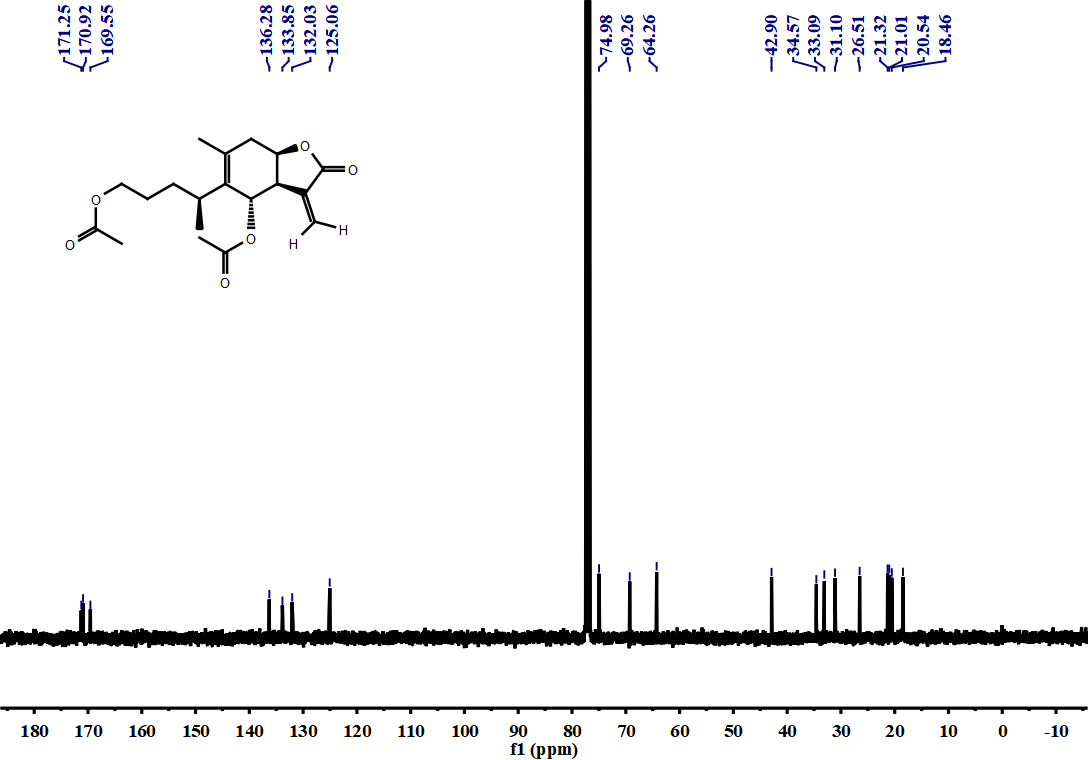


**Figure S54.** ^1^H NMR (top) and ^13^C NMR (bottom) of **49** (CDCl_3_ as the solvent).

**Supplementary Tables**

**Table S1. The effect of additive on the oxidative esterification of benzylic alcohol with methanol.^a^**

| **Entry** | **Additive (equiv)** | **Conv. (%)** | **Yield (%)^d^** |
| --- | --- | --- | --- |
| 1 | Na_2_SO_3_ | 21 | 15 |
| 2 | Na_2_SO_4_ | <5 | Trace |
| **3** | **NaCl** | **80** | **62** |
| 4 | NaHCO_3_ | <5 | Trace |
| 5 | Na_2_CO_3_ | <5 | Trace |
| 6 | NaOAc | <5 | Trace |
| 7 | KCl | >99 | 92 |
| 8^b^ | KCl | >99 | 82 |
| **9^c^** | **KCl** | **>99** | **97** |
| 10 | KF | <5 | Trace |
| 11 | KBr | <5 | Trace |
| 12 | KI | <5 | Trace |
| ^a^Reaction conditions: Cat. **1** (1.0 mol%), benzyl alcohol (1.0 mmol), 30% H_2_O_2_ (2.0 equiv), CH_3_OH (2 mL), 65^o^C, 24 hours. ^d^0.05 equiv. ^c^0.2equiv. ^d^Substrate yields were determined by GC-MS analysis. | | | |

**Table S2. The effect of** [**positive**](javascript:;) [**ion**](javascript:;)**of additive on the oxidative esterification of benzylic alcohol with methanol.^a^**

| **Entry** | **Additive (equiv)** | **Conv. (%)^b^** | **Yield (%)^b^** |
| --- | --- | --- | --- |
| **1** | **KCl** | **>99** | **97** |
| 2 | LiCl | 48 | 39 |
| 3 | CaCl_2_ | 41 | 31 |
| 4 | ZnCl_2_ | 53 | 42 |
| 5 | MgCl_2_ | 76 | 57 |
| 6 | NH_4_Cl | 43 | 31 |
| 7 | CsCl | 86 | 73 |
| ^a^Reaction conditions: Cat. **1** (1.0 mol%), benzyl alcohol (1.0 mmol), 30% H_2_O_2_ (2.0 equiv), CH_3_OH (2 mL), 70^o^C, 36 hours.  ^b^Substrate yields were determined by GC-Ms analysis. | | | |

**Table S3.** **Impact of reaction parameters** **on the oxidative esterification of benzylic alcohol with methanol.^a^**

| **Entry** | **Oxidant (equiv)** | **T (^o^C)** | **Time (hour)** | **Conv. (%)** | **Yield** **(%)^d^** |
| --- | --- | --- | --- | --- | --- |
| 1 | H_2_O_2_ | 50 | 24 | 23 | 15 |
| 2 | H_2_O_2_ | 60 | 24 | 30 | 21 |
| 3 | H_2_O_2_ | 65 | 24 | 38 | 31 |
| 4 | H_2_O_2_ | 70 | 24 | 45 | 36 |
| 5 | H_2_O_2_ | 80 | 24 | 40 | 31 |
| 6 | H_2_O_2_ | 70 | 12 | 15 | <10 |
| 7 | H_2_O_2_ | 70 | 36 | 95 | 82 |
| 8^b^ | H_2_O_2_ (3.0) | 70 | 36 | 99 | 90 |
| **9^c^** | **H_2_O_2_(4.0)** | **70** | **36** | **>99** | **97** |
| 10 | TBHP | 70 | 36 | 17 | 9.0 |
| 11 | Ditbutylperoxide | 70 | 36 | 38 | 31 |
| 12 | O_2_ | 70 | 36 | 29 | trace |
| ^a^Reaction conditions: Cat. **1** (1.0 mol%), benzyl alcohol (1.0 mmol), 30% H_2_O_2_ (2.0 equiv), CH_3_OH (2 mL), KCl (0.2 equiv). ^b^30% H_2_O_2_ (3.0 equiv). ^c^30% H_2_O_2_ (4.0 equiv). ^d^Substrate yields were determined by GC-Ms analysis. | | | | | |

**Table S4. Investigation the influence of the metal center of catalyst 1 on the** **oxidative esterification of benzylic alcohol with methanol.^a^**

| **Entry** | **Catalyst** | **Conv. (%)^b^** | **Yield (%)^b^** |
| --- | --- | --- | --- |
| 1 | - | - | - |
| **2** | **Co** | **>99** | **97** |
| 3 | Fe | 74 | 66 |
| 4 | Ni | 65 | 58 |
| 5 | Al | 98 | 93 |
| 6 | Zn | 90 | 82 |
| 7 | Cr | 83 | 72 |
| ^a^ Reaction conditions: Catalyst (1.0 mol%), benzyl alcohol (1.0 mmol), 30% H_2_O_2_ (2.0 equiv), KCl (0.2 equiv), CH_3_OH (2 mL), 70^o^C, 36 hours. ^b^Substrate yields were determined by GC-Ms analysis. | | | |

**Table S5. Crystal data and structure refinement for CoMo_6_**/Cl.

| Identification code | CoMo_6_Cl |
| --- | --- |
| Empirical formula | C_48_H_115_ClCoMo_6_N_3_O_24_ |
| Formula weight | 1788.44 |
| Temperature/K | 100 |
| Crystal system | monoclinic |
| Space group | *C2/c* |
| a/Å | 26.1667(4) |
| b/Å | 16.5284(2) |
| c/Å | 32.6499(4) |
| α/° | 90 |
| β/° | 99.6138(13) |
| γ/° | 90 |
| Volume/Å^3^ | 13922.6(3) |
| Z | 8 |
| ρ_calc_g/cm^3^ | 1.706 |
| μ/mm^‑1^ | 1.388 |
| F(000) | 7296.0 |
| Crystal size/mm^3^ | 0.2 × 0.2 × 0.2 |
| Radiation | MoKα (λ = 0.71073) |
| 2Θ range for data collection/° | 6.73 to 59 |
| Index ranges | -35 ≤ h ≤ 34, -22 ≤ k ≤ 22, -41 ≤ l ≤ 45 |
| Reflections collected | 64474 |
| Independent reflections | 17009 [R_int_ = 0.0411, R_sigma_ = 0.0452] |
| Data/restraints/parameters | 17009/7/788 |
| Goodness-of-fit on F^2^ | 1.067 |
| Final R indexes [I>=2σ (I)] | R_1_ = 0.0325, wR_2_ = 0.0619 |
| Final R indexes [all data] | R_1_ = 0.0486, wR_2_ = 0.0679 |
| Largest diff. peak/hole / e Å^-3^ | 0.68/-0.76 |

**Table S6**. Hydrogen bonds in the dimeric cluster.

| D-H | d(D-H) | d(H...A) | <DHA | d(D..A) | A |
| --- | --- | --- | --- | --- | --- |
| O6-H6 | 0.850 | 1.932 | 165.00 | 2.762 | O23 [ -x+1/2, -y+1/2, -z+1 ] |
| O5-H5 | 0.850 | 2.430 | 138.75 | 3.120 | O23 [ -x+1/2, -y+1/2, -z+1 ] |
| O1-H1 | 0.850 | 2.350 | 153.94 | 3.136 | Cl1 |
| O11-H11 | 0.850 | 1.944 | 158.00 | 2.751 | O22 [ -x+1/2, -y+1/2, -z+1 ] |
| O2-H2 | 0.850 | 2.305 | 152.67 | 3.085 | Cl1 |
| O3-H3 | 0.850 | 2.219 | 150.54 | 2.989 | Cl1 |
| O4-H4 | 0.850 | 2.374 | 142.48 | 3.092 | O22 [ -x+1/2, -y+1/2, -z+1 ] |
